# Supplementary material for: Affinity and cooperativity modulate ternary complex formation to drive targeted protein degradation
Source: Nat Commun. 2023 Jul 13;14:4177. doi: 10.1038/s41467-023-39904-5 (PMC10344917; doi:10.1038/s41467-023-39904-5)
Supplement: Supplementary file 1 — Supplementary Information [file 41467_2023_39904_MOESM1_ESM.docx]

**Supplementary Information**

**Affinity and Cooperativity Modulate Ternary Complex Formation to**

**Drive Targeted Protein Degradation**

Ryan P. Wurz^1^, Huan Rui^1^, Ken Dellamaggiore^1^, Sudipa Ghimire-Rijal^1^, Kaylee Choi^2^, Kate Smither^1^, Albert Amegadzie^1^, Ning Chen^1^, Xiaofen Li^1^, Abhisek Banerjee^3^, Qing Chen^1^, Dane Mohl^1*^ and Amit Vaish^1*^

^1^Amgen Research, Amgen Inc., Thousand Oaks, United States

^2^Amgen Research, Amgen Inc., South San Francisco, United States

^3^Syngene Amgen Research Center (SARC), Bangalore, India.

*email: [avaish@amgen.com](mailto:avaish@amgen.com), dmohl@amgen.com

**Supplementary Note 1:**

**Information on Compounds Syntheses and Characterization**

**Synthesis of SMARCA2-VHL PROTACs**

**(2*S*,4*R*)-1-((*S*)-2-Amino-3,3-dimethylbutanoyl)-4-hydroxy-*N*-((*S*)-1-(4-(4-methylthiazol-5-yl)phenyl)ethyl)pyrrolidine-2-carboxamide bis(hydrochloride) salt (Intermediate 1).**

**Step 1: *tert*-Butyl (*S*)-(1-(4-bromophenyl)ethyl)carbamate.** To a solution of (*S*)-1-(4-bromophenyl)ethan-1-amine (50.0 g, 250 mmol) and Et_3_N (27.8 g, 275 mmol) in DCM (350 mL) was added Boc_2_O (57.3 g, 262 mmol) drop-wise at 0 °C and the mixture was stirred at 20 °C for 1 h. The mixture washed with water (2 x 150 mL), brine (150 mL), dried over Na_2_SO_4_, filtered and concentrated to give crude *tert*-butyl (*S*)-(1-(4-bromophenyl)ethyl)carbamate (65 g) as white solid. ^1^H NMR (400 MHz, *CDCl_3_*) δ ppm 7.46-7.43 (m, 2H), 7.19-7.16 (m, 2H), 4.74 (br s, 2H), 1.56 (s, 2H), 1.53 (s, 1H), 1.41 (s, 13H).

**Step 2: *tert*-Butyl (*S*)-(1-(4-(4-methylthiazol-5-yl)phenyl)ethyl)carbamate.** *tert*-Butyl (*S*)-(1-(4-bromophenyl)ethyl)carbamate (11.0 g, 36.6 mmol), 4-methylthiazole (7.27 g, 73.3 mmol), KOAc (7.19 g, 73.3 mmol) and Pd(OAc)_2_ (82 mg, 366 μmol) in DMA (30 mL) was degassed and then heated to 90 °C for 12 h under nitrogen. The reaction mixture was diluted with water (80 mL) and extracted with DCM (3 x 80 mL). The combined organic layers were washed with brine (2 x 80 mL), dried over Na_2_SO_4,_ filtered and concentrated under reduced pressure (rotary evaporator). The crude residue was purified by column chromatography on silica gel eluting with a gradient of 30:1 to 3:1 petroleum ether/EtOAc to afford *tert*-butyl (*S*)-(1-(4-(4-methylthiazol-5-yl)phenyl)ethyl)carbamate (19.0 g, 59.7 mmol, 41% yield) as white solid.

**Step 3: (*S*)-1-(4-(4-Methylthiazol-5-yl)phenyl)ethan-1-amine hydrochloride.** A mixture of *tert*-butyl (*S*)-(1-(4-(4-methylthiazol-5-yl)phenyl)ethyl)carbamate (19.0 g, 56.7 mmol) and HCl/MeOH (4 M, 142 mL) in MeOH (140 mL) was stirred at 25 °C for 1 h. The mixture was concentrated under reduced pressure (rotary evaporator) to afford crude (*S*)-1-(4-(4-methylthiazol-5-yl)phenyl)ethan-1-amine hydrochloride (18.0 g) as white solid.

**Step 4: Methyl (2*S*,4*R*)-1-((*S*)-2-((*tert*-butoxycarbonyl)amino)-3,3-dimethylbutanoyl)-4-hydroxypyrrolidine-2-carboxylate.** To a solution of (*S*)-2-((*tert*-butoxycarbonyl)amino)-3,3-dimethylbutanoic acid (15.7 g, 86.5 mmol) and HATU (36.2 g, 95.1 mmol) in DCM (140 mL) was added methyl (2*S*,4*R*)-4-hydroxypyrrolidine-2-carboxylate (20.0 g, 86.5 mmol) and Et_3_N (30.6 g, 302 mmol) at 0 °C. The mixture was stirred at 25 °C for 18 h then the reaction mixture was quenched with water (400 mL) at 25 °C, and extracted with EtOAc (3 x 200 mL). The combined organic layers were washed with 5% citric acid  (2 x 140 mL), sat’d NaHCO_3_ solution (2 x 140 mL), and brine (2 x 140 mL) and dried over Na_2_SO_4_, filtered and concentrated under reduced pressure (rotary evaporator). The crude residue was purified by column chromatography on silica gel eluting with a gradient of 30:1 to 3:1 petroleum ether/EtOAc to afford methyl (2*S*,4*R*)-1-((*S*)-2-((*tert*-butoxycarbonyl)amino)-3,3-dimethylbutanoyl)-4-hydroxypyrrolidine-2-carboxylate (24.0 g, 67.0 mmol, 77% yield) as white solid. ^1^H NMR (400 MHz, *CDCl_3_*) δ ppm 5.27 (d, *J* = 9.2 Hz, 1H), 4.67 (t, *J* = 8.4 Hz, 1H), 4.50 (br s, 1H), 4.18 (d, *J* = 9.6 Hz, 1H), 4.11 (d, *J* = 7.2 Hz, 1H), 3.76 (s, 4H), 3.0 (br s, 1H), 2.37-2.32 (m, 1H), 1.40 (s, 9H), 1.1 (s, 9H), 1.0 (s,1H).

**Step 5: (2*S*,4*R*)-1-((*S*)-2-((*tert*-Butoxycarbonyl)amino)-3,3-dimethylbutanoyl)-4-hydroxypyrrolidine-2-carboxylic acid.** A mixture of methyl (2*S*,4*R*)-1-((*S*)-2-((*tert*-butoxycarbonyl)amino)-3,3-dimethylbutanoyl)-4-hydroxypyrrolidine-2-carboxylate (7.00 g, 19.53 mmol), LiOH monohydrate (2.34 g, 97.65 mmol) in THF (34 mL) and water (10 mL) was purged with nitrogen, and the mixture was stirred at 25 °C for 18 h. The THF was removed under reduced pressure (rotary evaporator) and the residue was diluted with ice water (35 mL) and the pH was slowly adjusted to pH 2-3 with 3 N HCl (ca. 20 mL). The resulting suspension was filtered and washed with water (2 x 30 mL) to afford (2*S*,4*R*)-1-((*S*)-2-((*tert*-butoxycarbonyl)amino)-3,3-dimethylbutanoyl)-4-hydroxypyrrolidine-2-carboxylic acid (5.36 g, 15.6 mmol, 80% yield) as white solid.

**Step 6: *tert*-Butyl ((*S*)-1-((2*S*,4*R*)-4-hydroxy-2-(((*S*)-1-(4-(4-methylthiazol-5-yl)phenyl)ethyl)carbamoyl)pyrrolidin-1-yl)-3,3-dimethyl-1-oxobutan-2-yl)carbamate.** A mixture of (*S*)-1-(4-(4-methylthiazol-5-yl)phenyl)ethan-1-amine hydrochloride (4.90 g, 19.2 mmol), (2*S*,4*R*)-1-((*S*)-2-((*tert*-butoxycarbonyl)amino)-3,3-dimethylbutanoyl)-4-hydroxypyrrolidine-2-carboxylic acid (7.40 g, 19.2 mmol), HATU (8.00 g, 21.2 mmol), Et_3_N (9.3 mL, 67.3 mmol) in DCM (100 mL) was purged with nitrogen, and then the mixture was stirred at 0-25 °C for 20 h. The reaction mixture was quenched with water (50 mL) and extracted with EtOAc (3 x 100 mL). The combined organic layers were washed with 5% citric acid (2 x 70 mL), sat’d NaHCO_3_ solution (2 x 70 mL), and brine (2 x 70 mL) and dried over Na_2_SO_4_, filtered and concentrated under reduced pressure (rotary evaporator). The crude residue was purified by reverse phase column chromatography to afford *tert*-butyl ((*S*)-1-((2*S*,4*R*)-4-hydroxy-2-(((*S*)-1-(4-(4-methylthiazol-5-yl)phenyl)ethyl)carbamoyl)pyrrolidin-1-yl)-3,3-dimethyl-1-oxobutan-2-yl)carbamate (5.90 g, 10.2 mmol, 53% yield) as white solid.

**Step 7: (2*S*,4*R*)-1-((*S*)-2-Amino-3,3-dimethylbutanoyl)-4-hydroxy-*N*-((*S*)-1-(4-(4-methylthiazol-5-yl)phenyl)ethyl)pyrrolidine-2-carboxamide bis(hydrochloride) salt (Intermediate 1).** A mixture of ((*S*)-1-((2*S*,4*R*)-4-hydroxy-2-(((*S*)-1-(4-(4-methylthiazol-5-yl)phenyl)ethyl)carbamoyl)pyrrolidin-1-yl)-3,3-dimethyl-1-oxobutan-2-yl)carbamate (4.10 g, 7.50 mmol), HCl/MeOH (4 M, 18.8 mL) in MeOH (30 mL) was purged with nitrogen, and then the mixture was stirred at 25 °C for 2 h. The mixture was concentrated to give the crude product. The residue was purified by reverse phase column chromatography to afford (2*S*,4*R*)-1-((*S*)-2-amino-3,3-dimethylbutanoyl)-4-hydroxy-*N*-((*S*)-1-(4-(4-methylthiazol-5-yl)phenyl)ethyl)pyrrolidine-2-carboxamide bis(hydrochloride) salt (**Intermediate 1**, 5.10 g, 9.80 mmol, 92% yield) as yellow solid. ^1^H NMR (400 MHz, *DMSO-d_6_*) δ ppm 9.16 (s, 1H), 8.65 (d, 1H), 8.17 (s, 3H), 7.48-7.44 (m, *J* = 8.7, 3, 2H), 7.41-7.38 (m, 2H), 5.96 (br s, 1H), 4.91 (t, *J* = 7.2, 1H), 4.54 (t, *J* = 8.0 Hz, 1H), 4.30 (s, 1H), 3.74 (d, *J* = 7.2, 1H), 3.50-3.46 (m, 1H), 2.47 (s, 3H), 2.11 (t, *J* = 4.8 Hz, 1H), 1.77-1.72 (m, 1H), 2.35-2.38 (m, 1 H), 1.37 (s, *J* = 8.0 Hz, 3H), 1.02 (s, 9H).

**2-(4-(3-Amino-6-(2-hydroxyphenyl)pyridazin-4-yl)piperazin-1-yl)-*N*-((*S*)-1-((2*S*,4*R*)-4-hydroxy-2-(((*S*)-1-(4-(4-methylthiazol-5-yl)phenyl)ethyl)carbamoyl)pyrrolidin-1-yl)-3,3-dimethyl-1-oxobutan-2-yl)isonicotinamide (Compound 1).**

**Step 1: *tert*-Butyl 4-(4-(((*S*)-1-((2*S*,4*R*)-4-hydroxy-2-(((*S*)-1-(4-(4-methylthiazol-5-yl)phenyl)ethyl)carbamoyl)pyrrolidin-1-yl)-3,3-dimethyl-1-oxobutan-2-yl)carbamoyl)pyridin-2-yl)piperazine-1-carboxylate.** A mixture of 2-(4-[(*tert*-butoxy)carbonyl]piperazin-1-yl)pyridine-4-carboxylic acid (1.73 g, 5.63 mmol, Enamine), (2*S*,4*R*)-1-((*S*)-2-amino-3,3-dimethylbutanoyl)-4-hydroxy-*N*-((*S*)-1-(4-(4-methylthiazol-5-yl)phenyl)ethyl)pyrrolidine-2-carboxamide hydrochloride (**Intermediate 1**, 2.71 g, 5.63 mmol), bromotri(pyrrolidin-1-yl)phosphonium hexafluorophosphate(V) (2.89 g, 6.19 mmol, Sigma-Aldrich Corporation), and Et_3_N (2.4 mL, 16.9 mmol) in DCM (20 mL) was stirred at r.t. for 2 h. The reaction mixture was diluted with water and extracted with DCM (2x). The organic layer was concentrated under reduced pressure (rotary evaporator) and the crude residue purified by column chromatography on silica gel eluting with a gradient of 0-10% MeOH in DCM to afford the title compound (3.19 g, 4.35 mmol, 77% yield). *m/z* (ESI, +ve ion): 734.3 (M+H)^+^.

**Step 2: 2-(4-(3-Amino-6-chloropyridazin-4-yl)piperazin-1-yl)-*N*-((*S*)-1-((2*S*,4*R*)-4-hydroxy-2-(((*S*)-1-(4-(4-methylthiazol-5-yl)phenyl)ethyl)carbamoyl)pyrrolidin-1-yl)-3,3-dimethyl-1-oxobutan-2-yl)isonicotinamide.** TFA (7.54 mL, 97.80 mmol) was added to a solution of *tert*-butyl 4-(4-(((*S*)-1-((2*S*,4*R*)-4-hydroxy-2-(((*S*)-1-(4-(4-methylthiazol-5-yl)phenyl)ethyl)carbamoyl)pyrrolidin-1-yl)-3,3-dimethyl-1-oxobutan-2-yl)carbamoyl)pyridin-2-yl)piperazine-1-carboxylate (3.59 g, 4.89 mmol) in DCM (20 mL). The solution was stirred at r.t. for 2 h and concentrated under reduced pressure (rotary evaporator). The crude residue was dissolved in DMSO (10 mL) and treated with 3-amino-4-bromo-6-chloropyridazine (1.02 g, 4.89 mmol, CombiBlocks) and DIPEA (8.54 mL, 48.9 mmol) and heated to 100 °C for 18 h. The reaction mixture was cooled, diluted with water and extracted with DCM (2x). The organic layer was concentrated under reduced pressure (rotary evaporator) and the crude residue purified by column chromatography on silica gel eluting with a gradient of 0-20% MeOH in DCM to afford the title compound (2.02 g, 2.65 mmol, 54% yield). *m/z* (ESI, +ve ion): 761.1/762.3 (M+H)^+^.

**Step 3: 2-(4-(3-Amino-6-(2-hydroxyphenyl)pyridazin-4-yl)piperazin-1-yl)-*N*-((*S*)-1-((2*S*,4*R*)-4-hydroxy-2-(((*S*)-1-(4-(4-methylthiazol-5-yl)phenyl)ethyl)carbamoyl)pyrrolidin-1-yl)-3,3-dimethyl-1-oxobutan-2-yl)isonicotinamide (Compound 1).** A mixture of 2-(4-(3-amino-6-chloropyridazin-4-yl)piperazin-1-yl)-*N*-((*S*)-1-((2*S*,4*R*)-4-hydroxy-2-(((*S*)-1-(4-(4-methylthiazol-5-yl)phenyl)ethyl)carbamoyl)pyrrolidin-1-yl)-3,3-dimethyl-1-oxobutan-2-yl)isonicotinamide (1.60 g, 2.10 mmol), 2-hydroxybenzeneboronic acid (0.58 g, 4.20 mmol, Oakwood Products), (2-dicyclohexylphosphino-2',4',6'-triisopropyl-1,1'-biphenyl)[2-(2'-amino-1,1'-biphenyl)]palladium(II) methanesulfonate (0.18 g, 0.21 mmol, Sigma-Aldrich Corporation), and sodium carbonate (0.67 g, 6.30 mmol) in 1,4-dioxane (15 mL) and water (10 mL) was heated to 110 °C for 1 h. The reaction mixture was diluted with water and extracted with DCM (3x). The organic layer was concentrated under reduced pressure (rotary evaporator) and the crude residue purified by column chromatography on silica gel eluting with a gradient of 0-20% MeOH in DCM. The product was further purified via preparative SFC using a Princeton MSA column (250 x 21 mm, 5 mm) with a mobile phase of 65% liquid CO_2_ and 35% MeOH using a flowrate of 70 mL/min to afford **Compound 1** (0.70 g, 0.86 mmol, 41% yield). ^1^H NMR (400 MHz, DMSO-*d_6_*,) δ ppm 14.21 (br s, 1H), 8.99 (br s, 1H), 8.41 (br d, 1H, *J*=7.0 Hz), 8.2-8.4 (m, 2H), 7.94 (br d, 1H, *J*=6.8 Hz), 7.58 (br s, 1H), 7.3-7.5 (m, 4H), 7.2-7.3 (m, 2H), 7.0-7.1 (m, 1H), 6.90 (br d, 2H, *J*=6.8 Hz), 6.40 (br s, 2H), 5.1-5.2 (m, 1H), 4.9-5.0 (m, 1H), 4.80 (br d, 1H, *J*=9.1 Hz), 4.47 (br t, 1H, *J*=7.6 Hz), 4.33 (br s, 2H), 3.83 (br s, 4H), 3.68 (br s, 2H), 3.2-3.3 (m, 4H), 2.5-2.5 (m, 2H), 1.9-2.1 (m, 1H), 1.82 (br s, 1H), 1.39 (br d, 3H, *J*=6.4 Hz), 1.05 (br s, 9H). *m/z* (ESI, +ve ion): 819.3 (M+H)^+^.

**6-(4-(3-Amino-6-(2-hydroxyphenyl)pyridazin-4-yl)piperazin-1-yl)-*N*-((*S*)-1-((2*S*,4*R*)-4-hydroxy-2-(((*S*)-1-(4-(4-methylthiazol-5-yl)phenyl)ethyl)carbamoyl)pyrrolidin-1-yl)-3,3-dimethyl-1-oxobutan-2-yl)picolinamide (Compound 2).**

**Step 1: *tert*-Butyl 6-(4-(3-amino-6-chloropyridazin-4-yl)piperazin-1-yl)picolinate.** To a solution of *tert*-butyl 6-(piperazin-1-yl)picolinate (2.00 g, 7.59 mmol, Aurum Pharmatech) in DMSO (25 mL) was added 3-amino-4-bromo-6-chloropyridazine (1.90 g, 9.11 mmol, CombiBlocks), followed by DIPEA (2.94 g, 22.78 mmol) dropwise. The reaction mixture was stirred at 100 °C for 18 h. The reaction mixture was diluted with water and extracted with EtOAc (100 mL). The aqueous layer was separated and back-extracted with EtOAc (3x). The organic layer was dried over Na_2_SO_4_ and concentrated and purified using column chromatography on silica gel eluting with 0-60% EtOH/EtOAc (3:1) in heptane to afford *tert*-butyl 6-(4-(3-amino-6-chloropyridazin-4-yl)piperazin-1-yl)picolinate (2.14 g, 5.47 mmol, 72% yield) as tan solid.

**Step 2: *tert*-Butyl 6-(4-(3-amino-6-(2-hydroxyphenyl)pyridazin-4-yl)piperazin-1-yl)picolinate.** A mixture of *tert*-butyl 6-(4-(3-amino-6-chloropyridazin-4-yl)piperazin-1-yl)picolinate (0.53 g, 1.36 mmol), 2-hydroxybenzeneboronic acid (0.28 g, 2.04 mmol, Oakwood Products), (2-dicyclohexylphosphino-2',4',6'-triisopropyl-1,1'-biphenyl)[2-(2'-amino-1,1'-biphenyl)]palladium(II) methanesulfonate (0.12 g, 0.14 mmol, Sigma-Aldrich Corporation), and sodium carbonate (0.36 g, 3.40 mmol) in 1,4-dioxane (5 mL) and water (2 mL) was purged with argon, capped and heated to 110 °C for 2 h. The reaction mixture was diluted with water and extracted with EtOAc (2x). The combined organic layers was concentrated and the residue purified by column chromatography on silica gel eluting with 0-80% EtOH/EtOAc (3:1) in heptane to afford *tert*-butyl 6-(4-(3-amino-6-(2-hydroxyphenyl)pyridazin-4-yl)piperazin-1-yl)picolinate (0.47 g, 1.05 mmol, 77% yield). *m/z* (ESI, +ve ion): 449.2 (M+H)^+^.

**Step 3: 6-(4-(3-Amino-6-(2-hydroxyphenyl)pyridazin-4-yl)piperazin-1-yl)-*N*-((*S*)-1-((2*S*,4*R*)-4-hydroxy-2-(((*S*)-1-(4-(4-methylthiazol-5-yl)phenyl)ethyl)carbamoyl)pyrrolidin-1-yl)-3,3-dimethyl-1-oxobutan-2-yl)picolinamide (Compound 2).** TFA (1.4 mL, 18.5 mmol) was added to a solution of *tert*-butyl 6-(4-(3-amino-6-(2-hydroxyphenyl)pyridazin-4-yl)piperazin-1-yl)picolinate (0.17 g, 0.37 mmol) in DCM (2 mL). The reaction was stirred at r.t. for 2 h and concentrated to dryness under reduced pressure (rotary evaporator). The crude residue was treated with (2*S*,4*R*)-1-((*S*)-2-amino-3,3-dimethylbutanoyl)-4-hydroxy-*N*-((*S*)-1-(4-(4-methylthiazol-5-yl)phenyl)ethyl)pyrrolidine-2-carboxamide (**Intermediate 1**, 0.17 g, 0.37 mmol), bromotri(1-pyrrolidinyl)phosphonium hexafluorophosphate (0.26 g, 0.56 mmol, Sigma-Aldrich Corporation), and TEA (0.26 mL, 1.85 mmol) in DCM (2 mL). The resulting solution was stirred at r.t. for 2 h, diluted with water and extracted with EtOAc (2x). The combined organic layers was concentrated under reduced pressure (rotary evaporator) and the crude residue was purified by column chromatography on silica gel, eluting with a gradient of 0-20% MeOH in DCM to afford 6-(4-(3-amino-6-(2-hydroxyphenyl)pyridazin-4-yl)piperazin-1-yl)-*N*-((*S*)-1-((2*S*,4*R*)-4-hydroxy-2-(((*S*)-1-(4-(4-methylthiazol-5-yl)phenyl)ethyl)carbamoyl)pyrrolidin-1-yl)-3,3-dimethyl-1-oxobutan-2-yl)picolinamide (**Compound 2**, 29 mg, 0.035 mmol, 10% yield). ^1^H NMR (400 MHz, *CDCl_3_*) δ ppm 8.64 (s, 1H), 8.53 (br d, *J*=8.50 Hz, 1H), 7.48-7.70 (m, 4H), 7.27-7.39 (m, 6H), 7.05 (br d, *J*=8.09 Hz, 1H), 6.85-6.94 (m, 2H), 4.99-5.20 (m, 3H), 4.77 (br t, *J*=7.57 Hz, 1H), 4.68 (br d, *J*=8.50 Hz, 1H), 4.54 (br s, 1H), 4.24 (br d, *J*=11.20 Hz, 1H), 3.75 (br s, 4H), 3.67 (br d, *J*=10.78 Hz, 2H), 3.24 (br s, 4H), 2.98-3.13 (m, 1H), 2.49 (s, 4H), 1.99-2.14 (m, 1H), 1.48 (br d, *J*=6.84 Hz, 3H), 1.15 (s, 9H). *m/z* (ESI, +ve ion): 819.2 (M+H)^+^.

**(2*S*,4*R*)-1-((*S*)-2-(3-(4-(3-Amino-6-(2-hydroxyphenyl)pyridazin-4-yl)piperazin-1-yl)benzamido)-3,3-dimethylbutanoyl)-4-hydroxy-*N*-((*S*)-1-(4-(4-methylthiazol-5-yl)phenyl)ethyl)pyrrolidine-2-carboxamide (Compound 3).**

**Step 1: Ethyl 6-(4-(3-amino-6-chloropyridazin-4-yl)piperazin-1-yl)nicotinate.** To a solution of 1-(5-ethoxycarbonyl-pyridin-2-yl)piperazine (2.19 g, 9.30 mmol) in DMSO (3 mL) was added 3-amino-4-bromo-6-chloropyridazine (2.33 g, 11.2 mmol), followed by DIPEA (4.87 mL, 27.9 mmol) dropwise. The reaction mixture was stirred at 100 ^o^C for 18 h. The reaction mixture was diluted with water and the resulting precipitate was collected by filtration, washed with water and EtOAc and dried to afford ethyl 6-(4-(3-amino-6-chloropyridazin-4-yl)piperazin-1-yl)nicotinate (2.23 g, 6.14 mmol, 66% yield) as tan solid. ^1^H NMR (400 MHz, DMSO-*d*_6_) δ ppm 8.67 (d, *J*=2.28 Hz, 1 H), 7.97 (dd, *J*=9.12, 2.28 Hz, 1 H), 6.95 (d, *J*=8.17 Hz, 1 H), 6.94 (s, 1 H), 6.27 (s, 2 H), 4.26 (q, *J*=7.05 Hz, 2 H), 3.80 - 3.90 (m, 4 H), 3.03 - 3.14 (m, 4 H), 1.29 (t, *J*=7.15 Hz, 3 H). *m/z* (ESI, +ve ion): 363.0 (M+H)^+^.

**Step 2: 6-(4-(3-Amino-6-chloropyridazin-4-yl)piperazin-1-yl)nicotinic acid.** A suspension of ethyl 6-(4-(3-amino-6-chloropyridazin-4-yl)piperazin-1-yl)nicotinate (1.23 g, 3.38 mmol) in 25% aqueous hydrochloric acid (4.1 mL, 33.8 mmol) was stirred at 80 ^o^C for 18 h. The reaction mixture was neutralized with 5 N aqueous NaOH to pH 6-7. The resulting precipitate was collected by filtration, dried in a vacuum oven to give 6-(4-(3-amino-6-chloropyridazin-4-yl)piperazin-1-yl)nicotinic acid (0.62 g, 1.85 mmol, 55% yield) as tan solid. The filtrate was extracted with EtOAc (product stayed in aqueous layer). The aqueous solution was lyophilized to provide another 0.54 g of product as a light-tan solid (combined yield 100%). ^1^H NMR (400 MHz, DMSO-*d*_6_) δ ppm 8.62 (d, *J*=1.87 Hz, 1 H), 7.97 (dd, *J*=8.71, 2.07 Hz, 1 H), 6.95 (s, 1 H), 6.78 (d, *J*=8.91 Hz, 1 H), 6.23 (s, 2 H), 3.66 - 3.77 (m, 4 H), 3.01 - 3.14 (m, 4 H). *m/z* (ESI, +ve ion): 335.0 (M+H)^+^.

**Step 3: 6-(4-(3-Amino-6-chloropyridazin-4-yl)piperazin-1-yl)-*N*-((*S*)-1-((2*S*,4*R*)-4-hydroxy-2-(((*S*)-1-(4-(4-methylthiazol-5-yl)phenyl)ethyl)carbamoyl)pyrrolidin-1-yl)-3,3-dimethyl-1-oxobutan-2-yl)nicotinamide.** A mixture of 6-(4-(3-amino-6-chloropyridazin-4-yl)piperazin-1-yl)nicotinic acid (0.10 g, 0.30 mmol), TEA (0.13 mL, 0.90 mmol), (2*S*,4*R*)-1-((*S*)-2-amino-3,3-dimethylbutanoyl)-4-hydroxy-*N*-((*S*)-1-(4-(4-methylthiazol-5-yl)phenyl)ethyl)pyrrolidine-2-carboxamide (**Intermediate 1**, 0.33 g, 0.75 mmol), and bromotri(1-pyrrolidinyl)phosphonium hexafluorophosphate (0.15 g, 0.33 mmol) in DCM (1 mL) was stirred at r.t. for 1 h. The reaction mixture concentrated under reduced pressure (rotary evaporator) and purified by column chromatography on silica gel eluting with a gradient of 0-100% EtOAc/EtOH (3:1) in heptane affording 2-(4-(3-amino6-(4-(3-amino-6-chloropyridazin-4-yl)piperazin-1-yl)-*N*-((*S*)-1-((2*S*,4*R*)-4-hydroxy-2-(((*S*)-1-(4-(4-methylthiazol-5-yl)phenyl)ethyl)carbamoyl)pyrrolidin-1-yl)-3,3-dimethyl-1-oxobutan-2-yl)nicotinamide (67 mg, 0.088 mmol, 30% yield) as off-white solid*. m/z* (ESI, +ve ion): 761.1 (M+H)^+^.

**Step 4: 6-(4-(3-Amino-6-(2-hydroxyphenyl)pyridazin-4-yl)piperazin-1-yl)-*N*-((*S*)-1-((2*S*,4*R*)-4-hydroxy-2-(((*S*)-1-(4-(4-methylthiazol-5-yl)phenyl)ethyl)carbamoyl)pyrrolidin-1-yl)-3,3-dimethyl-1-oxobutan-2-yl)nicotinamide (Compound 3).** A mixture of 6-(4-(3-amino-6-chloropyridazin-4-yl)piperazin-1-yl)-*N*-((*S*)-1-((2*S*,4*R*)-4-hydroxy-2-(((*S*)-1-(4-(4-methylthiazol-5-yl)phenyl)ethyl)carbamoyl)pyrrolidin-1-yl)-3,3-dimethyl-1-oxobutan-2-yl)nicotinamide (71 mg, 0.093 mmol), 2-hydroxybenzeneboronic acid (26 mg, 0.19 mmol), X-Phos Pd G3 (7.9 mg, 9.3 µmol) and anhydrous potassium carbonate (32 mg, 0.23 mmol) were flushed with nitrogen and treated with 1,4-dioxane (0.35 mL) and water (0.12 mL). The reaction mixture was then heated at 100 ^o^C for 20 h. Additional boronic acid (12 mg), X-Phos Pd G3 (4 mg) and dioxane (0.3 mL) were added and the reaction mixture stirred at 100 ^o^C for another 6 h. The reaction mixture was partitioned between EtOAc and brine and the aqueous layer was back-extracted with EtOAc (3x) and the combined organics was dried (Na_2_SO_4_) and concentrated under reduced pressure (rotary evaporator). The crude material was purified by column chromatography on silica gel eluting with a gradient of 0-80% EtOAc/EtOH (3:1) in heptane, to provide 6-(4-(3-amino-6-(2-hydroxyphenyl)pyridazin-4-yl)piperazin-1-yl)-*N*-((*S*)-1-((2*S*,4*R*)-4-hydroxy-2-(((*S*)-1-(4-(4-methylthiazol-5-yl)phenyl)ethyl)carbamoyl)pyrrolidin-1-yl)-3,3-dimethyl-1-oxobutan-2-yl)nicotinamide (**Compound 3**, 17 mg, 0.020 mmol, 22% yield) as tan solid. ^1^H NMR (400 MHz, *CDCl_3_*) δ ppm 8.62-8.70 (m, 2H), 7.93 (dd, *J*=2.49, 8.91 Hz, 1H), 7.59 (dd, *J*=1.24, 8.09 Hz, 1H), 7.34-7.46 (m, 6H), 7.28-7.33 (m, 1H), 7.06 (dd, *J*=0.93, 8.19 Hz, 1H), 6.88-6.96 (m, 1H), 6.67 (dd, *J*=8.81, 18.14 Hz, 2H), 5.10 (t, *J*=7.15 Hz, 1H), 4.87 (s, 2H), 4.70-4.81 (m, 2H), 4.56 (br s, 1H), 4.22 (br d, *J*=11.40 Hz, 1H), 3.80-3.92 (m, 4H), 3.65 (dd, *J*=3.63, 11.51 Hz, 1H), 3.21-3.30 (m, 4H), 2.55-2.64 (m, 1H), 2.53 (s, 3H), 2.09 (br dd, *J*=7.98, 13.99 Hz, 1H), 1.49 (d, *J*=6.84 Hz, 3H), 1.13 (s, 9H). *m/z* (ESI, +ve ion): 819.3 (M+H)^+^.

**(2*S*,4*R*)-1-((*S*)-2-(3-(4-(3-Amino-6-(2-hydroxyphenyl)pyridazin-4-yl)piperazin-1-yl)benzamido)-3,3-dimethylbutanoyl)-4-hydroxy-*N*-((*S*)-1-(4-(4-methylthiazol-5-yl)phenyl)ethyl)pyrrolidine-2-carboxamide (Compound 4).**

**Step 1: Methyl 3-(4-(3-amino-6-chloropyridazin-4-yl)piperazin-1-yl)benzoate.** To a solution of methyl 3-(piperazin-1-yl)benzoate hydrochloride (2.04 g, 7.95 mmol, Zerenex) in DMSO (27 mL) was added 3-amino-4-bromo-6-chloropyridazine (1.99 g, 9.54 mmol, CombiBlocks Inc.), followed by DIPEA (5.6 mL, 31.8 mmol) dropwise. The reaction mixture was stirred at 100 ^o^C for 18 h. After cooling to r.t., the reaction mixture was diluted with water and extracted with EtOAc (100 mL). The aqueous layer was separated and back-extracted with EtOAc (3x). The combined organic layers was dried over Na_2_SO_4_ and concentrated under reduced pressure (rotary evaporator) and the crude product was purified by column chromatography on silica gel using a gradient of 0-60% EtOAc/EtOH (3:1) in heptane to afford methyl 3-(4-(3-amino-6-chloropyridazin-4-yl)piperazin-1-yl)benzoate (1.40 g, 4.00 mmol, 51% yield) as yellow solid. ^1^H NMR (400 MHz, *CDCl_3_*) δ ppm 7.64 (s, 1 H), 7.59 (d, *J*=7.67 Hz, 1 H), 7.36 (t, *J*=7.98 Hz, 1 H), 7.16 (dd, *J*=8.19, 1.97 Hz, 1 H), 6.81 (s, 1 H), 4.82 (br s, 2 H), 3.92 (s, 3 H), 3.36 - 3.43 (m, 4 H), 3.21 - 3.28 (m, 4 H). *m/z* (ESI, +ve ion): 348.2 (M+H)^+^.

**Step 2: 3-(4-(3-Amino-6-chloropyridazin-4-yl)piperazin-1-yl)benzoic acid.** A suspension of methyl 3-(4-(3-amino-6-chloropyridazin-4-yl)piperazin-1-yl)benzoate (0.70 g, 2.00 mmol) in 25% hydrochloric acid (2.4 mL, 20 mmol) was stirred at 80 ^o^C for 18 h. After cooling to r.t., the reaction mixture was neutralized with 5 N NaOH to pH 6-7. The resulting precipitate was collected by filtration, washed with water, dried in a vacuum oven at 60 ^o^C to afford 3-(4-(3-amino-6-chloropyridazin-4-yl)piperazin-1-yl)benzoic acid (0.68 g, 2.00 mmol, 100% yield) as tan solid. ^1^H NMR (400 MHz, DMSO-*d*_6_) δ ppm 7.51 (br s, 2 H), 7.33 - 7.44 (m, 2 H), 7.27 (br d, *J*=7.05 Hz, 1 H), 7.20 (s, 1 H), 3.36 - 3.44 (m, 4 H), 3.24 (br s, 4 H). *m/z* (ESI, +ve ion): 334.1 (M+H)^+^.

**Step 3: (2*S*,4*R*)-1-((*S*)-2-(3-(4-(3-Amino-6-chloropyridazin-4-yl)piperazin-1-yl)benzamido)-3,3-dimethylbutanoyl)-4-hydroxy-*N*-((*S*)-1-(4-(4-methylthiazol-5-yl)phenyl)ethyl)pyrrolidine-2-carboxamide.** A mixture of 3-(4-(3-amino-6-chloropyridazin-4-yl)piperazin-1-yl)benzoic acid (0.20 g, 0.60 mmol), (2*S*,4*R*)-1-((*S*)-2-amino-3,3-dimethylbutanoyl)-4-hydroxy-*N*-((*S*)-1-(4-(4-methylthiazol-5-yl)phenyl)ethyl)pyrrolidine-2-carboxamide (**Intermediate 1**, 0.67 g, 1.50 mmol), triethylamine (0.25 mL, 1.8 mmol), and bromotri(1-pyrrolidinyl)phosphonium hexafluorophosphate (0.31 g, 0.66 mmol, Sigma-Aldrich Corporation) in DCM (4 mL) was stirred at r.t. for 16 h. The reaction mixture was warmed to 40 ^o^C and stirred for another 36 h. After cooling to r.t., the reaction mixture was diluted with brine and extracted with EtOAc (3x). The organic layers were combined, dried (MgSO_4_) and concentrated under reduced pressure (rotary evaporator). The crude residue was purified by column chromatography on silica gel, eluting with a gradient of 0-90% EtOAc/EtOH (3:1) in heptane, to provide 2-(4-(3-amino-(2*S*,4*R*)-1-((*S*)-2-(3-(4-(3-amino-6-chloropyridazin-4-yl)piperazin-1-yl)benzamido)-3,3-dimethylbutanoyl)-4-hydroxy-*N*-((*S*)-1-(4-(4-methylthiazol-5-yl)phenyl)ethyl)pyrrolidine-2-carboxamide (0.14 g, 0.19 mmol, 32% yield) as off-white solid*.* ^1^H NMR (400 MHz, *CDCl_3_*) δ ppm 8.67 (s, 1 H), 7.30 - 7.45 (m, 8 H), 7.19 (d, *J*=7.46 Hz, 1 H), 7.10 (dd, *J*=8.29, 2.07 Hz, 1 H), 6.77 - 6.83 (m, 1 H), 5.09 (quin, *J*=7.15 Hz, 1 H), 4.87 (br s, 2 H), 4.71 - 4.79 (m, 2 H), 4.57 (br s, 1 H), 4.19 (br d, *J*=11.40 Hz, 1 H), 3.67 (dd, *J*=11.30, 3.63 Hz, 1 H), 3.19 - 3.25 (m, 8 H), 2.52 (s, 3 H), 1.49 (d, *J*=7.05 Hz, 3 H), 1.39 (t, *J*=7.36 Hz, 2 H), 1.13 (s, 9 H). *m/z* (ESI, +ve ion): 760.3 (M+H)^+^.

**Step 4: (2*S*,4*R*)-1-((*S*)-2-(3-(4-(3-Amino-6-(2-hydroxyphenyl)pyridazin-4-yl)piperazin-1-yl)benzamido)-3,3-dimethylbutanoyl)-4-hydroxy-*N*-((*S*)-1-(4-(4-methylthiazol-5-yl)phenyl)ethyl)pyrrolidine-2-carboxamide (Compound 4).** A mixture of (2*S*,4*R*)-1-((*S*)-2-(3-(4-(3-amino-6-chloropyridazin-4-yl)piperazin-1-yl)benzamido)-3,3-dimethylbutanoyl)-4-hydroxy-*N*-((*S*)-1-(4-(4-methylthiazol-5-yl)phenyl)ethyl)pyrrolidine-2-carboxamide (0.14 g, 0.19 mmol), 2-hydroxybenzeneboronic acid (52 mg, 0.38 mmol, Oakwood Products), X-Phos Pd G3 (16 mg, 0.019 mmol, Sigma-Aldrich Corporation) and potassium carbonate (65 mg, 0.47 mmol) were flushed with nitrogen and treated with 1,4-dioxane (0.71 mL) and water (0.24 mL). The reaction mixture was heated at 100 ^o^C for 20 h. After cooling to r.t., the reaction mixture was partitioned between EtOAc and brine. The aqueous layer was back-extracted with EtOAc (3x) and the combined organic layers was dried (MgSO_4_) and concentrated under reduced pressure (rotary evaporator). The crude residue was purified by column chromatography on silica gel eluting with a gradient of 0-100% EtOAc/EtOH (3:1) in heptane, to provide (2*S*,4*R*)-1-((*S*)-2-(3-(4-(3-amino-6-(2-hydroxyphenyl)pyridazin-4-yl)piperazin-1-yl)benzamido)-3,3-dimethylbutanoyl)-4-hydroxy-*N*-((*S*)-1-(4-(4-methylthiazol-5-yl)phenyl)ethyl)pyrrolidine-2-carboxamide (**Compound 4**, 37 mg, 0.045 mmol, 24% yield) as off-white solid. ^1^H NMR (400 MHz, *CDCl_3_*) δ ppm 8.61 (s, 1 H), 8.10 (s, 1 H), 7.41 - 7.54 (m, 2 H), 7.32 - 7.38 (m, 6 H), 7.24 - 7.32 (m, 3 H), 6.82 (br d, *J*=8.09 Hz, 1 H), 5.28 - 5.38 (m, 1 H), 4.64 (t, *J*=8.60 Hz, 1 H), 4.58 (dd, *J*=8.81, 4.66 Hz, 1 H), 4.46 (d, *J*=8.71 Hz, 1 H), 4.35 - 4.43 (m, 3 H), 4.02 - 4.10 (m, 2 H), 4.01- 4.04 (m, 1H), 3.60 - 3.72 (m, 3 H), 3.56 (dd, *J*=15.45, 9.23 Hz, 1 H), 3.20 - 3.33 (m, 2 H), 3.11 - 3.20 (m, 1 H), 2.47 (s, 3 H), 1.63 (s, 3 H), 1.18 - 1.23 (m, 2 H), 0.98 (s, 9 H). *m/z* (ESI, +ve ion): 818.3 (M+H)^+^.

**2-(4-(3-Amino-6-(2-hydroxyphenyl)pyridazin-4-yl)piperazin-1-yl)-*N*-((*S*)-1-((2*S*,4*R*)-4-hydroxy-2-(((*S*)-1-(4-(4-methylthiazol-5-yl)phenyl)ethyl)carbamoyl)pyrrolidin-1-yl)-3,3-dimethyl-1-oxobutan-2-yl)thiazole-4-carboxamide (Compound 5).**

**Step 1: Ethyl 2-(4-(*tert*-butoxycarbonyl)piperazin-1-yl)thiazole-4-carboxylate.** To a solution of 1-(*tert*-butoxycarbonyl)-piperazine (1.41 g, 7.57 mmol) and ethyl 2-bromothiazole-4-carboxylate (2.14 g, 9.08 mmol) in DMSO (5 mL) was added DIPEA (2.6 mL, 15.1 mmol). The resulting mixture was stirred at 100 ^o^C for 16 h, cooled to r.t. then the reaction mixture was diluted with DCM and washed with water. The organic layer was concentrated under reduced pressure (rotary evaporator) and purified by column chromatography on silica gel, eluting with a gradient of 0-40% EtOAc/heptane to afford ethyl 2-(4-(*tert*-butoxycarbonyl)piperazin-1-yl)thiazole-4-carboxylate (2.07 g, 80% yield) as yellow solid. ^1^H NMR (400 MHz, *CDCl_3_*) δ ppm 7.43 - 7.49 (m, 1 H), 4.32 - 4.40 (m, 2 H), 3.48 - 3.60 (m, 8 H), 1.48 (s, 9 H), 1.34 - 1.41 (m, 3 H). *m/z* (ESI, +ve ion): 342.0 (M+H)^+^.

**Step 2: Ethyl 2-(4-(3-amino-6-chloropyridazin-4-yl)piperazin-1-yl)thiazole-4-carboxylate.** Ethyl 2-(4-(*tert*-butoxycarbonyl)piperazin-1-yl)thiazole-4-carboxylate (2.07 g, 6.06 mmol) in DCM (10 mL) was treated with TFA (5.0 mL, 67.1 mmol) and stirred at r.t. for 2 h. The reaction mixture was concentrated to dryness under reduced pressure (rotary evaporator), then dissolved in DMSO (10 mL) and treated with DIPEA (5.29 mL, 30.3 mmol) at 0 °C, followed by 3-amino-4-bromo-6-chloropyridazine (1.52 g, 7.28 mmol). The resulting mixture was heated at 110 °C for 16 h, cooled to r.t., then the reaction mixture was diluted with DCM and washed with water and the organic layer was dried over MgSO_4_, filtered and concentrated under reduced pressure (rotary evaporator). The crude residue was purified by column chromatography on silica gel, eluting with a gradient of 0-10% MeOH in DCM to afford ethyl 2-(4-(3-amino-6-chloropyridazin-4-yl)piperazin-1-yl)thiazole-4-carboxylate (1.08 g, 48% yield) as orange solid. *m/z* (ESI, +ve ion): 369.0 (M+H)^+^.

**Step 3: 2-(4-(3-Amino-6-chloropyridazin-4-yl)piperazin-1-yl)thiazole-4-carboxylic acid.** To a stirred suspension of ethyl 2-(4-(3-amino-6-chloropyridazin-4-yl)piperazin-1-yl)thiazole-4-carboxylate (0.56 g, 1.52 mmol) in EtOH (15 mL) was added 5 N sodium hydroxide solution (2 mL, 107 mmol) in water (2 mL). The resulting mixture was stirred at 70 °C for 1 h then the reaction mixture was concentrated under reduced pressure (rotary evaporator), the residue was dissolved in water and neutralized with aqueous HCl. The precipitate was collected by filtration and washed with heptanes to afford 2-(4-(3-amino-6-chloropyridazin-4-yl)piperazin-1-yl)thiazole-4-carboxylic acid (0.47 g, 91% yield) as yellow solid. ^1^H NMR (400 MHz, DMSO-*d*_6_) δ ppm 7.59 - 7.76 (m, 1 H), 6.97 (s, 1 H), 6.26 (s, 2 H), 3.58 - 3.68 (m, 4 H), 3.08 - 3.16 (m, 4 H). *m/z* (ESI, +ve ion): 341 (M+H)^+^.

**Step 4: 2-(4-(3-Amino-6-chloropyridazin-4-yl)piperazin-1-yl)-*N*-((*S*)-1-((2*S*,4*R*)-4-hydroxy-2-(((*S*)-1-(4-(4-methylthiazol-5-yl)phenyl)ethyl)carbamoyl)pyrrolidin-1-yl)-3,3-dimethyl-1-oxobutan-2-yl)thiazole-4-carboxamide.** A mixture of 2-(4-(3-amino-6-chloropyridazin-4-yl)piperazin-1-yl)thiazole-4-carboxylic acid (40 mg, 0.12 mmol), (2*S*,4*R*)-1-((*R*)-2-amino-3,3-dimethylbutanoyl)-4-hydroxy-*N*-((*S*)-1-(4-(4-methylthiazol-5-yl)phenyl)ethyl)pyrrolidine-2-carboxamide hydrochloride (**Intermediate 1**, 57 mg, 0.12 mmol), bromotripyrrolidinophosphonium hexafluorophosphate (0.11 g, 0.24 mmol), and Et_3_N (62 μL, 0.44 mmol) in DCM (2 mL) was stirred at r.t. for 2 h. The reaction mixture was purified by column chromatography on silica gel, eluting with a gradient of 0-10% MeOH in DCM to afford 2-(4-(3-amino-6-chloropyridazin-4-yl)piperazin-1-yl)-*N*-((*S*)-1-((2*S*,4*R*)-4-hydroxy-2-(((*S*)-1-(4-(4-methylthiazol-5-yl)phenyl)ethyl)carbamoyl)pyrrolidin-1-yl)-3,3-dimethyl-1-oxobutan-2-yl)thiazole-4-carboxamide (65 mg, 72% yield) as yellow solid. ^1^H NMR (400 MHz, *CDCl_3_*) δ ppm 8.59 (s, 1 H), 7.73 (d, *J*=8.9 Hz, 1 H), 7.43 (d, *J*=7.7 Hz, 1 H), 7.28 - 7.36 (m, 4 H), 6.71 (s, 1 H), 5.01 (t, *J*=7.3 Hz, 1 H), 4.94 (s, 2 H), 4.68 (t, *J*=7.8 Hz, 1 H), 4.60 (d, *J*=8.9 Hz, 1 H), 4.47 (br s, 1 H), 4.08 (br d, *J*=11.4 Hz, 1 H), 3.57 - 3.60 (m, 4 H), 3.33 - 3.38 (m, 1 H), 3.12 (br s, 4 H), 2.30 - 2.62 (m, 5 H), 1.41 (d, *J*=7.0 Hz, 3 H), 1.04 (s, 9 H). *m/z* (ESI, +ve ion): 767.0 (M+H)^+^.

**Step 5: 2-(4-(3-Amino-6-(2-hydroxyphenyl)pyridazin-4-yl)piperazin-1-yl)-*N*-((*S*)-1-((2*S*,4*R*)-4-hydroxy-2-(((*S*)-1-(4-(4-methylthiazol-5-yl)phenyl)ethyl)carbamoyl)pyrrolidin-1-yl)-3,3-dimethyl-1-oxobutan-2-yl)thiazole-4-carboxamide (Compound 5).** 2-(4-(3-Amino-6-chloropyridazin-4-yl)piperazin-1-yl)-*N*-((*S*)-1-((2*S*,4*R*)-4-hydroxy-2-(((*S*)-1-(4-(4-methylthiazol-5-yl)phenyl)ethyl)carbamoyl)pyrrolidin-1-yl)-3,3-dimethyl-1-oxobutan-2-yl)thiazole-4-carboxamide (65 mg, 0.085 mmol), 2-hydroxyphenylboronic acid (35 mg, 0.25 mmol), X-Phos Pd G3 (7.2 mg, 8.47 µmol) and sodium carbonate (22 mg, 0.21 mmol) were flushed with nitrogen and treated with 1,4-dioxane (2 mL) and water (1 mL). The reaction mixture was heated at 100 ^o^C for 2 h and concentrated under reduced pressure (rotary evaporator). The crude residue was purified by column chromatography on silica gel, eluting with a gradient of 0-10% MeOH in DCM to afford **Compound 5** (22 mg, 32% yield) as off-white solid. ^1^H NMR (400 MHz, *CDCl_3_*) δ ppm 8.65 (s, 1 H), 7.80 (br d, *J*=8.9 Hz, 1 H), 7.58 (br d, *J*=7.5 Hz, 1 H), 7.46 - 7.52 (m, 1 H), 7.42 - 7.45 (m, 1 H), 7.26 - 7.40 (m, 7 H), 6.91 (br t, *J*=7.5 Hz, 1 H), 5.00 - 5.16 (m, 1 H), 4.87 - 5.00 (m, 1 H), 4.76 (br t, *J*=7.8 Hz, 1 H), 4.66 (br d, *J*=8.7 Hz, 1 H), 4.54 (br s, 1 H), 4.21 (br d, *J*=11.0 Hz, 1 H), 3.57 - 3.79 (m, 5 H), 3.12 - 3.35 (m, 4 H), 2.45 - 2.58 (m, 4 H), 2.01 - 2.12 (m, 1 H), 1.45 - 1.52 (m, 4 H), 1.06 - 1.18 (m, 9 H). *m/z* (ESI, +ve ion): 825.2 (M+H)^+^.

**(2*S*,4*R*)-1-((*S*)-2-(2-(4-(3-Amino-6-(2-hydroxyphenyl)pyridazin-4-yl)piperazin-1-yl)acetamido)-3,3-dimethylbutanoyl)-4-hydroxy-*N*-((*S*)-1-(4-(4-methylthiazol-5-yl)phenyl)ethyl)pyrrolidine-2-carboxamide (Compound 6, AU-15330)**

**Compound** **6 (AU-15330)** was synthesized according to the procedure provided in the patent WO 2019/207538 (Example 43).

**Step 1: *tert*-Butyl 2-(4-(3-amino-6-chloropyridazin-4-yl)piperazin-1-yl)acetate.** To a solution of (piperazin-1-yl)acetic acid *tert*-butyl ester (0.49 g, 2.47 mmol, CombiBlocks Inc.) in DMSO (8.2 mL) was added 3-amino-4-bromo-6-chloropyridazine (0.66 g, 3.16 mmol, CombiBlocks Inc.), followed by DIPEA (1.85 mL, 10.61 mmol) dropwise. The reaction mixture was stirred at 100 ^o^C for 18 h. The reaction mixture was diluted with water and extracted with EtOAc (3x). The organic layers were combined, dried (MgSO_4_), filtered and concentrated. The crude material was purified by column chromatography on silica gel eluting with a gradient of 0-70% 3:1 EtOAc/EtOH in heptane to provide *tert*-butyl 2-(4-(3-amino-6-chloropyridazin-4-yl)piperazin-1-yl)acetate (0.61 g, 1.85 mmol, 75% yield) as off-white solid. ^1^H NMR (400 MHz, *CDCl_3_*) δ ppm 6.74 (s, 1 H), 4.75 (s, 2 H), 3.22 (s, 2 H), 3.08 - 3.18 (m, 4 H), 2.80 (br d, *J*=4.81 Hz, 4 H), 1.49 (s, 9 H). *m/z* (ESI, +ve ion): 328.1 (M+H)^+^.

**Step 2: (2*S*,4*R*)-1-((*S*)-2-(2-(4-(3-Amino-6-chloropyridazin-4-yl)piperazin-1-yl)acetamido)-3,3-dimethylbutanoyl)-4-hydroxy-*N*-((*S*)-1-(4-(4-methylthiazol-5-yl)phenyl)ethyl)pyrrolidine-2-carboxamide.** To a solution of *tert*-butyl 2-(4-(3-amino-6-chloropyridazin-4-yl)piperazin-1-yl)acetate (0.60 g, 1.84 mmol) in DCM (6.1 mL) was added TFA (2.0 mL, 27.6 mmol) dropwise. The reaction mixture was stirred at r.t. for 16 h. The solvent was evaporated and to the residue was added DCM and concentrated again. To the residue in DCM (4 mL) was added (2*S*,4*R*)-1-((*S*)-2-amino-3,3-dimethylbutanoyl)-4-hydroxy-*N*-((*S*)-1-(4-(4-methylthiazol-5-yl)phenyl)ethyl)pyrrolidine-2-carboxamide (**Intermediate 1**, 1.23 g, 2.76 mmol), triethylamine (0.78 mL, 5.52 mmol) and then bromotripyrrolidinophosphonium hexafluorophosphate (0.94 g, 2.02 mmol, Sigma-Aldrich Corporation). The reaction mixture was stirred at r.t. for 16 h. LCMS indicated no reaction. The precipitate was collected and found to contain both starting materials. To this precipitate was added DMF (4 mL), HATU (0.70 g, 1.84 mmol) and DIPEA (0.64 mL, 3.68 mmol) and the reaction was stirred at r.t. for 16 h. The reaction mixture was diluted with water and extracted with EtOAc (3x). The organic layers were combined, dried over anhydrous magnesium sulfate, filtered and concentrated. The crude material was purified by column chromatography on silica gel eluting with a gradient of 0-100% 3:1 EtOAc/EtOH in heptane, then switched to 20% MeOH/DCM, to afford (2*S*,4*R*)-1-((S)-2-(2-(4-(3-amino-6-chloropyridazin-4-yl)piperazin-1-yl)acetamido)-3,3-dimethylbutanoyl)-4-hydroxy-*N*-((*S*)-1-(4-(4-methylthiazol-5-yl)phenyl)ethyl)pyrrolidine-2-carboxamide (0.27 g, 0.39 mmol, 21% yield) as off-white solid. ^1^H NMR (400 MHz, *CDCl_3_*) δ ppm 11.94 - 12.56 (m, 1 H), 10.64 - 11.19 (m, 1 H), 8.68 (s, 1 H), 7.31 - 7.51 (m, 4 H), 6.79 (s, 1 H), 5.09 (br t, *J*=7.11 Hz, 1 H), 4.66 - 4.90 (m, 3 H), 4.44 - 4.61 (m, 2 H), 4.17 (br d, *J*=11.29 Hz, 1 H), 3.55 - 3.79 (m, 3 H), 3.03 - 3.24 (m, 5 H), 2.67 - 2.84 (m, 4 H), 2.45 - 2.59 (m, 4 H), 2.00 - 2.20 (m, 1 H), 1.47 - 1.49 (d, *J*=6.90 Hz, 3 H), 1.08 (s, 9 H). *m/z* (ESI, +ve ion): 698.2 (M+H)^+^.

**Step 3: (2*S*,4*R*)-1-((*S*)-2-(2-(4-(3-Amino-6-(2-hydroxyphenyl)pyridazin-4-yl)piperazin-1-yl)acetamido)-3,3-dimethylbutanoyl)-4-hydroxy-*N*-((*S*)-1-(4-(4-methylthiazol-5-yl)phenyl)ethyl)pyrrolidine-2-carboxamide (Compound 6, AU-15330).** (2*S*,4*R*)-1-((*S*)-2-(2-(4-(3-Amino-6-chloropyridazin-4-yl)piperazin-1-yl)acetamido)-3,3-dimethylbutanoyl)-4-hydroxy-*N*-((*S*)-1-(4-(4-methylthiazol-5-yl)phenyl)ethyl)pyrrolidine-2-carboxamide (0.14 g, 0.19 mmol), (2-hydroxyphenyl)boronic acid (53 mg, 0.39 mmol, Oakwood), methanesulfonato(2-dicyclohexylphosphino-2',4',6'-tri-*i*-propyl-1,1'-biphenyl)(2'-amino-1,1'-biphenyl-2-yl)palladium(II), XPhos Pd G3 (16 mg, 0.019 mmol, Strem Chemicals, Inc.) and sodium carbonate (51 mg, 0.48 mmol) was evacuated under vacuum and then purged with nitrogen. 1,4-Dioxane (0.73 mL) and water (0.24 mL) were added and the reaction mixture was heated to 95 °C for 4 h. After cooling to r.t., MgSO_4_ was added to the reaction mixture. The reaction was then diluted with DCM, filtered and purified by column chromatography on silica gel using a gradient of 0-90% EtOAc in heptane to afford (2*S*,4*R*)-1-((*S*)-2-(2-(4-(3-amino-6-(2-hydroxyphenyl)pyridazin-4-yl)piperazin-1-yl)acetamido)-3,3-dimethylbutanoyl)-4-hydroxy-*N*-((*S*)-1-(4-(4-methylthiazol-5-yl)phenyl)ethyl)pyrrolidine-2-carboxamide (**Compound 6**, 77 mg, 0.10 mmol, 53% yield) as off-white solid. ^1^H NMR (400 MHz, *CDCl_3_*) δ ppm 8.67 (s, 1 H), 7.76 (br d, *J*=8.36 Hz, 1 H), 7.62 (dd, *J*=8.05, 1.36 Hz, 1 H), 7.28 - 7.45 (m, 5 H), 7.07 (dd, *J*=8.26, 0.94 Hz, 1 H), 6.88 - 6.97 (m, 1 H), 5.10 (t, *J*=7.32 Hz, 1 H), 4.79 (s, 2 H), 4.73 - 4.78 (m, 1 H), 4.55 (br s, 1 H), 4.51 (d, *J*=8.57 Hz, 1 H), 4.20 (br d, *J*=11.50 Hz, 1 H), 3.62 (dd, *J*=11.39, 3.45 Hz, 1 H), 3.50 (s, 1 H), 3.18 - 3.36 (m, 4 H), 3.17 (s, 2 H), 2.80 (br s, 4 H), 2.54 - 2.66 (m, 1 H), 2.53 (s, 3 H), 2.10 (br dd, *J*=13.59, 8.15 Hz, 1 H), 1.49 (d, *J*=6.90 Hz, 3 H), 1.10 (s, 9 H). *m/z* (ESI, +ve ion): 756.2 (M+H)^+^.

**(2*S*,4*R*)-1-((*S*)-2-(2-(2-(4-(3-Amino-6-(2-hydroxyphenyl)pyridazin-4-yl)piperazin-1-yl)pyridin-4-yl)acetamido)-3,3-dimethylbutanoyl)-4-hydroxy-*N*-((*S*)-1-(4-(4-methylthiazol-5-yl)phenyl)ethyl)pyrrolidine-2-carboxamide (Compound 7).**

**Step 1: Diethyl 2-(2-(4-(*tert*-butoxycarbonyl)piperazin-1-yl)pyridin-4-yl)malonate.** A solution of diethyl 2-(2-bromopyridin-4-yl)malonate (1.00 g, 3.16 mmol), *tert*-butyl piperazine-1-carboxylate (1.18 g, 6.33 mmol, Chempure) and DIPEA (1.66 mL, 9.49 mmol) in DMSO (10 mL) was stirred at 130 °C for 24 h. The reaction mixture was diluted with ice-cold water and extracted with EtOAc. The combined organic extracts were washed with brine, dried over Na_2_SO_4_, concentrated under reduced pressure (rotary evaporator) and purified by column chromatography on silica gel using 0-30 % EtOAc in hexane, to afford diethyl 2-(2-(4-(*tert*-butoxycarbonyl) piperazin-1-yl)pyridin-4-yl)malonate (0.33 g, 0.78 mmol, 25% yield) as light-yellow oil. ^1^H NMR (400 MHz, DMSO-*d*_6_) δ ppm 8.08 (d, *J*=5.1 Hz, 1 H), 6.78 (s, 1 H), 6.61 (d, *J*=5.2 Hz, 1 H), 5.22 (s, 1 H), 4.08 – 4.14 (m, 4 H), 3.83- 3.78 (m, 4 H), 3.44 – 3.38 (m, 4 H), 1.35 – 1.44 (s, 9 H), 1.17 (t, *J*=7.1 Hz, 6 H). *m/z* (ESI): 422.2 (M+H)^+^.

**Step 2: Diethyl 2-(2-(piperazin-1-yl)pyridin-4-yl)malonate.** To a solution of diethyl 2-(2-(4-(*tert*-butoxycarbonyl)piperazin-1-yl)pyridin-4-yl)malonate (0.33 g, 0.78 mmol) in DCM (10 mL) was added TFA (0.5 mL, 6.49 mmol) at 0 °C and the reaction mixture was stirred at r.t. for 12 h. The solvent was evaporated to afford diethyl 2-(2-(piperazin-1-yl)pyridin-4-yl)malonate (0.25 g, 0.78 mmol, 99% yield) as light yellow gummy oil. *m/z* (ESI): 322.1 (M+H)^+^.

**Step 3: Diethyl 2-(2-(4-(3-amino-6-chloropyridazin-4-yl)piperazin-1-yl)pyridin-4-yl)malonate.** A solution of diethyl 2-(2-(piperazin-1-yl)pyridin-4-yl)malonate (0.25 g, 0.78 mmol), 4-bromo-6-chloropyridazin-3-amine (0.16 g, 0.78 mmol) and DIPEA (0.41 mL, 2.33 mmol) in DMSO (5 mL) was stirred at 110 °C for 16 h. The reaction mixture was diluted with ice-cold water and extracted with EtOAc. The organic extract was dried over anhydrous Na_2_SO_4_, concentrated under reduced pressure (rotary evaporator) and purified by column chromatography on silica gel, eluting with 0-10% MeOH in DCM, to provide diethyl 2-(2-(4-(3-amino-6-chloropyridazin-4-yl)piperazin-1-yl)pyridin-4-yl)malonate (0.15 g, 0.33 mmol, 43% yield) as light brown gummy oil. ^1^H NMR (300 MHz, DMSO-*d*_6_) δ ppm 8.10 (dd, *J*=17.7, 5.1 Hz, 1 H), 6.95 (s, 1 H), 6.80 (s, 1 H), 6.56 – 6.72 (m, 1 H), 6.24 (s, 2 H), 3.97 – 4.24 (m, 3 H), 3.59 – 3.72 (m, 6 H), 3.09 (t, *J*=5.1 Hz, 4 H), 1.07 – 1.25 (m, 6 H). *m/z* (ESI): 449.1 (M+H)^+^.

**Step 4: 2-(2-(4-(3-Amino-6-chloropyridazin-4-yl)piperazin-1-yl)pyridin-4-yl)acetic acid.** To a solution of diethyl 2-(2-(4-(3-amino-6-chloropyridazin-4-yl)piperazin-1-yl)pyridin-4-yl)malonate (0.15 g, 0.33 mmol) in water (1 mL) was added K_2_CO_3_ (92 mg, 0.67 mmol) and the reaction mixture was stirred at 100 °C for 5 h. The pH was adjusted to pH 7 using 1.5 N HCl and the precipitated solids were collected by filtration and dried to afford 2-(2-(4-(3-amino-6-chloropyridazin-4-yl)piperazin-1-yl)pyridin-4-yl)acetic acid (0.080 g, 0.23 mmol, 69% yield) as off-white solid. ^1^H NMR (300 MHz, DMSO-*d*_6_) δ ppm 12.50 (s, 1 H), 8.05 (d, *J*=5.3 Hz, 1 H), 6.98 (s, 1 H), 6.88 (s, 1 H), 6.65 (d, *J*=5.3 Hz, 1 H), 6.38 (s, 2 H), 3.69 (s, 4 H), 3.56 (s, 2 H), 3.11 (s, 4 H). *m/z* (ESI): 349.1 (M+H)^+^.

**Step 5: (2*S*,4*R*)-1-((*S*)-2-(2-(2-(4-(3-Amino-6-chloropyridazin-4-yl)piperazin-1-yl)pyridin-4-yl)acetamido)-3,3-dimethylbutanoyl)-4-hydroxy-*N*-((*S*)-1-(4-(4-methylthiazol-5-yl)phenyl)ethyl)pyrrolidine-2-carboxamide.** To a solution of 2-(2-(4-(3-amino-6-chloropyridazin-4-yl)piperazin-1-yl)pyridin-4-yl)acetic acid (80 mg, 0.23 mmol), DIPEA (0.12 mL, 0.69 mmol) in DCM (5 mL) was added HATU (0.13 g, 0.34 mmol) at 0 °C followed by (2*S*,4*R*)-1-((*S*)-2-amino-3,3-dimethylbutanoyl)-4-hydroxy-*N*-((*R*)-1-(4-(4-methylthiazol-5-yl)phenyl)ethyl)pyrrolidine-2-carboxamide hydrochloride (**Intermediate 1**, 0.13 g, 0.28 mmol). The reaction mixture was stirred for at r.t. for 16 h then the reaction mixture was diluted with water and extracted with DCM. The organic extract was dried over anhydrous Na_2_SO_4_, concentrated under reduced pressure (rotary evaporator) and purified by column chromatography on silica gel eluting with 0-15% MeOH in DCM, to afford (2*S*,4*R*)-1-((*S*)-2-(2-(2-(4-(3-amino-6-chloropyridazin-4-yl)piperazin-1-yl)pyridin-4-yl)acetamido)-3,3-dimethylbutanoyl)-4-hydroxy-*N*-((*R*)-1-(4-(4-methylthiazol-5-yl)phenyl)ethyl)pyrrolidine-2-carboxamide (0.11 g, 0.14 mmol, 59% yield) as light yellow solid. *m/z* (ESI): 775.3 (M+H)^+^.

**Step 6: (2*S*,4*R*)-1-((*S*)-2-(2-(2-(4-(3-Amino-6-(2-hydroxyphenyl)pyridazin-4-yl)piperazin-1-yl)pyridin-4-yl)acetamido)-3,3-dimethylbutanoyl)-4-hydroxy-*N*-((*S*)-1-(4-(4-methylthiazol-5-yl)phenyl)ethyl)pyrrolidine-2-carboxamide (Compound 7).** A solution of (2*S*,4*R*)-1-((*S*)-2-(2-(2-(4-(3-amino-6-chloropyridazin-4-yl)piperazin-1-yl)pyridin-4-yl)acetamido)-3,3-dimethylbutanoyl)-4-hydroxy-*N*-((*R*)-1-(4-(4-methylthiazol-5-yl)phenyl)ethyl)pyrrolidine-2-carboxamide (0.10 g, 0.13 mmol), (2-hydroxyphenyl)boronic acid (21 mg, 0.16 mmol, Sigma Aldrich) and K_2_CO_3_ (36 mg, 0.26 mmol) in 1,4-dioxane (2 mL):water (0.4 mL) was degassed with nitrogen. Then X-Phos Pd G3 (5 mg, 6.45 μmol, Strem Chemicals) was added to the reaction mixture and stirred at 100 °C for 16 h. The reaction mixture was diluted with ice water and extracted with EtOAc and the organic extract was washed with brine, dried over anhydrous Na_2_SO_4_, concentrated under reduced pressure (rotary evaporator) and purified by column chromatography on silica gel using 0-10% MeOH in DCM, to provide (2*S*,4*R*)-1-((*S*)-2-(2-(2-(4-(3-amino-6-(2-hydroxyphenyl)pyridazin-4-yl)piperazin-1-yl)pyridin-4-yl)acetamido)-3,3-dimethylbutanoyl)-4-hydroxy-*N*-((*R*)-1-(4-(4-methylthiazol-5-yl)phenyl)ethyl)pyrrolidine-2-carboxamide (**Compound 7**, 44 mg, 0.053 mmol, 41% yield) as off-white solid. ^1^H NMR (400 MHz, DMSO-*d*_6_) δ ppm 14.22 (s, 1 H), 8.98 (s, 1 H), 8.38 (d, *J* = 7.8 Hz, 1 H), 8.16 (d, *J*=9.2 Hz, 1 H), 8.04 (d, *J* = 5.1 Hz, 1 H), 7.99 – 7.83 (m, 1 H), 7.57 (s, 1 H), 7.43 (d, *J* = 8.1 Hz, 2 H), 7.37 (d, *J* = 8.1 Hz, 2 H), 7.30 – 7.17 (m, 2 H), 6.96 – 6.84 (m, 2 H), 6.60 (d, *J* = 5.2 Hz, 1 H), 6.40 (s, 2 H), 5.10 (d, *J* = 3.5 Hz, 1 H), 4.91 (d, *J* = 7.3 Hz, 1 H), 4.51 (d, *J* = 9.1 Hz, 1 H), 4.43 (t, *J* = 8.1 Hz, 1 H), 4.27 (d, *J* = 5.9 Hz, 1 H), 3.79 – 3.66 (m, 4 H), 3.64 – 3.53 (m, 3 H), 3.43 (d, *J* =13.7 Hz, 1 H), 3.19 (t, *J* = 4.9 Hz, 3 H), 2.45 (s, 3 H), 2.02 (dd, *J* = 13.1, 8.1 Hz, 1 H), 1.79 (ddd, *J*= 13.1, 8.7, 4.5 Hz, 1 H), 1.37 (d, *J* = 7.0 Hz, 3 H), 0.93 (d, *J* = 8.9 Hz, 9 H). *m/z* (ESI): 833.3 (M+H)^+^.

**(2*S*,4*R*)-1-((*S*)-2-(3-(2-(4-(3-Amino-6-(2-hydroxyphenyl)pyridazin-4-yl)piperazin-1-yl)pyridin-4-yl)propanamido)-3,3-dimethylbutanoyl)-4-hydroxy-*N*-((*S*)-1-(4-(4-methylthiazol-5-yl)phenyl)ethyl)pyrrolidine-2-carboxamide (Compound 8).**

**Step 1: *tert*-Butyl (*E*)-3-(2-bromopyridin-4-yl)acrylate.** To a suspension of NaH (0.77 g, 32.0 mmol) in THF (50 mL) was added *tert*-butyl 2-(diethoxyphosphoryl) acetate (8.14 g, 32.3 mmol, CombiBlocks) at 0 °C. After 10 min, 2-bromoisonicotinaldehyde (5.00 g, 26.9 mmol, CombiBlocks) was added dropwise and stirred at r.t. for 2 h. The reaction mixture was quenched with ice water and extracted with EtOAc. The organic extract was washed with brine, dried over anhydrous Na_2_SO_4_, concentrated under reduced pressure (rotary evaporator) and purified by column chromatography on silica gel eluting with a gradient of 10-13% EtOAc in hexane, to provide *tert*-butyl (*E*)-3-(2-bromopyridin-4-yl)acrylate (5.80 g, 20.41 mmol, 76% yield) as white solid. ^1^H NMR (400 MHz, DMSO-*d_6_*) δ ppm 8.41 (d, *J*=5.1 Hz, 1 H), 7.98 – 8.02 (m, 1 H), 7.76 (dd, *J*=5.1, 1.5 Hz, 1 H), 7.50 (d, *J*=16.1 Hz, 1 H), 6.87 (d, *J*=16.1 Hz, 1 H), 1.49 (s, 9 H). *m/z* (ESI): 284.0 and 286.0 (M+H)^+^.

**Step 2: (*E*)-3-(2-(4**-(***tert***-**Butoxycarbonyl)piperazin-1-yl)pyridin-4-yl)acrylic acid.** A solution of *tert*-butyl (*E*)-3-(2-bromopyridin-4-yl)acrylate (1.00 g, 3.52 mmol), *tert*-butyl piperazine-1-carboxylate (0.79 g, 4.22 mmol, Chempure) and sodium *tert*-butoxide (0.68 g, 7.04 mmol) in 1,4-dioxane (10 mL) was degassed with nitrogen for 2 min. Then Xantphos (0.20 g, 0.35 mmol, Arbor Chemicals) and Pd_2_(dba)_3_ (0.32 g, 0.35 mmol, Chempure) were added to the reaction mixture and it was stirred at 100 °C for 16 h. The reaction mixture was diluted with water and extracted with EtOAc and the organic extract was dried over anhydrous Na_2_SO_4_, concentrated under reduced pressure (rotary evaporator) to provide (*E*)-3-(2-(4-(*tert*-butoxycarbonyl)piperazin-1-yl)pyridin-4-yl)acrylic acid as yellow solid (1.4 g). ^1^H NMR (400 MHz, DMSO-*d*_6_) δ ppm 8.10 (d, *J*=5.1 Hz, 1 H), 7.28 (d, *J*=15.9 Hz, 1 H), 7.02 (s, 1 H), 6.88 (d, *J*=5.2 Hz, 1 H), 6.69 (d, *J*=15.9 Hz, 1 H), 3.54 – 3.50 (m, 4 H), 3.44 -3.40 (m, 4 H), 1.32 – 1.52 (s, 9 H). *m/z* (ESI): 334.2 (M+H)^+^.

**Step 3: *tert*-Butyl (*E*)-4-(4-(3-methoxy-3-oxoprop-1-en-1-yl)pyridin-2-yl)piperazine-1-carboxylate.** To a solution of (*E*)-3-(2-(4-(*tert*-butoxycarbonyl)piperazin-1-yl)pyridin-4-yl)acrylic acid (1.00 g, 3.00 mmol) in DCM (10 mL) was added dropwise TMS-diazomethane in diethyl ether (6.00 mL, 12.00 mmol) at 0 °C and the reaction mixture was stirred at r.t. for 5 h. The reaction mixture was diluted with water and extracted with DCM. The organic extract was dried over anhydrous Na_2_SO_4_, concentrated under reduced pressure (rotary evaporator) and purified by column chromatography on silica gel eluting with a gradient of 20-25% EtOAc in petroleum ether to afford *tert*-butyl (*E*)-4-(4-(3-methoxy-3-oxoprop-1-en-1-yl)pyridin-2-yl)piperazine-1-carboxylate (0.50 g, 1.44 mmol, 48% yield) as orange oil. *m/z* (ESI): 348.2 (M+H)^+^.

**Step 4: *tert*-Butyl 4-(4-(3-methoxy-3-oxopropyl)pyridin-2-yl)piperazine-1-carboxylate.** A solution of *tert*-butyl (*E*)-4-(4-(3-methoxy-3-oxoprop-1-en-1-yl)pyridin-2-yl)piperazine-1-carboxylate (0.60 g, 1.73 mmol) and Pd/C (0.15 g, 0.14 mmol) in MeOH (10 mL) was stirred under an atmosphere of hydrogen (1 atm) for 4 h. The reaction mixture was filtered through a pad of celite and washed with MeOH. The reaction mixture was concentrated under reduced pressure (rotary evaporator) to afford *tert*-butyl 4-(4-(3-methoxy-3-oxopropyl)pyridin-2-yl)piperazine-1-carboxylate (0.50 g, 83% yield) as light-yellow oil which was taken to the subsequent step without purification. *m/z* (ESI): 350.2 (M+H).

**Step 5: Methyl 3-(2-(piperazin-1-yl)pyridin-4-yl)propanoate.** To a solution of *tert*-butyl 4-(4-(3-methoxy-3-oxopropyl)pyridin-2-yl)piperazine-1-carboxylate (0.53 g, 1.52 mmol) in 1,4-dioxane (2 mL) was added 4M HCl in dioxane (1 mL) at 0 °C and stirred at r.t. for 2 h. The reaction mixture was concentrated under reduced pressure (rotary evaporator) to afford methyl 3-(2-(piperazin-1-yl)pyridin-4-yl)propanoate hydrochloride (0.43 g, 1.51 mmol, 99% yield) as white solid which was taken to the subsequent step without further purification.

**Step 6: Methyl 3-(2-(4-(3-amino-6-chloropyridazin-4-yl)piperazin-1-yl)pyridin-4-yl)propanoate.** To a solution of 4-bromo-6-chloropyridazin-3-amine (0.33 g, 1.58 mmol, CombiBlocks) and methyl 3-(2-(piperazin-1-yl)pyridin-4-yl)propanoate hydrochloride (0.45 g, 1.58 mmol) in DMSO (3 mL) was added DIPEA (1.38 mL, 7.92 mmol) and stirred at 100 °C for 16 h. The reaction mixture was diluted with water and extracted with EtOAc and the organic extract was dried over anhydrous Na_2_SO_4_ and concentrated under reduced pressure (rotary evaporator) and purified by column chromatography on silica gel eluting with 10-11% MeOH in DCM, to provide methyl 3-(2-(4-(3-amino-6-chloropyridazin-4-yl)piperazin-1-yl)pyridin-4-yl)propanoate (0.15 g, 0.40 mmol, 27% yield) as light-yellow solid. ^1^H NMR (300 MHz, DMSO-*d_6_*) δ ppm 8.02 (d, *J*=5.1 Hz, 1 H), 6.95 (s, 1 H), 6.76 (s, 1 H), 6.57 (d, *J*=5.3 Hz, 1 H), 6.24 (s, 2 H), 3.69 - 3.65 (m, 4 H), 3.60 (s, 3 H), 2.78 (t, *J*=7.5 Hz, 2 H), 2.61 – 2.72 (m, 4 H), 2.56 (d, *J*=8.3 Hz, 2 H). *m/z* (ESI): 377.1 (M+H)^+^.

**Step 7: 3-(2-(4-(3-Amino-6-chloropyridazin-4-yl)piperazin-1-yl)pyridin-4-yl)propanoic acid.** To a solution of methyl 3-(2-(4-(3-amino-6-chloropyridazin-4-yl)piperazin-1-yl)pyridin-4-yl)propanoate (0.15 g, 0.40 mmol) in MeOH (1 mL), THF (1 mL) and water (0.5 mL) was added LiOH monohydrate (50 mg, 1.19 mmol). The reaction mixture was stirred at r.t. for 4 h. The reaction mixture was concentrated under reduced pressure (rotary evaporator), diluted with water and acidified using 1 N HCl (to pH 6). The precipitated solid was collected by filtration and dried to provide 3-(2-(4-(3-amino-6-chloropyridazin-4-yl)piperazin-1-yl)pyridin-4-yl)propanoic acid (0.12 g, 0.33 mmol, 83% yield) as white solid. ^1^H NMR (300 MHz, DMSO-*d_6_*) δ ppm 12.19 (s, 1 H), 8.02 (d, *J*=5.2 Hz, 1 H), 6.96 (s, 1 H), 6.80 (s, 1 H), 6.60 (d, *J*=5.2 Hz, 1 H), 6.28 (s, 2 H), 3.70 – 3.66 (s, 4 H), 3.08 (t, *J*=4.8 Hz, 4 H), 2.77 (d, *J*=7.6 Hz, 2 H), 2.58 (d, *J*=7.4 Hz, 2 H). *m/z* (ESI): 363.1 (M+H)^+^.

**Step 8: (2*S*,4*R*)-1-((*S*)-2-(3-(2-(4-(3-Amino-6-chloropyridazin-4-yl)piperazin-1-yl)pyridin-4-yl)propanamido)-3,3-dimethylbutanoyl)-4-hydroxy-*N*-((*S*)-1-(4-(4-methylthiazol-5-yl)phenyl)ethyl)pyrrolidine-2-carboxamide.** To a solution of 3-(2-(4-(3-amino-6-chloropyridazin-4-yl)piperazin-1-yl)pyridin-4-yl)propanoic acid (0.12 g, 0.33 mmol) in DCM (3 mL) were added HATU (0.19 g, 0.50 mmol) followed by DIPEA (0.23 mL, 1.32 mmol) at 0 °C, after 15 min was added (2*S*,4*R*)-1-((*S*)-2-amino-3,3-dimethylbutanoyl)-4-hydroxy-*N*-((*S*)-1-(4-(4-methylthiazol-5-yl)phenyl)ethyl)pyrrolidine-2-carboxamide hydrochloride (**Intermediate 1**, 0.16 g, 0.33 mmol). The reaction mixture was stirred at r.t. for 18 h then the reaction mixture was diluted with water and extracted with DCM. The organic extract was dried over anhydrous Na_2_SO_4_, concentrated under reduced pressure (rotary evaporator) and purified by column chromatography on silica gel eluting with 10-11% MeOH in DCM, to provide (2*S*,4*R*)-1-((*S*)-2-(3-(2-(4-(3-amino-6-chloropyridazin-4-yl)piperazin-1-yl)pyridin-4-yl)propanamido)-3,3-dimethylbutanoyl)-4-hydroxy-*N*-((*S*)-1-(4-(4-methylthiazol-5-yl)phenyl)ethyl)pyrrolidine-2-carboxamide (0.20 g, 0.25 mmol, 77% yield) as yellow solid. ^1^H NMR (300 MHz, DMSO-*d_6_*) δ ppm 8.99 (s, 1 H), 8.38 (d, *J*=7.8 Hz, 1 H), 8.00 (d, *J*=5.0 Hz, 1 H), 7.90 (d, *J*=9.3 Hz, 1 H), 7.44 (d, *J*=8.4 Hz, 2 H), 7.38 (d, *J*=8.2 Hz, 2 H), 6.95 (s, 1 H), 6.75 (s, 1 H), 6.56 (d, *J*=5.1 Hz, 1 H), 6.24 (s, 2 H), 5.12 (d, *J*=3.4 Hz, 1 H), 4.87 – 4.97 (m, 1 H), 4.53 (d, *J*=9.3 Hz, 1 H), 4.43 (t, *J*=8.1 Hz, 1 H), 4.29 (s, 1 H), 4.10 (q, *J*=5.3 Hz, 1 H), 3.64 (d, *J*=17.8 Hz, 6 H), 3.17 (d, *J*=5.2 Hz, 2 H), 3.09 – 3.05 (m, 4 H), 2.62 (dd, *J*=20.7, 13.2 Hz, 1 H), 2.46 (s, 3 H), 2.01 (d, *J*=9.3 Hz, 1 H), 1.37 (d, *J*=6.9 Hz, 3 H), 0.90 (s, 9 H). *m/z* (ESI): 790.3 (M+H)^+^.

**Step 9: (2*S*,4*R*)-1-((*S*)-2-(3-(2-(4-(3-Amino-6-(2-hydroxyphenyl)pyridazin-4-yl)piperazin-1-yl)pyridin-4-yl)propanamido)-3,3-dimethylbutanoyl)-4-hydroxy-*N*-((*S*)-1-(4-(4-methylthiazol-5-yl)phenyl)ethyl)pyrrolidine-2-carboxamide (Compound 8).** A solution of (2*S*,4*R*)-1-((*S*)-2-(3-(2-(4-(3-amino-6-chloropyridazin-4-yl)piperazin-1-yl)pyridin-4-yl)propanamido)-3,3-dimethylbutanoyl)-4-hydroxy-*N*-((*S*)-1-(4-(4-methylthiazol-5-yl)phenyl)ethyl)pyrrolidine-2-carboxamide (0.20 g, 0.25 mmol), (2-hydroxyphenyl)boronic acid (0.042 g, 0.30 mmol, CombiBlocks) and K_2_CO_3_ (70 mg, 0.51 mmol) in dioxane (5 mL) and water (1 mL) was degassed with nitrogen then treated with X-Phos Pd G-3 (9.8 mg, 0.013 mmol, Aldrich). The reaction mixture was stirred at 100 °C for 18 h, diluted with water and extracted with EtOAc. The organic extract was dried over anhydrous Na_2_SO_4_, concentrated under reduced pressure (rotary evaporator) and purified by column chromatography on silica gel eluting with a gradient of 10-11% MeOH in DCM to afford (2*S*,4*R*)-1-((*S*)-2-(3-(2-(4-(3-amino-6-(2-hydroxyphenyl)pyridazin-4-yl)piperazin-1-yl)pyridin-4-yl)propanamido)-3,3-dimethylbutanoyl)-4-hydroxy-*N*-((*S*)-1-(4-(4-methylthiazol-5-yl)phenyl)ethyl)pyrrolidine-2-carboxamide (**Compound 8**, 0.15 g, 0.18 mmol, 70% yield) as white solid. ^1^H NMR (400 MHz, DMSO-*d_6_*) δ ppm 14.22 (s, 1 H), 8.98 (s, 1 H), 8.37 (d, *J*=7.8 Hz, 1 H), 8.02 (d, *J*=5.1 Hz, 1 H), 7.92 (t, *J*=9.5 Hz, 2 H), 7.55 (d, *J*=5.2 Hz, 1 H), 7.31 – 7.48 (m, 4 H), 7.24 (t, *J*=7.6 Hz, 1 H), 6.81 – 6.96 (m, 2 H), 6.77 (s, 1 H), 6.57 (d, *J*=5.4 Hz, 1 H), 6.39 (s, 2 H), 5.12 (d, *J*=3.5 Hz, 1 H), 4.90 (d, *J*=7.3 Hz, 1 H), 4.53 (d, *J*=9.3Hz, 1 H), 4.42 (t, *J*=8.1 Hz, 1 H), 4.28 (s, 1 H), 3.71 (d, *J*=6.0 Hz, 4 H), 3.60 (s, 2 H), 3.18 (d, *J*=5.9 Hz, 4 H), 2.75(d, *J*=7.2 Hz, 1 H), 2.63 (d, *J*=7.5 Hz, 1 H), 2.45 (s, 3 H), 2.00 (d, *J*=9.6 Hz, 1 H), 1.77 (d, *J*=13.5 Hz, 1 H), 1.36 (d, *J*=6.9 Hz, 3 H), 0.89 (s, 9 H). *m/z* (ESI): 847.3 (M+H)^+^.

**6-(4-(6-Amino-3-(2-hydroxyphenyl)-1,2,4-triazin-5-yl)piperazin-1-yl)-*N*-((*S*)-1-((2*S*,4*R*)-4-hydroxy-2-(((*S*)-1-(4-(4-methylthiazol-5-yl)phenyl)ethyl)carbamoyl)pyrrolidin-1-yl)-3,3-dimethyl-1-oxobutan-2-yl)picolinamide (Compound 9).**

**Step 1: 2-Hydroxybenzimidohydrazide.** To a solution of hydrazine (54.6 mL, 54.6 mmol, Sigma Aldrich) in THF was added LiHMDS (1.0 M in THF, 126 mL, 126 mmol, Spectrochem) at -78 °C. After 30 min, 2-hydroxybenzonitrile (5.00 g, 42.0 mmol, CombiBlocks) in THF was added to the reaction mixture and stirred at r.t. for 12 h. The reaction mixture was quenched with water and extracted with EtOAc. The combined organic layers were dried over Na_2_SO_4_ and concentrated to afford crude 2-hydroxybenzimidohydrazide (5.00 g, 24.81 mmol, 59% yield) as colorless liquid. *m/z* (ESI): 152.1 (M+H)^+^.

**Step 2: 6-Amino-3-(2-hydroxyphenyl)-1,2,4-triazin-5-ol.** A mixture of ethyl 2-amino-2-thioxoacetate (4.29 g, 32.2 mmol, Sigma Aldrich) and 2-hydroxybenzimidohydrazide (5.00 g, 24.81 mmol) in EtOH was stirred at 100 °C for 12 h. The reaction mixture was concentrated, diluted with diethyl ether and stirred at 0 °C. The suspension was filtered and washed with cold ethanol to afford 6-amino-3-(2-hydroxyphenyl)-1,2,4-triazin-5-ol (2.00 g, 9.75 mmol, 39% yield) as pale yellow solid. ^1^H NMR (400 MHz, DMSO-*d*_6_) δ ppm 13.32 (br s, 2 H), 7.95 (d, *J*=8.0 Hz, 1 H), 7.38 (t, *J*=7.8 Hz, 1 H), 6.80 – 7.07 (m, 4 H). *m/z* (ESI): 205.1 (M+H)^+^.

**Step 3:** **2-(6-Amino-5-chloro-1,2,4-triazin-3-yl)phenol.** A mixture of 6-amino-3-(2-hydroxyphenyl)-1,2,4-triazin-5-ol (2.00 g, 9.79 mmol) and SOCl_2_ (20 mL, 274 mmol, Spectrochem) was stirred at 70 °C for 3 h. The reaction mixture was concentrated under reduced pressure (rotary evaporator) to afford 2-(6-amino-5-chloro-1,2,4-triazin-3-yl)phenol (2.10 g, 9.43 mmol, 96% yield) as pale yellow solid. ^1^H NMR (400 MHz, DMSO-*d*_6_) δ ppm 10.65 (br s, 1 H), 6.97 – 7.84 (m, 5 H), 6.92 (t, *J*=7.6 Hz, 1 H). *m/z* (ESI): 223.0 (M+H)^+^.

**Step 4: *tert*-Butyl 4-(6-amino-3-(2-hydroxyphenyl)-1,2,4-triazin-5-yl)piperazine-1-carboxylate.** A mixture of 2-(6-amino-5-chloro-1,2,4-triazin-3-yl)phenol (2.00 g, 8.98 mmol), *tert*-butyl piperazine-1-carboxylate (3.35 g, 17.97 mmol, Spectrochem), DIPEA (7.85 mL, 44.9 mmol, Spectrochem) in DMSO was stirred at 120 °C for 1 h in a microwave. The reaction mixture was diluted with water and extracted with EtOAc. The combined organic layers were dried over Na_2_SO_4_, filtered, concentrated under reduced pressure (rotary evaporator) and purified by column chromatography on silica gel eluting with a gradient of 40-80% EtOAc in petroleum ether to afford *tert*-butyl 4-(6-amino-3-(2-hydroxyphenyl)-1,2,4-triazin-5-yl)piperazine-1-carboxylate (0.95 g, 1.35 mmol, 15% yield) as brown solid. *m/z* (ESI): 373.1 (M+H)^+^.

**Step 5: 2-(6-Amino-5-(piperazin-1-yl)-1,2,4-triazin-3-yl)phenol.** A mixture of *tert*-butyl 4-(6-amino-3-(2-hydroxyphenyl)-1,2,4-triazin-5-yl)piperazine-1-carboxylate (0.95 g, 2.55 mmol) and HCl (4.0 M in dioxane, 0.64 mL, 2.55 mmol) in DCM was stirred at r.t. for 2 h. The reaction mixture was concentrated under reduced pressure (rotary evaporator) to afford 2-(6-amino-5-(piperazin-1-yl)-1,2,4-triazin-3-yl)phenol hydrochloride (0.87 g, 1.78 mmol, 70% yield) as brown solid. *m/z* (ESI): 273.1 (M+H)^+^.

**Step 6: Methyl 6-(4-(6-amino-3-(2-hydroxyphenyl)-1,2,4-triazin-5-yl)piperazin-1-yl)picolinate.** A mixture of 2-(6-amino-5-(piperazin-1-yl)-1,2,4-triazin-3-yl)phenol hydrochloride (0.85 g, 1.73 mmol), methyl 6-bromopicolinate (0.38 g, 1.73 mmol, CombiBlocks) and DIPEA (0.91 mL, 5.20 mmol) in DMSO was stirred at 120 °C for 48 h. The reaction mixture was cooled to r.t., diluted with cold water and extracted with EtOAc. The combined organic layers were dried over Na_2_SO_4_, filtered, concentrated under reduced pressure (rotary evaporator) and purified by column chromatography on silica gel eluting with a gradient of 5-15% EtOAc in petroleum ether to afford methyl 6-(4-(6-amino-3-(2-hydroxyphenyl)-1,2,4-triazin-5-yl)piperazin-1-yl)picolinate (0.12 g, 0.22 mmol, 13% yield) as pale yellow solid. *m/z* (ESI): 408.1 (M+H)^+^.

**Step 7: 6-(4-(6-Amino-3-(2-hydroxyphenyl)-1,2,4-triazin-5-yl)piperazin-1-yl)picolinic acid.** A mixture of methyl 6-(4-(6-amino-3-(2-hydroxyphenyl)-1,2,4-triazin-5-yl)piperazin-1-yl)picolinate (0.12 g, 0.21 mmol), and LiOH monohydrate (7.6 mg, 0.32 mmol, Spectrochem) in THF and water was stirred at r.t. for 3 h. The reaction mixture was concentrated, diluted with ice-water and the pH was adjusted to pH 5-6 using 1.5 N HCl. The resulting precipitate was collected by filtration and dried to afford 6-(4-(6-amino-3-(2-hydroxyphenyl)-1,2,4-triazin-5-yl)piperazin-1-yl)picolinic acid (80 mg, 0.17 mmol, 79% yield) as pale yellow solid. ^1^H NMR (400 MHz, DMSO-*d*_6_) δ ppm 13.39 (s, 1 H), 8.16 (d, *J*=8.0 Hz, 1 H), 7.73 (t, *J*=8.0 Hz, 1 H), 7.23 – 7.44 (m, 2 H), 7.12 (d, *J*=8.4 Hz, 1 H), 6.92 (t, *J*=8.7 Hz, 2 H), 6.61 (s, 2 H), 3.79 (br s, 8 H). *m/z* (ESI): 394.1 (M+H)^+^.

**Step 8: 6-(4-(6-Amino-3-(2-hydroxyphenyl)-1,2,4-triazin-5-yl)piperazin-1-yl)-*N*-((*S*)-1-((2*S*,4*R*)-4-hydroxy-2-(((*S*)-1-(4-(4-methylthiazol-5-yl)phenyl)ethyl)carbamoyl)pyrrolidin-1-yl)-3,3-dimethyl-1-oxobutan-2-yl)picolinamide (Compound 9).** To a mixture of 6-(4-(6-amino-3-(2-hydroxyphenyl)-1,2,4-triazin-5-yl)piperazin-1-yl)picolinic acid (60 mg, 0.15 mmol), HATU (87 mg, 0.23 mmol, Spectrochem), and DIPEA (53 µL, 0.31 mmol) in DMF was added (2*S*,4*R*)-1-((*S*)-2-amino-3,3-dimethylbutanoyl)-4-hydroxy-*N*-((*S*)-1-(4-(4-methylthiazol-5-yl)phenyl)ethyl)pyrrolidine-2-carboxamide hydrochloride (**Intermediate 1**, 73 mg, 0.15 mmol) and stirred at 80 °C for 48 h. The reaction mixture was diluted with cold water and extracted with EtOAc. The combined organic layer was dried over Na_2_SO_4_, filtered, concentrated under reduced pressure (rotary evaporator) and purified by preparative HPLC to afford 6-(4-(6-amino-3-(2-hydroxyphenyl)-1,2,4-triazin-5-yl)piperazin-1-yl)-*N*-((*S*)-1-((2*S*,4*R*)-4-hydroxy-2-(((*S*)-1-(4-(4-methylthiazol-5-yl)phenyl)ethyl)carbamoyl)pyrrolidin-1-yl)-3,3-dimethyl-1-oxobutan-2-yl)picolinamide (**Compound 9**, 20 mg, 0.024 mmol, 16% yield) as pale yellow solid. ^1^H NMR (400 MHz, DMSO-*d*_6_) δ ppm 13.39 (s, 1 H), 8.99 (s, 1 H), 8.42 – 8.53 (m, 2 H), 8.18 (dd, *J*=8.1, 1.8 Hz, 1 H), 7.79 (t, *J*=7.9 Hz, 1 H), 7.27 – 7.51 (m, 6 H), 7.19 (d, *J*=8.5 Hz, 1 H), 6.89 – 6.99 (m, 2 H), 6.65 (s, 2 H), 5.15 (d, *J*=3.5 Hz, 1 H), 4.86 – 4.96 (m, 1 H), 4.69 (d, *J*=9.8 Hz, 1 H), 4.49 (t, *J*=8.2 Hz, 1 H), 4.30 (br s, 1 H), 3.72 – 3.92 (br m, 8 H), 3.66 (br s, 2 H), 2.46 (s, 3 H), 2.05 – 2.16 (m, 1 H), 1.72 – 1.82 (m, 1 H), 1.40 (d, *J*=7.0 Hz, 3 H), 1.02 (s, 9 H). *m/z* (ESI): 820.3 (M+H)^+^.

***N*-((*S*)-1-((2*S*,4*R*)-4-Hydroxy-2-(((*S*)-1-(4-(4-methylthiazol-5-yl)phenyl)ethyl)carbamoyl)pyrrolidin-1-yl)-3,3-dimethyl-1-oxobutan-2-yl)-2-(4-(6-(2-hydroxyphenyl)pyridazin-4-yl)piperazin-1-yl)isonicotinamide (Compound 10).**

**Step 1: 2-(5-Chloropyridazin-3-yl)phenol.** A mixture of 3,5-dichloropyridazine (2.00 g, 13.43 mmol, CombiBlocks), (2-hydroxyphenyl)boronic acid (1.85 g, 13.43 mmol, CombiBlocks), cesium carbonate (10.94 g, 33.6 mmol, Spectrochem) and bis(diphenylphosphino)ferrocene (0.37 g, 0.67 mmol, Chempure) in 1,4-dioxane and water was treated with palladium(II) acetate (0.15 g, 0.67 mmol, Hindustan Platinum) and stirred at 70 °C for 16 h. The reaction mixture was cooled to r.t., filtered through a celite pad and the filtrate was diluted with water and extracted with EtOAc. The combined organic layers were washed with brine, dried over Na_2_SO_4_, filtered, concentrated under reduced pressure (rotary evaporator) and purified by column chromatography on silica gel using a gradient of 0-20% EtOAc in petroleum ether, to provide 2-(5-chloropyridazin-3-yl)phenol (0.80 g, 2.09 mmol, 16% yield) as off-white gummy solid. *m/z* (ESI): 207.1 (M+H)^+^.

**Step 2: *tert*-Butyl 4-(6-(2-hydroxyphenyl)pyridazin-4-yl)piperazine-1-carboxylate.** A mixture of 2-(5-chloropyridazin-3-yl)phenol (0.60 g, 1.57 mmol), DIPEA (1.37 mL, 7.84 mmol) and *tert*-butyl piperazine-1-carboxylate (1.46 g, 7.84 mmol, Chempure) in DMSO was stirred at 110 °C for 16 h. The reaction mixture was cooled to r.t., diluted with ice-cold water and extracted with EtOAc. The combined organic extracts were washed with brine, dried over Na_2_SO_4_, filtered, concentrated under reduced pressure (rotary evaporator) and purified by column chromatography on silica gel eluting with 0-60% EtOAc in petroleum ether, to provide *tert*-butyl 4-(6-(2-hydroxyphenyl)pyridazin-4-yl)piperazine-1-carboxylate (0.34 g, 0.95 mmol, 61% yield) as light-yellow solid. ^1^H NMR (400 MHz, DMSO-*d*_6_) δ ppm 14.53 (s, 1 H), 8.94 (d, *J*=2.8 Hz, 1 H), 8.10 (dd, *J*=8.4, 1.7 Hz, 1 H), 7.56 (d, *J*=3.0 Hz, 1 H), 7.26 – 7.43 (m, 1 H), 6.86 – 7.00 (m, 2 H), 3.60 – 3.70 (m, 4 H), 3.40 – 3.58 (m, 4 H), 1.44 (s, 9 H). *m/z* (ESI): 357.1 (M+H)^+^.

**Step 3: 2-(5-(Piperazin-1-yl)pyridazin-3-yl)phenol.** A mixture of *tert*-butyl 4-(6-(2-hydroxyphenyl)pyridazin-4-yl)piperazine-1-carboxylate (0.34 g, 0.95 mmol) and TFA (1.0 mL, 13 mmol) in DCM was stirred at r.t. for 12 h. The reaction mixture was concentrated and co-evaporated with diethyl ether to give 2-(5-(piperazin-1-yl)pyridazin-3-yl)phenol, 2,2,2-trifluoroacetate salt (0.35 g, 0.95 mmol, 99% yield) as off-white solid. ^1^H NMR (300 MHz, DMSO-*d*_6_) δ ppm 8.97 – 9.23 (m, 3 H), 7.71 – 7.80 (m, 1 H), 7.62 (br s, 1 H), 7.44 (t, *J*=7.7 Hz, 1 H), 6.93 – 7.11 (m, 2 H), 3.98 (br s, 4 H), 3.31 (br s, 4 H). *m/z* (ESI): 257.2 (M+H)^+^.

**Step 4: Methyl 2-(4-(6-(2-hydroxyphenyl)pyridazin-4-yl)piperazin-1-yl)isonicotinate.** A mixture of 2-(5-(piperazin-1-yl)pyridazin-3-yl)phenol, 2,2,2-trifluoroacetate salt (0.35 g, 0.95 mmol), DIPEA (0.66 mL, 3.79 mmol) and methyl 2-bromoisonicotinate (0.31 g, 1.42 mmol, CombiBlocks) in DMSO was stirred at 110 °C for 16 h. The reaction mixture was cooled to r.t., diluted with ice-cold water and extracted with EtOAc. The combined organic extracts were washed with brine, dried over Na_2_SO_4_, filtered, concentrated and purified by column chromatography on silica gel using a gradient of 0-80% EtOAc in petroleum ether, to provide methyl 2-(4-(6-(2-hydroxyphenyl)pyridazin-4-yl)piperazin-1-yl)isonicotinate (0.080 g, 0.20 mmol, 22% yield) as light-yellow solid. ^1^H NMR (300 MHz, DMSO-*d*_6_) δ ppm 14.56 (s, 1 H), 8.98 (d, *J*=2.8 Hz, 1 H), 8.33 (d, *J*=5.1 Hz, 1 H), 8.04 – 8.17 (m, 1 H), 7.59 (d, *J*=2.9 Hz, 1 H), 7.30 – 7.41 (m, 1 H), 7.28 (s, 1 H), 7.10 (d, *J*=5.1 Hz, 1 H), 6.93 (d, *J*=7.7 Hz, 2 H), 3.88 (s, 3 H), 3.78 (s, 8 H). *m/z* (ESI): 392.1 (M+H)^+^.

**Step 5: 2-(4-(6-(2-Hydroxyphenyl)pyridazin-4-yl)piperazin-1-yl)isonicotinic acid.** A mixture of methyl 2-(4-(6-(2-hydroxyphenyl)pyridazin-4-yl)piperazin-1-yl)isonicotinate (80 mg, 0.20 mmol) and LiOH monohydrate (24 mg, 1.02 mmol) in THF and water was stirred at r.t. for 16 h. The reaction mixture was concentrated under reduced pressure (rotary evaporator), diluted with ice-water and the pH was adjusted to pH 5-6 using 1.5 N HCl. The precipitated solid was filtered and dried to afford 2-(4-(6-(2-hydroxyphenyl)pyridazin-4-yl)piperazin-1-yl)isonicotinic acid (35 mg, 0.093 mmol, 45% yield) as brown solid. ^1^H NMR (400 MHz, DMSO-*d*_6_) δ ppm 9.01 (d, *J*=3.1 Hz, 1 H), 8.31 (d, *J*=5.1 Hz, 1 H), 7.76 (br s, 1 H), 7.57 (d, *J*=3.1 Hz, 1 H), 7.44 (t, *J*=7.8 Hz, 1 H), 7.27 (s, 1 H), 6.83 – 7.18 (m, 3 H), 3.94 (br s, 4 H), 3.73 – 3.87 (m, 4 H). *m/z* (ESI): 378.1 (M+H)^+^.

**Step 6: *N*-((*S*)-1-((2*S*,4*R*)-4-Hydroxy-2-(((*S*)-1-(4-(4-methylthiazol-5-yl) phenyl)ethyl)carbamoyl)pyrrolidin-1-yl)-3,3-dimethyl-1-oxobutan-2-yl)-2-(4-(6-(2-hydroxyphenyl)pyridazin-4-yl)piperazin-1-yl)isonicotinamide (Compound 10).** To a mixture of 2-(4-(6-(2-hydroxyphenyl)pyridazin-4-yl)piperazin-1-yl)isonicotinic acid (35 mg, 0.093 mmol), DIPEA (49 μL, 0.28 mmol) and HATU (53 mg, 0.14 mmol, Spectrochem) in DCM was added (2*S*,4*R*)-1-((*S*)-2-amino-3,3-dimethylbutanoyl)-4-hydroxy-*N*-((*S*)-1-(4-(4-methylthiazol-5-yl)phenyl)ethyl)pyrrolidine-2-carboxamide hydrochloride (**Intermediate 1**, 54 mg, 0.11 mmol) and stirred at r.t. for 16 h. The reaction mixture was diluted with ice-cold water and extracted with DCM. The combined organic extracts were dried over Na_2_SO_4_, filtered, concentrated under reduced pressure (rotary evaporator) and purified by column chromatography on silica gel eluting with 0-10% MeOH in DCM, to provide *N*-((*S*)-1-((2*S*,4*R*)-4-hydroxy-2-(((*S*)-1-(4-(4-methylthiazol-5-yl)phenyl)ethyl)carbamoyl)pyrrolidin-1-yl)-3,3-dimethyl-1-oxobutan-2-yl)-2-(4-(6-(2-hydroxyphenyl)pyridazin-4-yl)piperazin-1-yl)isonicotinamide (**Compound 10**, 20 mg, 0.025 mmol, 27% yield) as off-white solid. ^1^H NMR (DMSO-*d*_6_, 400 MHz) δ ppm 14.60 (s, 1 H), 8.94 – 9.05 (m, 2 H), 8.44 (d, *J*=7.7 Hz, 1 H), 8.35 (d, *J*=9.1 Hz, 1 H), 8.25 (d, *J*=5.2 Hz, 1 H), 8.14 (d, *J*=8.0 Hz, 1 H), 7.62 (s, 1 H), 7.31 – 7.53 (m, 5 H), 7.25 (s, 1 H), 7.06 (d, *J*=5.3 Hz, 1 H), 6.95 (t, *J*=7.6 Hz, 2 H), 5.16 (d, *J*=3.5 Hz, 1 H), 4.89 – 5.03 (m, 1 H), 4.80 (d, *J*=9.0 Hz, 1 H), 4.46 (t, *J*=8.2 Hz, 1 H), 4.32 (br s, 1 H), 3.80 (br s, 8 H), 3.68 (br s, 2 H), 2.47 (s, 3 H), 1.98 – 2.12 (m, 1 H), 1.75 – 1.87 (m, 1 H), 1.39 (d, *J*=7.0 Hz, 3 H), 1.05 (s, 9 H). *m/z* (ESI): 804.3 (M+H)^+^.

**(2*R*,4*R*)-*N*-(2-(4-((4-(3-amino-6-(2-hydroxyphenyl) pyridazin-4-yl)piperazin-1-yl)methyl)phenethoxy)-4-(4-methylthiazol-5-yl)benzyl)-1-((*R*)-2-(1-fluorocyclopropane-1-carboxamido)-3,3-dimethylbutanoyl)-4-hydroxypyrrolidine-2-carboxamide (Compound 11).**

**Compound** **11** (PROTAC 2) was synthesized according to the procedure described in Farnaby *et al. Nat. Chem. Biol*. **2019**, *15*, 672-680.

**Step 1: 2-Hydroxy-4-(4-methylthiazol-5-yl) benzonitrile.** To a solution of 4-methylthiazole (36.9 g, 372 mmol, 1.84 equiv) in *N*-methyl-2-pyrrolidinone (320 mL) was added 4-bromo-2-hydroxybenzonitrile (40 g, 202 mmol 1 equiv), potassium acetate (59.5 g, 606 mmol 3 equiv) followed by palladium(II) acetate (0.91 g, 4.04 mmol, 0.02 equiv) at r.t. The reaction mixture was stirred at 110 °C for 6 h under nitrogen. After completion of reaction, the mixture was quenched with water (500 mL), extracted with EtOAc (2 x 300 mL) and washed with brine (200 mL). The organic layer was dried (Na_2_SO_4_), filtered, and concentrated under reduced pressure. The crude product was triturated with cold methanol (250 mL) and filtered to give 2-hydroxy-4-(4-methylthiazol-5-yl) benzonitrile (20 g, 92 mmol, 46% yield) as white solid. ^1^H NMR (400 MHz, DMSO-*d*_6_) δ ppm 9.74 (s, 1H), 8.15 (s, 1H), 7.10 – 7.40 (m, 2H), 6.91 (s, 1H), 2.41 (s, 3H). *m/z* (ESI): 217 (M+H)^+^.

**Step 2: 2-(Aminomethyl)-5-(4-methylthiazol-5-yl) phenol.** To a solution of 2-hydroxy-4-(4-methylthiazol-5-yl) benzonitrile (20 g, 92 mmol, 1 equiv) in THF (1 L) was added lithium aluminium hydride (1 M in THF, 277 mL, 277 mmol, 3.0 equiv) dropwise at 0 °C. The reaction mixture was stirred at 50 °C for 3 h under nitrogen. After completion of reaction, the mixture was cooled to 0 °C and quenched with Na_2_SO_4_ (34 g) and stirred for 1 h and quenched with water (500 mL), The reaction mixture was filtered and the residue was washed with 10% methanol in DCM (300 mL). The combined filtrate was concentrated under reduced pressure to give 2-(aminomethyl)-5-(4-methylthiazol-5-yl) phenol (10.5 g, 47.7 mmol, 52% yield) as colorless oil. *m/z* (ESI): 221 (M+H)^+^.

**Step 3: *tert*-Butyl (2-hydroxy-4-(4-methylthiazol-5-yl) benzyl) carbamate (Intermediate 2).** To a solution of 2-(aminomethyl)-5-(4-methylthiazol-5-yl) phenol (10.5 g, 0.048 mmol, 1 equiv) in methanol (1.1 L) were added sodium bicarbonate (4.00 g, 0.048 mmol 1.0 equiv) and Boc-anhydride (11.07 mL, 0.048 mmol) at r.t. The reaction mixture was stirred at r.t. for 18 h under nitrogen. After completion of reaction, the mixture was concentrated under reduced pressure. The crude material was absorbed onto a plug of silica gel and purified by column chromatography on silica gel eluting with gradient of 8-10% EtOAc in hexanes to provide *tert*-butyl (2-hydroxy-4-(4-methylthiazol-5-yl) benzyl) carbamate (4.70 g, 7.80 mmol, 32% yield) as white solid. ^1^H NMR (300 MHz, DMSO-*d*_6_) δ ppm 9.75 (s, 1 H), 8.96 (s, 1 H), 7.36 – 7.07 (m, 3 H), 6.90 (s, 1 H), 4.10 (d, *J*= 6.1 Hz, 2 H), 2.45 (s, 3 H), 1.41 (s, 9 H). *m/z* (ESI): 321 (M+H)^+^.

**Step 4: Methyl (2*S*,4*R*)-1-((*S*)-2-((*tert*-butoxycarbonyl)amino)-3,3-dimethylbutanoyl)-4-hydroxypyrrolidine-2-carboxylate.** To a solution of (*S*)-2-((*tert*-butoxycarbonyl)amino)-3,3-dimethylbutanoic acid (20 g, 86 mmol, 1.0 equiv) in DMF (160 mL) was added HATU (34.5 g, 91 mmol 1.05 equiv) and DIPEA (5.3 mL, 30.1 mmol, 3.5 equiv) followed by methyl (2*S*,4*R*)-4-hydroxypyrrolidine-2-carboxylate (15.64 g, 121 mmol, 1.4 equiv) at r.t. The reaction mixture was stirred at r.t. for 18 h under nitrogen. After completion of the reaction, the mixture was quenched with water (500 mL), extracted with EtOAc (2 x 300 mL) and washed with brine (200 mL). The organic layer was dried (Na_2_SO_4_), filtered and concentrated under reduced pressure to give methyl (2*S*,4*R*)-1-((*S*)-2-((*tert*-butoxycarbonyl)amino)-3,3-dimethylbutanoyl)-4-hydroxypyrrolidine-2-carboxylate (21 g, 58.6 mmol, 68% yield) as white solid. The crude compound was used in the subsequent step without purification. *m/z* (ESI): 359 (M+H)^+^.

**Step 5: Methyl ((*S*)-2-((*tert*-butoxycarbonyl) amino)-3,3-dimethylbutanoyl)-*L*-prolinate.** To a solution of methyl (2*S*,4*R*)-1-((*S*)-2-((*tert*-butoxycarbonyl)amino)-3,3-dimethylbutanoyl)-4-hydroxypyrrolidine-2-carboxylate (21 g, 58.6 mmol, 1.0 equiv) in DCM (210 mL) was added 4 M HCl in dioxane (29.3 mL, 117 mmol 2.0 equiv) dropwise at r.t. and stirred for 18 h under nitrogen. The reaction mixture was concentrated under reduced pressure to give methyl ((*S*)-2-((*tert*-butoxycarbonyl) amino)-3,3-dimethylbutanoyl)-*L*-prolinate (21 g, crude) as white solid. The crude was used in the subsequent step without further purification. *m/z* (ESI): 259 (M+H)^+^.

**Step 6: Methyl (2*S*,4*R*)-1-((*S*)-2-(1-fluorocyclopropane-1-carboxamido)-3,3-dimethylbutanoyl)-4-hydroxypyrrolidine-2-carboxylate.** To a solution of 1-fluorocyclopropane-1-carboxylic acid (26.9 g, 116 mmol, 1.0 equiv) in THF (260 mL) were added oxalyl chloride (10.17 mL, 116 mmol, 1.00 equiv) and DMF (0.9 µL, 0.012 mmol 0.001 equiv) at 0 °C and stirred for 2 h. To this reaction mixture was added triethylamine (40.5 mL, 290 mmol, 2.5 equiv) followed by a solution of methyl ((*S*)-2-((*tert*-butoxycarbonyl) amino)-3,3-dimethylbutanoyl)-*L*-prolinate (21 g, 163 mmol, 1.4 equiv) in THF (210 mL) dropwise at 0 °C and stirred at r.t. for 3 h under nitrogen. After completion of reaction, the mixture was concentrated under reduced pressure to give methyl (2*S*,4*R*)-1-((*S*)-2-(1-fluorocyclopropane-1-carboxamido)-3,3-dimethylbutanoyl)-4-hydroxypyrrolidine-2-carboxylate (24 g, 67.0 mmol, 58% yield) as white solid. The crude was used without further purification in the subsequent step. *m/z* (ESI): 345 (M+H)^+^.

**Step 7: (2*S*,4*R*)-1-((*S*)-2-(1-Fluorocyclopropane-1-carboxamido)-3,3-dimethylbutanoyl)-4-hydroxypyrrolidine-2-carboxylic acid (Intermediate 3).** To a solution of methyl (2*S*,4*R*)-1-((*S*)-2-(1-fluorocyclopropane-1-carboxamido)-3,3-dimethylbutanoyl)-4-hydroxypyrrolidine-2-carboxylate (24 g, 69.7 mmol, 1.0 equiv) in methanol (240 mL) was added LiOH (3.51 g, 84 mmol, 1.0 equiv) at r.t. and stirred for 18 h under nitrogen. After completion of reaction, the mixture was concentrated under reduced pressure and acidified to pH 3 using 2 N HCl to give a white suspension. The suspension was filtered, washed with water (100 mL) and dried under vacuum to give (2*S*,4*R*)-1-((*S*)-2-(1-fluorocyclopropane-1-carboxamido)-3,3-dimethylbutanoyl)-4-hydroxypyrrolidine-2-carboxylic acid (**Intermediate 3**, 7.5 g, 22.70 mmol, 33% yield) as white solid. ^1^H NMR (400 MHz, DMSO-*d*_6_) δ ppm 12.68 (s, 1H), 7.27 (dd, *J*= 9.4, 3.0 Hz, 1H), 5.46-4.88 (bs, 1H) 4.60 (d, *J*= 9.4 Hz, 1H), 4.31 (q, *J*= 8.5 Hz, 2H), 3.71 – 3.54 (m, 2H), 2.19 – 2.06 (m, 1H), 1.91 (ddt, *J*= 13.3, 9.3, 5.2 Hz, 1H), 1.41 – 1.31 (m, 2H), 1.23 (ddd, *J*= 16.3, 8.3, 3.3 Hz, 2H), 0.98 (s, 9H). *m/z* (ESI): 331 (M+H)^+^.

**Step 8: Methyl 2-(4-(bromomethyl) phenyl) acetate.** To a solution of 2-(4-(bromomethyl)phenyl)acetic acid (5.00 g, 21.83 mmol, 1.0 equiv) in MeOH (100 mL) was added thionyl chloride (0.80 mL, 10.91 mmol, 0.5 equiv) dropwise at 0 °C. The reaction mixture was stirred at r.t. for 12 h. The reaction mixture was quenched with 10% aqueous sodium bicarbonate solution (30 mL) and extracted with EtOAc (2 x 30 mL). The organic extract was washed with brine (30 mL), dried (Na_2_SO_4_), filtered and concentrated under reduced pressure to give methyl 2-(4-(bromomethyl) phenyl) acetate (4.50 g, 18.51 mmol, 85% yield) as colorless oil. ^1^H NMR (400 MHz, DMSO-*d*_6_) δ ppm 7.32 – 7.45 (dd, *J*= 9.1, 5.9 Hz, 2H), 7.27 (dd, *J*= 9.1, 5.9 Hz, 2H), 4.73 (s, 2H), 3.70 (s, 2H), 3.62 (s, 3H). *m/z* (ESI): no ionization.

**Step 9:** ***tert*-Butyl 4-(4-(2-methoxy-2-oxoethyl) benzyl) piperazine-1-carboxylate.** To a solution of methyl 2-(4-(bromomethyl) phenyl) acetate (4.50 g, 18.51 mmol, 1.0 equiv) in *N,N*-dimethylformamide (45 mL) were added *tert*-butyl piperazine-1-carboxylate (4.83 g, 25.9 mmol, 1.4 equiv) and potassium carbonate (5.12 g, 37.0 mmol, 2.0 equiv) at r.t. and stirred for 4 h. After completion of the reaction (monitored by TLC), the reaction mixture was quenched with cold water (50 mL) and extracted with diethyl ether (2 x 50 mL). The organic extract was dried (Na_2_SO_4_), filtered and concentrated under reduced pressure. The crude material was absorbed onto a plug of silica gel and purified by column chromatography on silica gel eluting with a gradient of 30-35% EtOAc in hexanes to provide *tert*-butyl 4-(4-(2-methoxy-2-oxoethyl) benzyl) piperazine-1-carboxylate (4.50 g, 12.91 mmol, 70% yield) as tan oil. ^1^H NMR (400 MHz, DMSO-*d*_6_) δ ppm 7.10 – 7.37 (m, 4H), 3.66 (s, 2H), 3.61 (s, 3H), 3.45 (s, 2H), 2.65 – 2.70 (t, *J*= 5.1 Hz, 4H), 2.29 (t, *J*= 5.1 Hz, 4H), 1.39 (s, 9H). *m/z* (ESI): 349 (M+H)^+^.

**Step 10:** **Methyl 2-(4-(piperazin-1-ylmethyl) phenyl) acetate hydrochloride.** To the solution of *tert*-butyl 4-(4-(2-methoxy-2-oxoethyl) benzyl) piperazine-1-carboxylate (4.50 g, 12.91 mmol, 1.0 equiv) in DCM (67.5 mL) was added 4 M HCl in dioxane (30 mL, 120 mmol, 9.2 equiv) at 0 °C. The reaction mixture was stirred at 25 °C for 12 h. After completion of the reaction (monitored by TLC), the reaction mixture was concentrated under reduced pressure to give methyl 2-(4-(piperazin-1-ylmethyl) phenyl) acetate hydrochloride (3.50 g, 95% yield) as white solid. ^1^H NMR (400 MHz, DMSO-*d*_6_) δ ppm 11.99 (s, 1H), 9.50 (s, 2H), 7.48 – 7.62 (m, 2H), 7.36 (dd, *J*= 7.9, 2.9 Hz, 2H), 4.30 (s, 1H), 4.01 (d, *J*= 14.1 Hz, 1H), 3.74 (d, *J*= 1.8 Hz, 2H), 3.63 (s, 3H), 3.26 (d, *J*= 12.2 Hz, 2H), 2.97 (s, 1H), 1.41 (s, 3H). *m/z* (ESI): 249 (M+H)^+^.

**Step 11: Methyl 2-(4-((4-(3-amino-6-chloropyridazin-4-yl) piperazin-1-yl) methyl) phenyl) acetate.** To a solution of methyl 2-(4-(piperazin-1-ylmethyl) phenyl) acetate hydrochloride (3.50 g, 12.29 mmol, 1.0 equiv) in DMSO (52.5 mL) were added 4-bromo-6-chloropyridazin-3-amine (3.33 g, 15.98 mmol, 1.3 equiv) and potassium carbonate (8.49 g, 61.5 mmol, 5.0 equiv) and stirred at 110 °C for 12 h. After completion of the reaction, the reaction mixture was quenched with cold water (50 mL) and extracted with EtOAc (2 x 100 mL). The organic extract was dried (Na_2_SO_4_), filtered and concentrated under reduced pressure. The crude material was absorbed onto a plug of silica gel and purified by column chromatography on silica gel eluting with a gradient of 3-5% MeOH in DCM to provide methyl 2-(4-((4-(3-amino-6-chloropyridazin-4-yl) piperazin-1-yl) methyl) phenyl) acetate (3.00 g, 7.98 mmol, 65% yield) as pale yellow oil. ^1^H NMR (400 MHz, DMSO-*d*_6_) δ ppm 7.11 – 7.36 (m, 4H), 6.88 (s, 1H), 6.10 (s, 2H), 3.60 – 3.70 (m, 5H), 3.51 (s, 2H), 3.00 (b s, 4H), 2.55 (t, *J*=5.0 Hz, 4H). *m/z* (ESI): 376.2 (M+H)^+^.

**Step 12: 2-(4-((4-(3-Amino-6-chloropyridazin-4-yl) piperazin-1-yl) methyl) phenyl) ethan-1-ol.** To a solution of methyl 2-(4-((4-(3-amino-6-chloropyridazin-4-yl) piperazin-1-yl) methyl) phenyl) acetate (3.00 g, 7.98 mmol, 1.0 equiv) in tetrahydrofuran (30 mL) and ethanol (60 mL) was added sodium borohydride (1.51 g, 39.9 mmol, 5.0 equiv) in portions at r.t. and stirred at 80 °C for 16 h. After completion of the reaction (monitored by TLC), the mixture was quenched with water (30 mL) and extracted with EtOAc (2 x 30 mL). The organic extract washed with brine (30 mL), dried (Na_2_SO_4_), filtered and concentrated. The crude material was absorbed onto a plug of silica gel and purified by column chromatography on silica gel eluting with a gradient of 5-7% MeOH in DCM to give 2-(4-((4-(3-amino-6-chloropyridazin-4-yl) piperazin-1-yl) methyl) phenyl) ethan-1-ol (2.00 g, 5.75 mmol, 72% yield) as pale yellow solid. ^1^H NMR (400 MHz, DMSO-*d_6_*) δ ppm 7.13 – 7.31 (m, 4H), 6.87 (s, 1H), 6.09 (s, 2H), 4.63 (t, *J*= 5.1 Hz, 1H), 3.53 – 3.65 (m, 2H), 3.49 (s, 2H), 3.00 (s, 4H), 2.71 (t, *J*= 7.1 Hz, 2H), 2.52 – 2.61 (m, 4H). *m/z* (ESI): 376.2 (M+H)^+^.

**Step 13:** ***tert*-Butyl (2-(4-((4-(3-amino-6-chloropyridazin-4-yl)piperazin-1-yl)methyl)phenethoxy)-4-(4-methylthiazol-5-yl)benzyl)carbamate.** To a solution of 2-(4-((4-(3-amino-6-chloropyridazin-4-yl) piperazin-1-yl) methyl) phenyl) ethan-1-ol (1.70 g, 4.86 mmol, 1.0 equiv) in toluene (25.5 mL) was added (tributylphosphoranylidene)acetonitrile (3.54 g, 14.66 mmol, 3.0 equiv) and *tert*-butyl (2-hydroxy-4-(4-methylthiazol-5-yl) benzyl) carbamate **(Intermediate 2**, 2.04 g, 6.35 mmol, 1.3 equiv) at r.t. The reaction mixture was stirred at 80 °C for 12 h. After the completion of reaction (monitored by TLC), the reaction mixture was quenched with cold water (20 mL) and extracted with EtOAc (2 x 30 mL). The organic extract was washed with brine (20 mL), dried (Na_2_SO_4_), filtered and concentrated under reduced pressure. The crude material was absorbed onto a plug of silica gel and purified by column chromatography on silica gel, eluting with a gradient of 3-6% MeOH in DCM to give *tert*-butyl (2-(4-((4-(3-amino-6-chloropyridazin-4-yl)piperazin-1-yl)methyl)phenethoxy)-4-(4-methylthiazol-5-yl)benzyl)carbamate (2.00 g, 3.05 mmol, 62% yield) as tan solid. ^1^H NMR (400 MHz, *DMSO-d_6_*) δ ppm 8.98 (s, 1H), 7.25 – 7.41 (m, 4H), 7.18 (d, *J*= 8.0 Hz, 2H), 7.03 (d, *J*= 6.2 Hz, 2H), 6.87 (s, 1H), 6.09 (s, 2H), 4.26 (s, 2H), 4.07 (d, *J*= 6.2 Hz, 2H), 3.50 (s, 2H), 3.0 – 3.15 (m, 6H), 2.46 (s, 7H), 1.40 (s, 9H). *m/z* (ESI): 649.9 (M+H)^+^.

**Step 14:** **4-(4-(4-(2-(2-(Aminomethyl)-5-(4-methylthiazol-5-yl) phenoxy)ethyl) benzyl)piperazin-1-yl)-6-chloropyridazin-3-amine.** To the solution of *tert*-butyl (2-(4-((4-(3-amino-6-chloropyridazin-4-yl)piperazin-1-yl)methyl)phenethoxy)-4-(4-methylthiazol-5-yl)benzyl)carbamate (0.50 g, 0.77 mmol, 1.0 equiv) in DCM (10 mL) was added TFA (0.18 mL, 2.31 mmol, 3.0 equiv) at 0 °C. The reaction mixture was stirred at 0 °C for 2 h. After completion of the reaction (monitored by TLC), the mixture was concentrated under reduced pressure to give 4-(4-(4-(2-(2-(aminomethyl)-5-(4-methylthiazol-5-yl) phenoxy)ethyl) benzyl)piperazin-1-yl)-6-chloropyridazin-3-amine (1 g, crude) as pale yellow oil. The crude compound was used in the subsequent step without purification. ^1^H NMR (300 MHz, DMSO-*d*_6_) δ ppm 10.37 (s, 1H), 9.04 (s, 1H), 8.14 (s, 2H), 7.46 (d, *J*= 14.1 Hz, 3H), 7.25 (s, 1H), 7.09 – 7.17 (m, 1H), 4.36 (dd, *J*= 13.8, 7.2 Hz, 4H), 3.96 (d, *J*= 5.8 Hz, 2H), 3.61 (d, *J*=17.4 Hz, 2H), 3.43 (s, 2H), 2.92 – 3.21 (m, 4H), 2.47 (s, 2H), 1.54 (s, 3H). *m/z* (ESI): 550.2 (M+H)^+^.

**Step 15:** **(2*R*,4*R*)-*N*-(2-(4-((4-(3-Amino-6-chloropyridazin-4-yl) piperazin-1-yl) methyl)phenethoxy)-4-(4-methylthiazol-5-yl) benzyl)-1-((*R*)-2-(1-fluorocyclopropane-1-carboxamido)-3,3-dimethylbutanoyl)-4-hydroxypyrrolidine-2-carboxamide.** To a solution of 4-(4-(4-(2-(2-(aminomethyl)-5-(4-methylthiazol-5-yl) phenoxy)ethyl) benzyl)piperazin-1-yl)-6-chloropyridazin-3-amine (0.50 g, 0.91 mmol, 1.0 equiv) in *N,N*-dimethylformamide (1 mL) were added (2*S*,4*R*)-1-((*S*)-2-(1-fluorocyclopropane-1-carboxamido)-3,3-dimethylbutanoyl)-4-hydroxypyrrolidine-2-carboxylic acid (**Intermediate 3**, 0.30 g, 0.91 mmol, 1.0 equiv), HATU (0.42 g, 1.09 mmol, 1.2 equiv) and DIPEA (0.79 mL, 4.54 mmol, 5.0 equiv) at r.t. and stirred for 18 h. The reaction mixture was quenched with water (2 mL) and extracted with EtOAc (2 x 5 mL). The organic extract was washed with brine (5 mL), dried (Na_2_SO_4_), filtered and concentrated under reduced pressure. The crude material was adsorbed onto a plug of silica gel and purified by column chromatography on silica gel eluting with a gradient of 3-6% MeOH in DCM to provide (2*R*,4*R*)-*N*-(2-(4-((4-(3-amino-6-chloropyridazin-4-yl) piperazin-1-yl) methyl)phenethoxy)-4-(4-methylthiazol-5-yl) benzyl)-1-((*R*)-2-(1-fluorocyclopropane-1-carboxamido)-3,3-dimethylbutanoyl)-4-hydroxypyrrolidine-2-carboxamide (2.00 g, 3.05 mmol, 62% yield) as tan solid. ^1^H NMR (400 MHz, DMSO-*d_6_*) δ ppm 8.98 (s, 1H), 8.48 (t, *J*= 6.1 Hz, 1H), 7.20 – 7.50 (m, 6H), 7.02 (s, 1H), 6.94 (d, *J*= 7.9 Hz, 1H), 6.87 (s, 1H), 6.09 (s, 2H), 5.76 (s, 1H), 5.18 (d, *J*= 3.6 Hz, 1H), 4.46 – 4.63 (m, 2H), 4.05 – 4.42 (m, 6H), 3.62 (d, *J*= 6.3 Hz, 3H), 3.50 (s, 2H), 2.87 – 3.20 (m, 4H), 2.45 (s, 3H), 2.03 – 2.15 (m, 2H), 1.92 (q, *J*= 8.4, 6.8 Hz, 1H), 1.37 (dd, *J*= 18.9, 8.9 Hz, 3H), 1.22 (ddt, *J*= 18.1, 14.0, 6.6 Hz, 3H), 0.96 (s, 9H). *m/z* (ESI): 862.1 (M+H)^+^.

**Step 16: (2*R*,4*R*)-*N*-(2-(4-((4-(3-Amino-6-(2-hydroxyphenyl) pyridazin-4-yl)piperazin-1-yl)methyl)phenethoxy)-4-(4-methylthiazol-5-yl)benzyl)-1-((*R*)-2-(1-fluorocyclopropane-1-carboxamido)-3,3-dimethylbutanoyl)-4-hydroxypyrrolidine-2-carboxamide (Compound 11).** To a solution of (2*R*,4*R*)-*N*-(2-(4-((4-(3-amino-6-chloropyridazin-4-yl) piperazin-1-yl) methyl)phenethoxy)-4-(4-methylthiazol-5-yl) benzyl)-1-((*R*)-2-(1-fluorocyclopropane-1-carboxamido)-3,3-dimethylbutanoyl)-4-hydroxypyrrolidine-2-carboxamide (0.30 g, 0.35 mmol, 1.0 equiv) in 1,4-dioxane (6 mL) and water (0.12 mL) were added (2-hydroxyphenyl)boronic acid (96 mg, 0.70 mmol, 2.0 equiv), potassium carbonate (96 mg, 0.70 mmol, 2.0 equiv) at r.t. The reaction mixture was degassed and purged with nitrogen for 5 min and added PdCl_2_(dppf)-CH_2_Cl_2_ adduct (14 mg, 0.017 mmol, 0.05 equiv). The reaction mixture was stirred at 100 °C for 4 h. After the completion of reaction (monitored by TLC), the mixture was diluted with EtOAc (10 mL) and filtered through celite. The filtrate was concentrated under reduced pressure. The crude material was purified by prep. HPLC purification (Agilent 1260 infinity; Mobile phase: A: 10 mM ammonium acetate in water B: MeCN with a flow rate of 15 mL/min) to give (2*R*,4*R*)-*N*-(2-(4-((4-(3-amino-6-(2-hydroxyphenyl) pyridazin-4-yl)piperazin-1-yl)methyl)phenethoxy)-4-(4-methylthiazol-5-yl)benzyl)-1-((*R*)-2-(1-fluorocyclopropane-1-carboxamido)-3,3-dimethylbutanoyl)-4-hydroxypyrrolidine-2-carboxamide (**Compound 11**, 74 mg, 23% yield) as white solid. ^1^H NMR (400 MHz, DMSO-*d*_6_) δ ppm 14.23 (s, 1H), 8.98 (s, 1H), 8.45 – 8.52 (m, 1H), 7.86 – 7.95 (m, 1H), 7.50 (s, 1H), 7.40 (d, *J*= 7.8 Hz, 1H), 7.18 – 7.35 (m, 6H), 7.02 (d, *J*= 1.7 Hz, 1H), 6.92 – 6.96 (m, 1H), 6.88 (dd, *J*= 7.7, 6.4 Hz, 2H), 6.24 (s, 2H), 5.17 (d, *J*= 3.5 Hz, 1H), 4.46 – 4.63 (m, 2H), 4.28 (t, *J*= 23.6 Hz, 4H), 4.13 (dd, *J*= 16.6, 5.5 Hz, 1H), 3.57 – 3.66 (m, 2H), 3.54 (s, 2H), 3.09 (d, *J*= 8.8 Hz, 6H), 2.55 – 2.65 (m, 3H), 2.45 (s, 3H), 2.04 – 2.15 (m, 1H), 1.93 (d, *J*= 10.9 Hz, 1H), 1.37 (dd, *J*= 18.5, 9.5 Hz, 2H), 1.19 – 1.27 (m, 3H), 0.94 (s, 9H). *m/z* (ESI): 920.0 (M+H)^+^

**Synthesis of BRD4-VHL PROTACs**

**General Synthetic Scheme for the Synthesis of Compounds 12-16.**

The syntheses of compounds **12-16** have been previously reported (Wurz, R. P. *et al. J. Med. Chem.* **2018**, *61*, 453-461).

| **(*S*)-*N*-(2-Azidoethyl)-2-(4-(4-chlorophenyl)-2,3,9-trimethyl-6*H*-thieno[3,2-*f*][1,2,4]triazolo[4,3-*a*][1,4]diazepin-6-yl)acetamide.** |
| --- |

**Step 1. (*S*)-2-(4-(4-Chlorophenyl)-2,3,9-trimethyl-6*H*-thieno[3,2-*f*][1,2,4]triazolo[4,3-*a*][1,4]diazepin-6-yl)acetic acid.** (*S*)-*tert*-Butyl 2-(4-(4-chlorophenyl)-2,3,9-trimethyl-6*H*-thieno[3,2-*f*][1,2,4]triazolo[4,3-*a*][1,4]diazepin-6-yl)acetate (Selleckchem.com, Cat. No. S7110, 0.90 g, 1.96 mmol) was treated with DCM (6 mL) and TFA (4 mL, 51.9 mmol) and allowed to stir at r.t. for 2 h. The reaction mixture was concentrated to dryness affording crude (*S*)-2-(4-(4-chlorophenyl)-2,3,9-trimethyl-6*H*-thieno[3,2-*f*][1,2,4]triazolo[4,3-*a*][1,4]diazepin-6-yl)acetic acid mono-TFA salt (1:1) (1.01 g, 1.96 mmol) as a viscous yellow tar (assuming quantitative yield). ^1^H NMR (400 MHz, *DMSO-d*_6_) δ ppm 3.49 (2 H, dd, *J*=16.6, 6.8 Hz), 3.37 (1 H, dd, *J*=16.6, 7.4 Hz), 2.65 - 2.70 (3 H, m), 2.14 (1 H, s). ^19^F NMR (376 MHz, *DMSO-d*_6_) δ ppm -74.94 (1 F, s) - assume mono-TFA salt. *m/z* (ESI, +ve) 401.1 (M+H)^+^.

**Step 2. (*S*)-*N*-(2-Azidoethyl)-2-(4-(4-chlorophenyl)-2,3,9-trimethyl-6*H*-thieno[3,2-*f*][1,2,4]triazolo[4,3-*a*][1,4]diazepin-6-yl)acetamide**. (*S*)-2-(4-(4-Chlorophenyl)-2,3,9-trimethyl-6*H*-thieno[3,2-*f*][1,2,4]triazolo[4,3-*a*][1,4]diazepin-6-yl)acetic acid mono-TFA salt (1:1) (1.01 g, 1.96 mmol) was treated with 2-azidoethanamine hydrochloride (0.29 g, 2.36 mmol, Enamine), *N,N*-dimethylformamide (10 mL) and DIPEA (1.72 mL, 9.82 mmol) followed by HATU (0.93 g, 2.45 mmol) in one portion. The reaction mixture was then allowed to stir at r.t. for 30 min. LC-MS analysis of the reaction mixture indicated ca. 50% conversion to the desired product *m/z* (ESI, +ve) 469.1 (M+H)^+^. The reaction mixture was treated with an additional DIPEA (1.0 mL) and after another 30 min, LC-MS analysis indicated clean conversion to the desired product. The reaction mixture was treated with a saturated solution of NaHCO_3_ and extracted with EtOAc (2 x 50 mL), washed with brine (3 x 25 mL) and dried over MgSO_4_, filtered and concentrated. The crude residue was purified by column chromatography on silica gel using a gradient of 0-12% MeOH in DCM affording (*S*)-*N*-(2-azidoethyl)-2-(4-(4-chlorophenyl)-2,3,9-trimethyl-6*H*-thieno[3,2-*f*][1,2,4]triazolo[4,3-*a*][1,4]diazepin-6-yl)acetamide (0.93 g, 1.99 mmol, >99% yield) as light yellow foam. ^1^H NMR (400 MHz, *DMSO-d*_6_) δ ppm 7.41 - 7.51 (1 H, m), 3.34 - 3.44 (1 H, m), 3.31 - 3.33 (8 H, m), 3.17 - 3.29 (1 H, m), 2.68 - 2.71 (3 H, m), 2.59 (1 H, s), 2.41 (1 H, s), 1.62 (1 H, s). ^13^C NMR (151 MHz, *DMSO-d*_6_) δ ppm 170.44, 165.07, 163.52, 162.78, 155.55, 150.30, 137.24, 135.69, 132.74, 131.18, 130.64, 130.31, 130.05, 128.91, 54.25, 50.52, 38.72, 38.65, 38.08, 36.25, 31.24, 28.28, 14.51, 13.15, 11.77. *m/z* (ESI, +ve) 469.1 (M+H)^+^.

**Representative Procedure for the Synthesis of VHL-alkyne building blocks.**

**(2*S*,4*R*)-1-((*S*)-3,3-Dimethyl-2-(2-(prop-2-yn-1-yloxy)acetamido)butanoyl)-4-hydroxy-*N*-(4-(4-methylthiazol-5-yl)benzyl)pyrrolidine-2-carboxamide.**

To a solution of 2-(prop-2-yn-1-yloxy)acetic acid (0.68 g, 6.00 mmol, 1 equiv.) in DCM (50 mL) was added (COCl)_2_ (1.90 g, 15.0 mmol, 1.31 mL, 2.5 equiv.) followed by DMF (0.44 g, 6 mmol, 0.5 mL, 1.0 equiv.) dropwise. After addition, the mixture was stirred at 15 °C for 1 h, then the mixture was concentrated under vacuum. The crude acid chloride was diluted with THF (50 mL), (2*S*,4*R*)-1-[(2*S*)-2-amino-3,3-dimethylbutanoyl]-4-hydroxy-*N*-{[4-(4-methyl-1,3-thiazol-5-yl)phenyl]methyl}pyrrolidine-2-carboxamide hydrochloride (2.80 g, 6.00 mmol, 1.0 equiv., Enamine) and triethylamine (3.04 g, 30 mmol, 4.16 mL, 5.0 equiv.) then the mixture was stirred at 40 °C for 12 h. The mixture was quenched with water (80 mL), extracted with EtOAc (2 x 80 mL), the combined organic phase was washed with brine (100 mL), dried over anhydrous Na_2_SO_4_, filtered and concentrated under vacuum. The residue was purified by prep-HPLC affording (2*S*,4*R*)-1-((*S*)-3,3-dimethyl-2-(2-(prop-2-yn-1-yloxy)acetamido)butanoyl)-4-hydroxy-*N*-(4-(4-methylthiazol-5-yl)benzyl)pyrrolidine-2-carboxamide (1.40 g, 2.64 mmol, 44% yield) as white solid. ^1^H NMR (400 MHz, *DMSO-d*_6_) δ ppm 9.03 (1 H, s), 8.64 (1 H, t, *J*=5.9 Hz), 7.47 - 7.54 (1 H, m), 7.45 (4 H, s), 5.20 (1 H, br s), 4.60 (1 H, d, *J*=9.5 Hz), 4.44 - 4.52 (1 H, m), 4.37 - 4.44 (2 H, m), 4.25 - 4.35 (4 H, m), 3.97 - 4.12 (2 H, m), 3.61 - 3.75 (2 H, m), 3.56 (1 H, t, *J*=2.3 Hz), 2.10 (1 H, br s), 1.95 (1 H, s), 0.93 - 1.03 (9 H, m). ^13^C NMR (151 MHz, *DMSO-d*_6_) δ ppm 172.23, 169.60, 168.42, 151.93, 148.23, 139.92, 131.61, 130.18, 129.36, 129.17, 127.94, 79.99, 78.44, 69.36, 68.50, 59.21, 58.41, 57.06, 56.27, 42.16, 38.39, 36.23, 26.75, 26.67, 16.41. *m/z* (ESI, +ve) 527.2 (M+H)^+^.

**(2*S*,4*R*)-1-((*S*)-3,3-Dimethyl-2-(2-(2-(prop-2-yn-1-yloxy)ethoxy)acetamido)butanoyl)-4-hydroxy-*N*-(4-(4-methylthiazol-5-yl)benzyl)pyrrolidine-2-carboxamide.**

Prepared according to the representative procedure for the synthesis of VHL-alkyne building blocks affording (2*S*,4*R*)-1-((*S*)-3,3-dimethyl-2-(2-(2-(prop-2-yn-1-yloxy)ethoxy)acetamido)butanoyl)-4-hydroxy-*N*-(4-(4-methylthiazol-5-yl)benzyl)pyrrolidine-2-carboxamide (2.00 g, 40% yield) as light yellow syrup. ^1^H NMR (400 MHz, *DMSO-d*_6_) δ ppm 8.98 (1 H, s), 8.60 (1 H, t, *J*=6.1 Hz), 7.48 (1 H, br d, *J*=9.5 Hz), 7.40 (4 H, s), 5.15 (1 H, d, *J*=3.5 Hz), 4.57 (1 H, d, *J*=9.5 Hz), 4.39 - 4.47 (2 H, m), 4.37 (1 H, br d, *J*=13.7 Hz), 4.12 - 4.30 (4 H, m), 3.96 (2 H, s), 3.56 - 3.70 (7 H, m), 3.36 - 3.44 (1 H, m), 1.90 (1 H, br s), 0.88 - 1.00 (9 H, m). ^13^C NMR (151 MHz, *DMSO-d*_6_) δ ppm 172.25, 169.61, 169.09, 151.92, 148.23, 139.93, 131.61, 130.18, 129.35, 129.16, 128.60, 127.92, 80.64, 77.73, 77.70, 70.67, 70.51, 70.03, 69.36, 68.66, 59.21, 58.02, 57.04, 56.21, 42.15, 40.92, 38.39, 36.17, 26.76 , 26.66, 16.44, 16.41. *m/z* (ESI, +ve) 571.2 (M+H)^+^.

**(2*S*,4*R*)-1-((*S*)-2-(*tert*-Butyl)-4-oxo-6,9,12-trioxa-3-azapentadec-14-yn-1-oyl)-4-hydroxy-*N*-(4-(4-methylthiazol-5-yl)benzyl)pyrrolidine-2-carboxamide.**

Prepared according to the representative procedure for the synthesis of VHL-alkyne building blocks affording (2*S*,4*R*)-1-((*S*)-2-(*tert*-butyl)-4-oxo-6,9,12-trioxa-3-azapentadec-14-yn-1-oyl)-4-hydroxy-*N*-(4-(4-methylthiazol-5-yl)benzyl)pyrrolidine-2-carboxamide (1.00 g, 40% yield) as light yellow syrup. ^1^H NMR (400 MHz, *DMSO-d*_6_) δ ppm 9.03 (1 H, s), 8.64 (1 H, br t, *J*=6.0 Hz), 7.41 - 7.51 (6 H, m), 5.19 (1 H, d, *J*=3.5 Hz), 4.61 (1 H, d, *J*=9.5 Hz), 4.36 - 4.52 (4 H, m), 4.24 - 4.36 (2 H, m), 4.12 - 4.22 (3 H, m), 4.01 (2 H, s), 3.56 - 3.74 (13 H, m), 3.40 - 3.52 (3 H, m), 2.65 (1 H, br s), 2.57 - 2.60 (21 H, m), 2.03 - 2.25 (1 H, m), 1.95 (1 H, ddd, *J*=12.9, 8.7, 4.6 Hz), 0.93 - 1.04 (9 H, m). ^13^C NMR (151 MHz, *DMSO-d*_6_) δ ppm 172.23, 169.61, 169.05, 151.93, 148.23, 139.92, 131.61, 130.19, 129.35, 129.17, 127.94, 80.77, 77.55, 70.89, 70.07, 70.03, 69.35, 69.01, 68.99, 59.20, 57.97, 57.04, 56.18, 42.16, 38.39, 36.18, 26.81, 26.75, 26.66, 16.44, 16.40. *m/z* (ESI, +ve) 615.2 (M+H)^+^.

**(2*S*,4*R*)-1-((*S*)-2-(*tert*-Butyl)-4-oxo-6,9,12,15-tetraoxa-3-azaoctadec-17-yn-1-oyl)-4-hydroxy-*N*-(4-(4-methylthiazol-5-yl)benzyl)pyrrolidine-2-carboxamide.**

Prepared according to the representative procedure for the synthesis of VHL-alkyne building blocks affording (2*S*,4*R*)-1-((*S*)-2-(*tert*-butyl)-4-oxo-6,9,12,15-tetraoxa-3-azaoctadec-17-yn-1-oyl)-4-hydroxy-*N*-(4-(4-methylthiazol-5-yl)benzyl)pyrrolidine-2-carboxamide (3.60 g, 32% yield) as light yellow syrup. ^1^H NMR (400 MHz, *DMSO-d*_6_) δ ppm 8.98 (1 H, s), 8.59 (1 H, t, *J*=6.1 Hz), 7.40 (5 H, s), 5.15 (1 H, d, *J*=3.5 Hz), 4.56 (1 H, d, *J*=9.5 Hz), 4.39 - 4.47 (2 H, m), 4.32 - 4.39 (2 H, m), 4.19 - 4.31 (2 H, m), 4.10 - 4.15 (3 H, m), 3.96 (2 H, s), 3.62 - 3.69 (3 H, m), 3.52 - 3.62 (11 H, m), 3.38 - 3.42 (2 H, m), 2.53 - 2.55 (35 H, m), 2.42 - 2.47 (4 H, m), 2.05 (1 H, br dd, *J*=12.5, 7.8 Hz), 1.90 (1 H, ddd, *J*=12.8, 8.7, 4.5 Hz), 0.89 - 0.99 (9 H, m).^13^C NMR (151 MHz, *DMSO-d*_6_) δ ppm 172.23, 169.61, 169.05, 151.93, 148.23, 139.92, 131.61, 130.19, 129.17, 128.61, 127.94, 80.77, 77.55, 70.89, 70.07, 70.03, 70.00, 69.35, 69.01, 68.99, 59.20, 57.97, 57.04, 56.18, 42.16, 38.39, 36.18, 26.76, 26.66, 16.40. *m/z* (ESI, +ve) 637.2 (M+H)^+^.

**(2*S*,4*R*)-1-((*S*)-2-(*tert*-Butyl)-4-oxo-6,9,12,15,18-pentaoxa-3-azahenicos-20-yn-1-oyl)-4-hydroxy-*N*-(4-(4-methylthiazol-5-yl)benzyl)pyrrolidine-2-carboxamide.**

Prepared according to the representative procedure for the synthesis of VHL-alkyne building blocks affording (2*S*,4*R*)-1-((*S*)-2-(*tert*-butyl)-4-oxo-6,9,12,15,18-pentaoxa-3-azahenicos-20-yn-1-oyl)-4-hydroxy-*N*-(4-(4-methylthiazol-5-yl)benzyl)pyrrolidine-2-carboxamide (6.00 g, 39% yield) as light yellow oil. ^1^H NMR (400 MHz, *DMSO-d*_6_) δ ppm 8.98 (1 H, s), 8.59 (1 H, br t, *J*=5.9 Hz), 7.34 - 7.46 (6 H, m), 5.15 (1 H, d, *J*=3.3 Hz), 4.56 (1 H, d, *J*=9.7 Hz), 4.40 - 4.47 (2 H, m), 4.31 - 4.39 (2 H, m), 4.19 - 4.29 (2 H, m), 4.13 (3 H, d, *J*=2.3 Hz), 3.96 (2 H, s), 3.57 - 3.69 (7 H, m), 3.44 - 3.57 (18 H, m), 3.38 - 3.42 (2 H, m), 2.60 (1 H, br s), 2.52 - 2.55 (21 H, m), 2.42 - 2.46 (4 H, m), 2.01 - 2.11 (1 H, m), 1.90 (1 H, ddd, *J*=12.9, 8.8, 4.5 Hz), 0.88 - 0.99 (9 H, m). ^13^C NMR (151 MHz, *DMSO-d*_6_) δ ppm 172.24, 169.61, 169.07, 151.93, 148.23, 139.92, 131.61, 130.17, 129.35, 129.16, 128.61, 127.94, 80.81, 77.55, 70.94, 70.27, 70.23, 70.14, 69.35, 68.98, 59.21, 57.96, 57.04, 56.16, 42.16, 40.92, 38.39, 36.19, 26.81, 26.75, 26.65, 16.39. *m/z* (ESI, +ve) 703.4 (M+H)^+^.

**Representative Procedure for the “Click Ligation”.**

In a 3-mL vial was weighed (*2S,4R*)-1-((*S*)-3,3-dimethyl-2-(2-(prop-2-yn-1-yloxy)acetamido)butanoyl)-4-hydroxy-*N*-(4-(4-methylthiazol-5-yl)benzyl)pyrrolidine-2-carboxamide (51 mg, 0.097 mmol), (+)-sodium l-ascorbate (3.8 mg, 0.019 mmol), anhydrous copper(II) sulfate powder (3.1 mg, 0.019 mmol) and (*S*)-*N*-(2-azidoethyl)-2-(4-(4-chlorophenyl)-2,3,9-trimethyl-6*H*-thieno[3,2-*f*][1,2,4]triazolo[4,3-*a*][1,4]diazepin-6-yl)acetamide (45.4 mg, 0.097 mmol). The reaction mixture was treated with THF (1 mL, ca. 0.1 M) and 3-4 drops of water then the headspace of the vial was purged briefly with argon and stirred at r.t. for 16 h. LC-MS analysis of the crude reaction mixture indicated clean conversion to the desired product *m/z* (ESI, +ve) 995.4 (M+H)^+^. The reaction mixture was then purified by column chromatography on silica gel using a gradient of 0-20% MeOH in DCM affording the desired (2*S*,4*R*)-1-((*S*)-2-(2-((1-(2-(2-((*S*)-4-(4-chlorophenyl)-2,3,9-trimethyl-6*H*-thieno[3,2-*f*][1,2,4]triazolo[4,3-*a*][1,4]diazepin-6-yl)acetamido)ethyl)-1*H*-1,2,3-triazol-4-yl)methoxy)acetamido)-3,3-dimethylbutanoyl)-4-hydroxy-*N*-(4-(4-methylthiazol-5-yl)benzyl)pyrrolidine-2-carboxamide (**Compound 12**, 76 mg, 0.076 mmol, 79% yield) as white amorphous solid.

**(2*S*,4*R*)-1-((*S*)-2-(2-((1-(2-(2-((*S*)-4-(4-chlorophenyl)-2,3,9-trimethyl-6*H*-thieno[3,2-*f*][1,2,4]triazolo[4,3-*a*][1,4]diazepin-6-yl)acetamido)ethyl)-1*H*-1,2,3-triazol-4-yl)methoxy)acetamido)-3,3-dimethylbutanoyl)-4-hydroxy-*N*-(4-(4-methylthiazol-5-yl)benzyl)pyrrolidine-2-carboxamide (Compound 12).**

Prepared according to the general “click ligation” method affording **Compound 12** (76 mg, 0.076 mmol, 79% yield) as white amorphous solid. ^1^H NMR (400 MHz, *DMSO-d*_6_) δ ppm 8.97 (1 H, s), 8.58 (1 H, t, *J*=5.9 Hz), 8.45 (1 H, t, *J*=5.6 Hz), 8.20 (1 H, s), 7.47 (1 H, s), 7.48 (2 H, d, *J*=7.9 Hz), 7.35 - 7.45 (7 H, m), 5.15 (1 H, d, *J*=3.5 Hz), 4.63 (2 H, s), 4.56 (1 H, d, *J*=9.6 Hz), 4.50 (1 H, t, *J*=7.1 Hz), 4.31 - 4.47 (5 H, m), 4.20 - 4.31 (1 H, m), 4.00 (2 H, d, *J*=4.7 Hz), 3.53 - 3.69 (4 H, m), 3.21 - 3.27 (2 H, m), 2.55 - 2.63 (3 H, m), 2.43 - 2.46 (3 H, m), 2.41 (3 H, s), 1.98 - 2.10 (1 H, m), 1.90 (1 H, td, *J*=8.7, 4.4 Hz), 1.62 (3 H, s), 0.87 - 0.97 (9 H, m). ^13^C NMR (151 MHz, *DMSO-d*_6_) δ ppm 172.20, 170.59, 169.62, 168.74, 163.57, 155.52, 151.91, 150.34, 148.23, 143.51, 139.89, 137.22, 135.67, 132.71, 131.61, 131.15, 130.69, 130.35, 130.18, 130.04, 129.35, 129.18, 128.92, 127.93, 125.08, 69.35, 69.03, 64.21, 59.20, 57.05, 56.21, 54.19, 49.32, 42.16, 38.38, 38.03, 36.17, 26.73, 26.66, 16.40, 14.53, 13.16, 11.78. *m/z* (ESI, +ve) 995.3 (M+H)^+^.

**(2*S*,4*R*)-1-((*S*)-2-(2-(2-((1-(2-(2-((*S*)-4-(4-chlorophenyl)-2,3,9-trimethyl-6*H*-thieno[3,2-*f*][1,2,4]triazolo[4,3-*a*][1,4]diazepin-6-yl)acetamido)ethyl)-1*H*-1,2,3-triazol-4-yl)methoxy)ethoxy)acetamido)-3,3-dimethylbutanoyl)-4-hydroxy-*N*-(4-(4-methylthiazol-5-yl)benzyl)pyrrolidine-2-carboxamide (Compound 13).**

Prepared according to the general “click ligation” method affording **Compound 13** (62 mg, 0.059 mmol, 60% yield) as off-white amorphous solid. ^1^H NMR (400 MHz, *DMSO-d*_6_) δ ppm 8.95 - 8.98 (1 H, m), 8.59 (1 H, t, *J*=6.0 Hz), 8.43 (1 H, br t, *J*=5.7 Hz), 8.14 (1 H, s), 7.45 - 7.52 (3 H, m), 7.33 - 7.45 (6 H, m), 5.15 (1 H, d, *J*=3.5 Hz), 4.52 - 4.61 (3 H, m), 4.38 - 4.52 (4 H, m), 4.36 (2 H, br d, *J*=6.1 Hz), 4.20 - 4.32 (1 H, m), 3.95 (2 H, s), 3.47 - 3.70 (8 H, m), 3.19 - 3.27 (2 H, m), 3.17 (1 H, d, *J*=5.1 Hz), 2.59 (3 H, s), 2.39 - 2.45 (6 H, m), 1.98 - 2.10 (1 H, m), 1.90 (1 H, ddd, *J*=12.9, 8.7, 4.5 Hz), 1.62 (3 H, s), 0.87 - 0.99 (9 H, m). ^13^C NMR (151 MHz, *DMSO-d*_6_) δ ppm 172.22, 171.79, 170.59, 170.00, 169.66, 169.08, 168.23, 163.58, 155.52, 151.98, 151.91, 150.34, 148.22, 144.23, 139.90, 137.21, 135.67, 132.71, 131.59, 131.16, 130.69, 130.48, 130.35, 130.18, 130.04, 129.35, 129.16, 128.92, 128.59, 127.93, 124.75, 124.71, 108.98, 70.82, 70.71, 70.12, 70.05, 69.86, 69.34, 69.03, 68.97, 67.44, 64.12, 64.02, 59.42, 59.21, 57.04, 56.21, 55.96, 55.38, 54.17, 49.25, 49.07, 38.40, 38.01, 36.60, 36.15, 26.77, 26.74, 26.65, 22.71, 18.43, 16.43, 16.38, 14.52, 13.15, 11.78. *m/z* (ESI, +ve) 1039.4 (M+H)^+^.

**(2*S,*4*R*)-1-((*S*)-12-(*tert*-butyl)-1-(1-(2-(2-((*S*)-4-(4-chlorophenyl)-2,3,9-trimethyl-6*H*-thieno[3,2-*f*][1,2,4]triazolo[4,3-*a*][1,4]diazepin-6-yl)acetamido)ethyl)-1*H*-1,2,3-triazol-4-yl)-10-oxo-2,5,8-trioxa-11-azatridecan-13-oyl)-4-hydroxy-*N*-(4-(4-methylthiazol-5-yl)benzyl)pyrrolidine-2-carboxamide (Compound 14)**

Prepared according to the general “click ligation” method affording **Compound 14** (88 mg, 0.081 mmol, 72% yield) as off-white amorphous solid. ^1^H NMR (400 MHz, *DMSO-d*_6_) δ ppm 8.94 - 9.00 (1 H, m), 8.59 (1 H, t, *J*=6.0 Hz), 8.43 (1 H, t, *J*=5.7 Hz), 8.07 - 8.14 (1 H, m), 7.46 - 7.51 (2 H, m), 7.30 - 7.45 (7 H, m), 5.14 (1 H, d, *J*=3.5 Hz), 4.56 (1 H, d, *J*=9.8 Hz), 4.47 - 4.53 (3 H, m), 4.39 - 4.47 (4 H, m), 4.36 (2 H, br d, *J*=6.3 Hz), 4.25 (1 H, br dd, *J*=15.7, 5.8 Hz), 4.09 (3 H, q, *J*=5.3 Hz), 3.96 (2 H, s), 3.64 - 3.70 (1 H, m), 3.53 - 3.63 (11 H, m), 3.23 (2 H, br d, *J*=7.0 Hz), 3.17 (9 H, d, *J*=5.3 Hz), 2.59 (3 H, s), 2.43 (3 H, s), 2.41 (3 H, s), 2.04 (1 H, br d, *J*=8.4 Hz), 1.85 - 1.98 (1 H, m), 1.62 (3 H, s), 1.25 (2 H, br d, *J*=8.2 Hz), 0.93 (9 H, s). ^13^C NMR (151 MHz, *DMSO-d*_6_) δ ppm 172.22, 170.57, 169.62, 169.07, 163.58, 155.53, 151.92, 150.34, 148.23, 144.32, 139.91, 137.22, 135.67, 132.72, 131.61, 131.16, 130.69, 130.35, 130.18, 130.03, 129.35, 129.16, 128.92, 128.60, 127.95, 124.68, 100.00, 70.88, 70.19, 70.08, 70.07, 69.42, 69.35, 64.01, 59.21, 57.03, 56.18, 54.17, 49.27, 49.08, 46.23, 42.16, 38.39, 38.02, 36.58, 36.17, 26.74, 26.65, 16.44, 16.39, 14.53, 13.16, 11.78. *m/z* (ESI, +ve) 1083.3 (M+H)^+^.

**(2*S,*4*R*)-1-((*S*)-15-(*tert*-butyl)-1-(1-(2-(2-((*S*)-4-(4-chlorophenyl)-2,3,9-trimethyl-6*H*-thieno[3,2-*f*][1,2,4]triazolo[4,3-*a*][1,4]diazepin-6-yl)acetamido)ethyl)-1*H*-1,2,3-triazol-4-yl)-13-oxo-2,5,8,11-tetraoxa-14-azahexadecan-16-oyl)-4-hydroxy-*N*-(4-(4-methylthiazol-5-yl)benzyl)pyrrolidine-2-carboxamide (Compound 15)**

Prepared according to the general “click ligation” method affording **Compound 15** (62 mg, 0.055 mmol, 68% yield) as pale green amorphous solid. ^1^H NMR (400 MHz, *DMSO-d*_6_) δ ppm 8.97 (1 H, s), 8.58 (1 H, t, *J*=5.9 Hz), 8.43 (1 H, t, *J*=5.6 Hz), 8.11 (1 H, s), 7.45 - 7.51 (2 H, m), 7.34 - 7.44 (7 H, m), 5.14 (1 H, d, *J*=3.5 Hz), 4.55 (1 H, d, *J*=9.6 Hz), 4.48 - 4.52 (3 H, m), 4.38 - 4.47 (4 H, m), 4.36 (2 H, br d, *J*=6.5 Hz), 4.20 - 4.31 (1 H, m), 4.08 (2 H, q, *J*=5.3 Hz), 3.95 (2 H, s), 3.63 - 3.69 (1 H, m), 3.57 - 3.63 (3 H, m), 3.45 - 3.57 (13 H, m), 3.22 (2 H, d, *J*=7.2 Hz), 3.16 (7 H, d, *J*=5.3 Hz), 2.59 (3 H, s), 2.42 - 2.45 (3 H, m), 2.40 (3 H, s), 2.03 (1 H, br d, *J*=8.2 Hz), 1.84 - 1.94 (1 H, m), 1.62 (3 H, s), 0.93 (9 H, s). ^13^C NMR (151 MHz, *DMSO-d*_6_) δ ppm 173.32, 172.23, 170.57, 169.60, 169.06, 163.58, 155.56, 151.93, 148.23, 144.32, 139.92, 139.41, 137.22, 135.67, 132.72, 131.63, 131.16, 130.69, 130.34, 130.17, 130.03, 129.35, 129.16, 128.92, 128.60, 127.93, 124.70, 70.90, 70.29, 70.26, 70.14, 70.06, 69.36, 69.35, 63.98, 59.20, 57.03, 56.16, 54.18, 49.26, 49.07, 38.38, 38.02, 36.17, 26.74, 26.64, 16.43, 16.39, 14.53, 13.15, 11.78. *m/z* (ESI, +ve) 1127.4 (M+H)^+^.

**(2*S,*4*R*)-1-((*S*)-18-(*tert*-butyl)-1-(1-(2-(2-((*S*)-4-(4-chlorophenyl)-2,3,9-trimethyl-6*H*-thieno[3,2-*f*][1,2,4]triazolo[4,3-*a*][1,4]diazepin-6-yl)acetamido)ethyl)-1*H*-1,2,3-triazol-4-yl)-16-oxo-2,5,8,11,14-pentaoxa-17-azanonadecan-19-oyl)-4-hydroxy-*N*-(4-(4-methylthiazol-5-yl)benzyl)pyrrolidine-2-carboxamide (Compound 16).**

Prepared according to the general “click ligation” method affording **Compound 16** (62 mg, 0.053 mmol, 55% yield) as off-white amorphous solid. ^1^H NMR (400 MHz, *DMSO-d*_6_) δ ppm 9.03 (1 H, s), 8.64 (1 H, t, *J*=6.0 Hz), 8.48 (1 H, t, *J*=5.8 Hz), 8.17 (1 H, s), 7.51 - 7.56 (1 H, m), 7.40 - 7.50 (4 H, m), 5.20 (1 H, d, *J*=3.5 Hz), 4.61 (1 H, d, *J*=9.6 Hz), 4.56 (1 H, s), 4.37 - 4.55 (3 H, m), 4.25 - 4.34 (1 H, m), 4.01 (1 H, s), 3.69 - 3.76 (1 H, m), 3.63 - 3.68 (1 H, m), 3.53 - 3.63 (7 H, m), 3.51 (2 H, s), 3.37 (18 H, s), 3.24 - 3.32 (1 H, m), 2.65 (2 H, s), 2.43 - 2.46 (1 H, m), 2.08 - 2.16 (1 H, m), 1.91 - 2.00 (1 H, m), 1.68 (2 H, s), 0.92 - 1.05 (5 H, m). ^13^C NMR (151 MHz, *DMSO-d*_6_) δ ppm 172.24, 170.57, 169.60, 169.06, 163.58, 155.52, 151.92, 150.32, 148.22, 144.30, 139.92, 137.22, 135.67, 132.71, 131.61, 131.16, 130.69, 130.34, 130.16, 130.03, 129.35, 129.16, 128.92, 128.60, 127.93, 124.71, 70.79, 70.29, 70.24, 70.23, 70.21, 70.05, 70.01, 69.36, 70.13, 63.98, 59.20, 57.03, 56.16, 55.38, 54.17, 49.26, 38.38, 38.02, 36.58, 36.17, 31.16, 26.75, 26.64, 16.38, 13.15. *m/z* (ESI, +ve) 1172.2 (M+H)^+^.

**(2*S*,4*R*)-1-((*S*)-2-(*tert*-Butyl)-17-((*S*)-4-(4-chlorophenyl)-2,3,9-trimethyl-6*H*-thieno[3,2-*f*][1,2,4]triazolo[4,3-*a*][1,4]diazepin-6-yl)-4,16-dioxo-6,9,12-trioxa-3,15-diazaheptadecan-1-oyl)-4-hydroxy-*N*-(4-(4-methylthiazol-5-yl)benzyl)pyrrolidine-2-carboxamide (Compound 17, MZ1).**

**Step 1: (*S*)-2-(4-(4-Chlorophenyl)-2,3,9-trimethyl-6*H*-thieno[3,2-*f*][1,2,4]triazolo[4,3-*a*][1,4]diazepin-6-yl)acetic acid.** Prepared according to Ciulli *et al*. *ACS Chem. Biol*. **2015**, *10*, 1770-1777. (*S*)-*tert*-Butyl 2-(4-(4-chlorophenyl)-2,3,9-trimethyl-6*H*-thieno[3,2-*f*][1,2,4]triazolo[4,3-*a*][1,4]diazepin-6-yl)acetate (0.26 g, 0.57 mmol, Selleckchem, Cat. No. S7110) was treated with formic acid, 96% (16 mL, 424 mmol) resulting in a bright yellow solution and allowed to stir for 16 h at r.t. The reaction mixture was treated with water (ca. 50 mL) and extracted with DCM (3 x 30 mL), dried over MgSO_4_, filtered and concentrated affording crude (*S*)-2-(4-(4-chlorophenyl)-2,3,9-trimethyl-6*H*-thieno[3,2-*f*][1,2,4]triazolo[4,3-*a*][1,4]diazepin-6-yl)acetic acid (0.21 g, 0.54 mmol, 95% yield) as light yellow film. ^1^H NMR (400 MHz, *MeOH-d^4^*) δ ppm 7.27 - 7.39 (4 H, m), 4.48 (1 H, t, *J*=7.0 Hz), 4.42 - 4.46 (1 H, m), 3.41 (2 H, d, *J*=7.2 Hz), 2.60 (3 H, s), 2.35 (3 H, s), 1.58 - 1.63 (3 H, m). *m/z* (ESI, +ve) 401.1 (M+H)^+^.

**Step 2: (2*S*,4*R*)-1-((*S*)-14-Azido-2-(*tert*-butyl)-4-oxo-6,9,12-trioxa-3-azatetradecan-1-oyl)-4-hydroxy-*N*-(4-(4-methylthiazol-5-yl)benzyl)pyrrolidine-2-carboxamide.** (2*S*,4*R*)-1-((*S*)-2-Amino-3,3-dimethylbutanoyl)-4-hydroxy-*N*-(4-(4-methylthiazol-5-yl)benzyl)pyrrolidine-2-carboxamide (0.31 g, 0.71 mmol) was treated with 11-azido-3,6,9-trioxaundecanoic acid (0.20 g, 0.85 mmol, TCI, Tokyo Chemical Industry Co), purged with argon and treated with DCM (30 mL), DIPEA (0.50 mL, 2.83 mmol) and HATU (0.40 g, 1.06 mmol) and allowed to stir at r.t. for 6 h. The reaction mixture was treated with a saturated solution of NaHCO_3_ and extracted with DCM (3 x 50 mL), dried over Na_2_SO_4_, filtered and concentrated. Purification of the crude residue by column chromatography on silica gel using a gradient of 0-10% MeOH in DCM affording (2*S*,4*R*)-1-((*S*)-14-azido-2-(*tert*-butyl)-4-oxo-6,9,12-trioxa-3-azatetradecan-1-oyl)-4-hydroxy-*N*-(4-(4-methylthiazol-5-yl)benzyl)pyrrolidine-2-carboxamide (0.36 g, 0.56 mmol, 79% yield) as clear, colorless tar. ^1^H NMR (400 MHz, *CDCl_3_*) δ ppm 8.68 (1 H, s), 7.31 - 7.41 (5 H, m), 4.75 (1 H, t, *J*=7.9 Hz), 4.51 - 4.61 (2 H, m), 4.48 (1 H, d, *J*=8.4 Hz), 4.34 (1 H, dd, *J*=14.9, 5.3 Hz), 4.10 - 4.17 (1 H, m), 3.95 - 4.07 (2 H, m), 3.63 - 3.71 9 H, m), 3.60 (1 H, dd, *J*=11.3, 3.7 Hz), 3.37 (2 H, t, *J*=5.1 Hz), 2.75 - 3.07 (1 H, m), 2.55 - 2.64 (1 H, m), 2.52 (3 H, s), 2.06 - 2.17 (1 H, m), 1.38 - 1.53 (2 H, m), 0.95 (9 H, s). *m/z* (ESI, +ve) 646.3 (M+H)^+^.

**Step 3: (2*S*,4*R*)-1-((*S*)-14-Amino-2-(*tert*-butyl)-4-oxo-6,9,12-trioxa-3-azatetradecan-1-oyl)-4-hydroxy-*N*-(4-(4-methylthiazol-5-yl)benzyl)pyrrolidine-2-carboxamide.** (2*S*,4*R*)-1-((*S*)-14-Azido-2-(*tert*-butyl)-4-oxo-6,9,12-trioxa-3-azatetradecan-1-oyl)-4-hydroxy-*N*-(4-(4-methylthiazol-5-yl)benzyl)pyrrolidine-2-carboxamide (0.34 g, 0.52 mmol) was dissolved in MeOH (10 mL) and treated with palladium, 10 wt.% (dry basis) on activated carbon, wet, degussa type e101 (55 mg, 0.052 mmol) in a 75 mL reactor under nitrogen. The reaction vessel was then sealed, purged with hydrogen (6x) and allowed to stir at r.t. under 15 psi hydrogen for 3 h. The reaction mixture was filtered through an 0.45 micro acrodisc and concentrated to dryness affording crude (2*S*,4*R*)-1-((*S*)-14-amino-2-(*tert*-butyl)-4-oxo-6,9,12-trioxa-3-azatetradecan-1-oyl)-4-hydroxy-*N*-(4-(4-methylthiazol-5-yl)benzyl)pyrrolidine-2-carboxamide (0.32 g, 0.51 mmol, 98 % yield) as clear, colorless film. ^1^H NMR (400 MHz, *MeOH-d_4_*) δ ppm 7.46 (4 H, q, *J*=8.3 Hz), 4.72 (1 H, s), 4.55 - 4.62 (1 H, m), 4.49 - 4.55 (2 H, m), 4.34 - 4.43 (1 H, m), 4.05 - 4.10 (2 H, m), 3.86 - 3.93 (1 H, m), 3.79 - 3.86 (1 H, m), 3.60 - 3.77 (9 H, m), 3.57 (2 H, t, *J*=5.3 Hz), 3.37 (2 H, s), 3.34 - 3.36 (2 H, m), 2.87 (2 H, t, *J*=5.2 Hz), 2.48 - 2.52 (3 H, m), 2.25 (1 H, dd, *J*=13.1, 7.4 Hz), 2.06 - 2.15 (1 H, m), 1.07 (8 H, s), 1.04 (1 H, s). *m/z* (ESI, +ve) 620.2 (M+H)^+^.

**Step 4: (2*S*,4*R*)-1-((*S*)-2-(*tert*-Butyl)-17-((*S*)-4-(4-chlorophenyl)-2,3,9-trimethyl-6*H*-thieno[3,2-*f*][1,2,4]triazolo[4,3-*a*][1,4]diazepin-6-yl)-4,16-dioxo-6,9,12-trioxa-3,15-diazaheptadecan-1-oyl)-4-hydroxy-*N*-(4-(4-methylthiazol-5-yl)benzyl)pyrrolidine-2-carboxamide (Compound 17).** (*S*)-2-(4-(4-Chlorophenyl)-2,3,9-trimethyl-6*H*-thieno[3,2-*f*][1,2,4]triazolo[4,3-*a*][1,4]diazepin-6-yl)acetic acid (0.10 g, 0.25 mmol) and (2*S*,4*R*)-1-((*S*)-14-amino-2-(*tert*-butyl)-4-oxo-6,9,12-trioxa-3-azatetradecan-1-oyl)-4-hydroxy-*N*-(4-(4-methylthiazol-5-yl)benzyl)pyrrolidine-2-carboxamide (0.17 g, 0.27 mmol) were treated with DCM (6 mL) followed by DIPEA (0.17 mL, 1.00 mmol) and HATU (0.14 g, 0.37 mmol) and the solution was allowed to stir at r.t. for 4 h. The reaction mixture was concentrated to dryness on the rotovap and the crude residue dissolved in DMSO (6 mL) and purified on a Gilson (Gemini Phenomenex; 30 x 150 mm, 5 u, 10-90% 0.1%TFA/CH_3_CN in 0.1%TFA/water), concentrated affording a yellow, clear tar. The tar was taken up in 30% MeOH/DCM and passed through an Agilent PL-HCO3MP SPE cartridge and concentrated in the genevac affording (2*S*,4*R*)-1-((*S*)-2-(*tert*-butyl)-17-((*S*)-4-(4-chlorophenyl)-2,3,9-trimethyl-6*H*-thieno[3,2-*f*][1,2,4]triazolo[4,3-*a*][1,4]diazepin-6-yl)-4,16-dioxo-6,9,12-trioxa-3,15-diazaheptadecan-1-oyl)-4-hydroxy-*N*-(4-(4-methylthiazol-5-yl)benzyl)pyrrolidine-2-carboxamide (**Compound 17**, 0.15 g, 0.15 mmol, 60% yield) as light yellow tar. ^1^H NMR (400 MHz, *MeOH-d_4_*) δ ppm 8.85 - 8.91 (1 H, m), 7.80 (1 H, d, *J*=8.6 Hz), 7.73 (1 H, d, *J*=8.6 Hz), 7.56 (1 H, d, *J*=8.4 Hz), 7.51 (1 H, d, *J*=8.6 Hz), 7.37 - 7.50 (9 H, m), 4.72 (1 H, s), 4.56 - 4.67 (2 H, m), 4.47 - 4.56 (2 H, m), 4.32 - 4.41 (1 H, m), 4.08 (2 H, d, *J*=3.7 Hz), 3.86 - 3.93 (1 H, m), 3.78 - 3.86 (1 H, m), 3.71 - 3.76 (5 H, m), 3.63 - 3.71 (5 H, m), 3.61 (2 H, t, *J*=5.7 Hz), 3.42 - 3.53 (4 H, m), 3.37 (9 H, s), 2.70 (3 H, s), 2.43 - 2.51 (7 H, m), 2.19 - 2.31 (2 H, m), 2.04 - 2.16 (1 H, m), 1.93 - 2.00 (1 H, m), 1.71 (3 H, s), 1.06 (9 H, s), 1.04 (1 H, s). *m/z* (ESI, +ve) 1024.4 (M+Na)^+^.

**Supplementary Note 2:**

**Mathematical Framework for Describing** **SPR-derived Binding Parameters of PROTAC-Mediated Ternary Complex Formation**

The total ligase concentration is sum of the free ligase binding sites [*L*] and total bound ligase [*LPT*] at equilibrium as follows:

[*L*]_t_ = [*L*] + [*LPT*] (A1)

Substituting eq 2 after rearrangement into eq A1 yields:

$\frac{[LPT]}{{[L]}_{t}}=\frac{[TP]}{{[TP]+K}_{LPT}}$ (A2)

Here, the binary complex [*TP*] comprising *T* and *P* is defined in terms of the equilibrium dissociation constant (*K_TP_*) as:

$K_{TP}=\frac{\left[ T \right][P]}{[TP]}$ (A3)

The total PROTAC concentration is the sum of the free PROTAC [*P*] and total bound target [*TP*] as:

[*P*]_t_ = [*P*] + [*TP*] (A4)

Similarly, total target concentration is the sum of the free target [*T*] and total bound target [*TP*] as:

[*T*]_t_ = [*T*] + [*TP*] (A5)

Substituting eq A3 after rearrangement into eq A4 yields:

$\frac{[TP]}{{[P]}_{t}}=\frac{[T]}{{[T]+K}_{TP}}$ (A6)

Eq A6 can be fitted to a hyperbolic binding curve (Figure A1) by plotting target concentrations as a multiplier of binary binding affinity (*K_TP_*) against the fraction of binary complex. Figure 1 suggests that at [*T*]*_t_* ≈ 25×*K_TP_*, saturation of PROTAC molecules (~96%) bound to the target [*TP*] can be achieved. [*T*]_t_ ≅ [*T*], when total target concentration [*T*]_t_ >>[*TP*] to ensure the binary complex in SPR analysis.


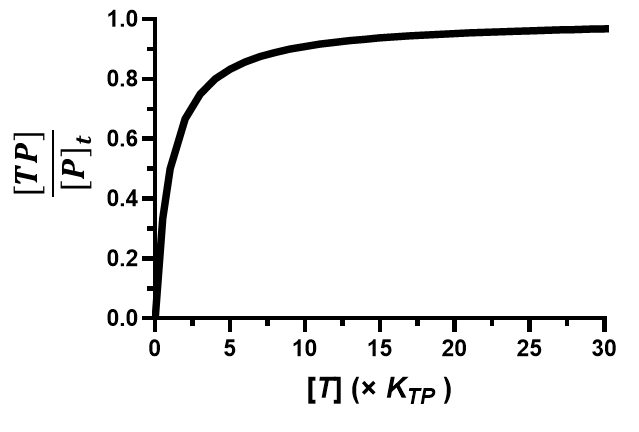


**Supplementary Figure 1.** Binding curve of varying concentrations of target (as a multiplier of binary binding affinity, *K_TP_*) to fraction of binary complex.

[*P*]_t_ ≅ [*TP*], when total target concentration [*T*]_t_ >>[*P*]_t_ to ensure the binary complex in SPR analysis, therefore, eq A2 can be written as:

$\frac{[LPT]}{{[L]}_{t}}=\frac{\left[ P \right]_{t}}{{\left[ P \right]_{t}+K}_{LPT}}$ (A7)

The binary interaction involving equilibrium dissociation constant (*K_LP_*) of *P* and surface-bound ligase (*L*) is defined as:

$K_{LP}=\frac{\left[ L \right][P]}{[LP]}$ (A8)

The binary complex (*TP*) in eq A3 is substituted into eq 2 to obtain eq A9 as follows:

$K_{LPT}K_{TP}=\frac{\left[ L \right]\left[ P \right][T]}{[LPT]}$ (A9)

In the SPR binding assay, cooperativity (*α*), is defined as the ratio of PROTAC/ligase binary binding affinity (*K_LP_*) to ternary complex binding affinity (*K_LPT_*).

$\alpha=\frac{K_{LP}}{K_{LPT}}$ (A10)

Eq A9 can be rewritten by incorporating cooperativity from eq A10 as follows:

$\frac{K_{LP}K_{TP}}{\alpha}=\frac{\left[ L \right]\left[ P \right][T]}{[LPT]}$ (A11)

As discussed by Douglass *et al*.,^1^ total concentration of each species at the ternary complex equilibria can be expressed according to the conservation of mass:

$\left[ L \right]_{t}=\left[ L \right]+\left[ LP \right]+\left[ LPT \right]$ (A12)

$\left[ T \right]_{t}=\left[ T \right]+\left[ TP \right]+\left[ LPT \right]$ (A13)

Rearranging eq A12 and eq A13 after substituting eq A8 and eq A3, respectively as follows:

$\left[ L \right]=\frac{K_{LP}(\left[ L \right]_{t}-\left[ LPT \right])}{K_{LP}+[P]}$ (A14)

$\left[ T \right]=\frac{K_{TP}(\left[ T \right]_{t}-\left[ LPT \right])}{K_{TP}+[P]}$ (A15)

Substituting eq A14 and eq A15 into eq A11:

$\left[ LPT \right]=\frac{\alpha\left[ P \right](\left[ L \right]_{t}-\left[ LPT \right])(\left[ T \right]_{t}-\left[ LPT \right])}{(K_{LP}+\left[ P \right])(K_{TP}+\left[ P \right])}$ (A16)

Rearrangement of eq A16 yields a quadratic equation:

$\left[ LPT \right]^{2}-\left[ LPT \right]\left( \left[ L \right]_{t}+\left[ T \right]_{t}+\frac{\left( K_{LP}+\left[ P \right] \right)\left( K_{TP}+\left[ P \right] \right)}{\alpha\left[ P \right]} \right)+\left[ L \right]_{t}\left[ T \right]_{t}=0$ (A17)

Eq A17 is algebraically unsolvable as discussed earlier ^1, 2^. However, it can be solved for ${[LPT]}_{max}$, the concentration of [*P*] at which it is maximum ($\frac{d[LPT]}{d[P]}=0)$ by taking the differentiation of eq A17 with respect to [*P*] as follows: $2\left[ LPT \right]\frac{d[LPT]}{d[P]} -\frac{d\left[ LPT \right]}{d\left[ P \right]}\left( \left[ L \right]_{t}+\left[ T \right]_{t}+\frac{\left( K_{LP}+\left[ P \right] \right)\left( K_{TP}+\left[ P \right] \right)}{\alpha\left[ P \right]} \right)-\left[ LPT \right]\frac{d\left[ \frac{\left( K_{LP}+\left[ P \right] \right)\left( K_{TP}+\left[ P \right] \right)}{\alpha\left[ P \right]} \right]}{d\left[ P \right]}=0$ (A18)

At ${[LPT]}_{max}$, $\frac{d[LPT]}{d[P]}=0$, thus A16 can be simplified by getting rid of 1^st^ and 2^nd^ terms as:

$\frac{d\left[ \frac{\left( K_{LP}+\left[ P \right] \right)\left( K_{TP}+\left[ P \right] \right)}{\alpha\left[ P \right]} \right]}{d\left[ P \right]}=0$ (A19)

Solving differentiation of A19 yielded:

$\left[ P \right]^{2}- K_{LP}K_{TP}=0$ (A20)

Eq A20 can be further rearranged:

$\left[ P \right]=\sqrt{K_{LP}K_{TP}}$ (A21)

Eq A17 can be rewritten for ${[LPT]}_{max}$ by substituting $\left[ P \right]=\sqrt{K_{LP}K_{TP}}$ as follows:

${[LPT]}_{max}^{2}-{[LPT]}_{max}\left( \left[ L \right]_{t}+\left[ T \right]_{t}+\frac{{(\sqrt{K_{LP}}+\sqrt{K_{TP}})}^{2}}{\alpha} \right)+\left[ L \right]_{t}\left[ T \right]_{t} =0$ (A22)

In SPR experiments, $\left[ L \right]_{t}$ is equivalent to total surface-immobilized ligase and $\left[ T \right]_{t}$is the total concentration of target in the solution. In the binding assay (Figure 1C), $\left[ T \right]_{t}$>> $\left[ P \right]_{t}$to ensure that all PROTAC [*P*] remains as a binary complex $\left[ TP \right]$. Surface-immobilized $\left[ L \right]_{t}$ can be estimated by the surface capture/immobilization SPR response. For example, 400 RU capturing/immobilizing of ligase would be equivalent to a surface density of ~0.01 pmol/mm^2^.^3^ Conversely, the target molecule mass flux at the SPR chip surface can be approximated as^4^: *D**[*T*]/𝛅, where *D* is the diffusivity of the target molecule, [*T*]_t_ is the total target concentration and 𝛅 is the diffusion layer thickness (Figure 2B). For a flow rate of 80 µL/min, 𝛅 would be in the order of 1 µm,^5^ diffusivity of both targets (BRD4 or SMARCA2) were measured as 10^-6^ cm^2^/s,^6^ and target protein concentration [*T*]_t_, 2.5 µM, was always kept much higher than [*P*] to ensure binary complex between PROTAC and target. The target mass flux for 60 s onto the SPR surface would be ~15 pmol/mm^2^, which would make the total ligase molecules onto the SPR chip (~0.01 pmol/mm^2^) much smaller than the total target molecules available in the SPR flow cell, therefore under SPR experimental conditions $\left[ T \right]_{t}$>> $\left[ L \right]_{t.}$

Eq A22 is divided by ${[T]}_{t}$ as follows:

${[LPT]}_{max}^{2}/{[T]}_{t}-{[LPT]}_{max}\left[ L \right]_{t}/\left[ T \right]_{t}-{[LPT]}_{max}\left( 1+\frac{{(\sqrt{K_{LP}}+\sqrt{K_{TP}})}^{2}}{\alpha{[T]}_{t}} \right)+\left[ L \right]_{t} =0$ (A23)

As a first order approximation, the first and second terms of eq A23 (${\left[ T \right]_{t}>> \left[ L \right]_{t}or [LPT]}_{max})$,

can cancel each other out, which transforms it into:

$\frac{{[LPT]}_{max}}{\left[ L \right]_{t}}\cong\frac{\alpha}{\left( \alpha+\frac{{(\sqrt{K_{LP}}+\sqrt{K_{TP}})}^{2}}{\left[ T \right]_{t}} \right)}$ (A24)

**Supplementary Figure 2**: Correlation between PROTAC’s area under the dose–response curve (AUC) and cooperativity factor (*α*) for SMARCA2 **(A)** and BRD4^BD2^ **(B)**. AUC show negative correlation (***P* = 0.008) with the cooperativity for SMARCA2 PROTACs. Similarly, AUC for BRD4 PROTACs demonstrate negative correlation (**P* = 0.012) with the cooperativity. Pearson correlation coefficient (r) is used for correlation analysis and two-tailed test for significance.

 **(A) (B)**

**Supplementary Table 1.** SPR-measured ternary complex binding parameters of SMARCA2 degraders.


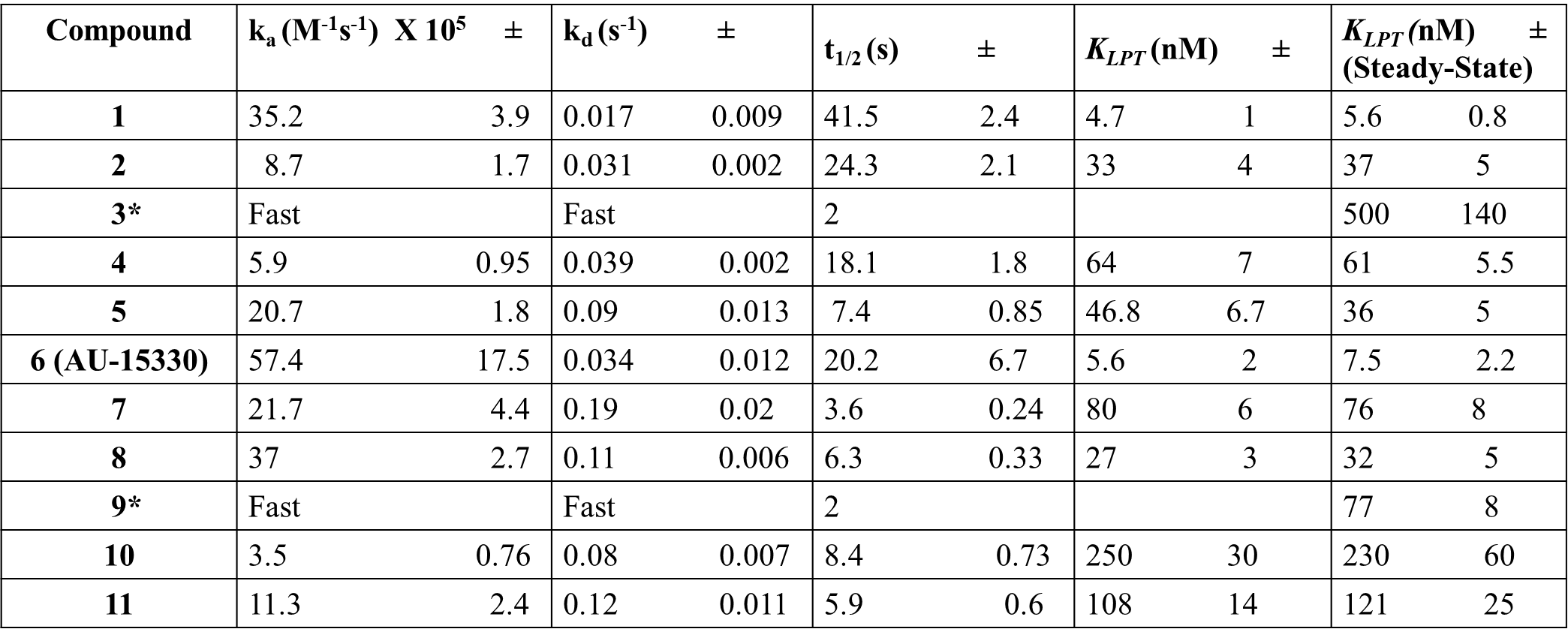


SPR analysis was performed using 1:1 Langmuir interaction or steady-state affinity models. Error is SEMs for *N=3* or SD for *N=2*. Chi^2^ and uniqueness (U) values were used to determine the quality of fitted parameters and confidence in the results. Steady-state binding affinity for compounds **3** and **10** was calculated using steady-state affinity model with constant R_max_ (90 RU, based on compound **1**); * ternary complex half-life of 2 s was assumed for compounds **3** and **9** with fast-off binding kinetics.

**Supplementary Figure 3.** Representative SPR sensorgrams of preformed binary complex comprising PROTACs and SMARCA2 binding to VBC-functionalized surfaces.


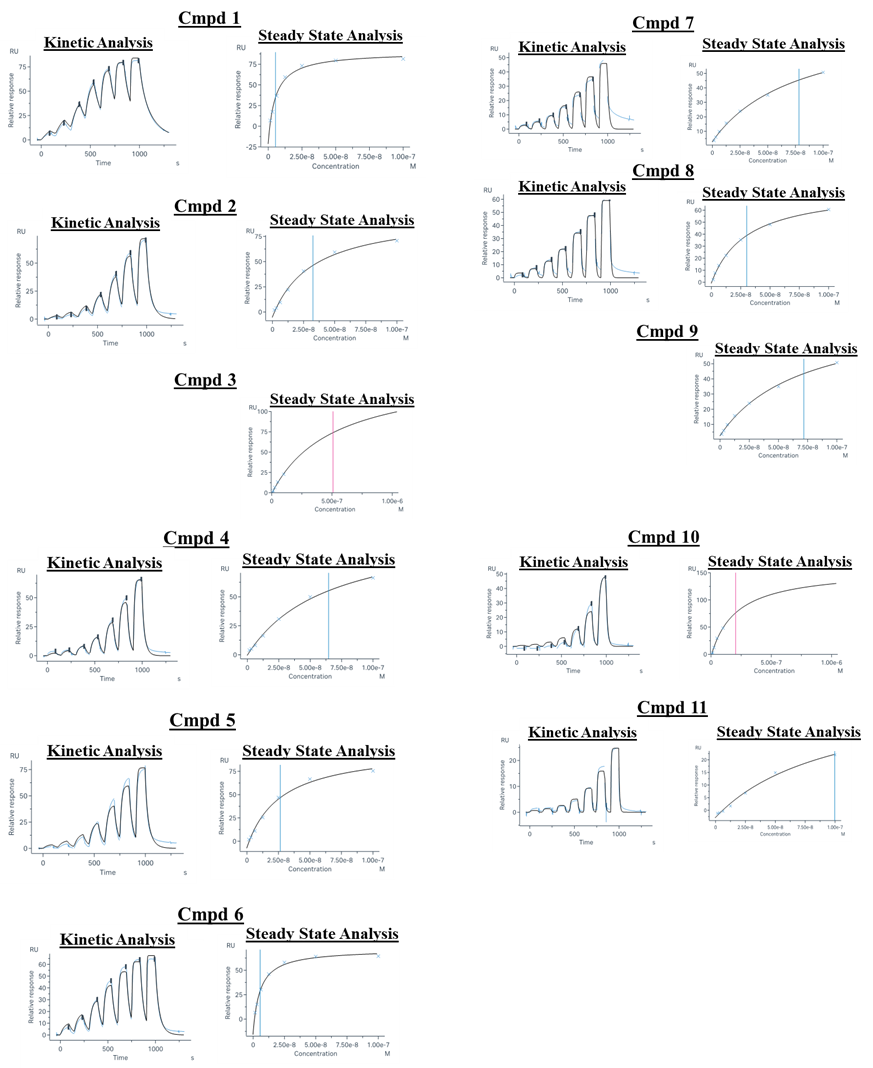


**Supplementary Figure 4.** Representative SPR sensorgrams of SMARCA2 PROTACs binding to VBC‑functionalized surfaces. SPR analysis was performed using 1:1 Langmuir interaction model.


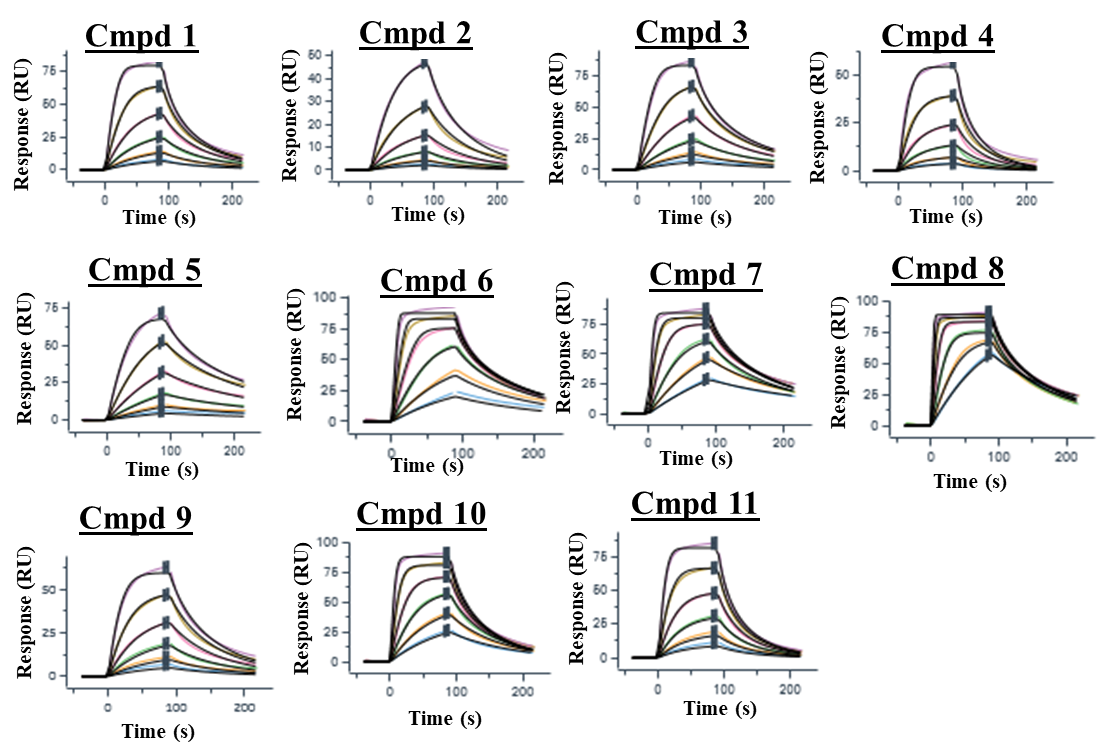


**Supplementary Table 2**. SPR-based binary affinity data for PROTACs and SMARCA2 interaction.

| Target | PROTAC | *K_TP_* (nM) |
| --- | --- | --- |
| SMARCA2^BD^ | **1** | 95 ± 25.5 |
|  | **2** | 196 ± 23 |
|  | **3** | 225 ± 24 |
|  | **4** | 232 ± 35 |
|  | **5** | 206 ± 34 |
|  | **6 (AU-15330)** | 14.2 ± 1.6 |
|  | **7** | 46 ± 8.5 |
|  | **8** | 49 ± 12 |
|  | **9** | 294 ± 33 |
|  | **10** | 745 ± 134 |
|  | **11** | 302 ± 80 |

SPR analysis was performed using 1:1 Langmuir binding model. Error is

SEMs for *N=3* or SD for *N=2*.

**Supplementary Figure 5.** Representative SPR sensorgrams of PROTACs binding to SMARCA2‑functionalized surfaces. SPR analysis was performed using 1:1 Langmuir interaction model.


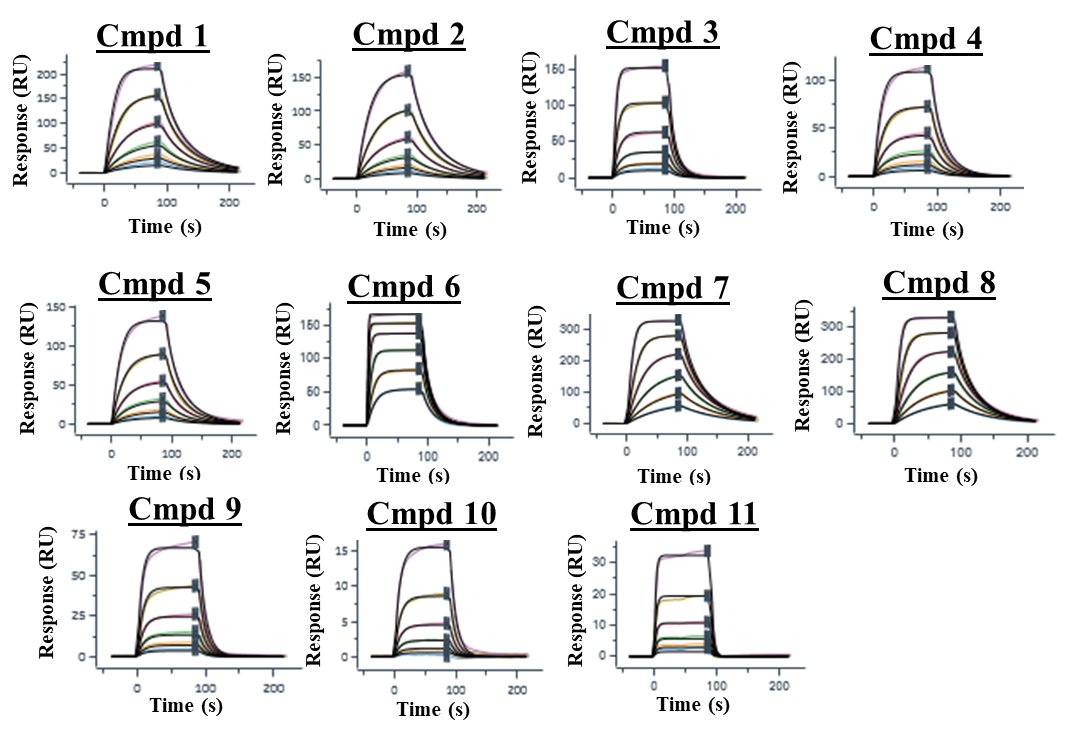


**Supplementary Figure 6.** SPR sensorgrams of PROTAC-mediated ternary complex formation between VBC and BRD4 bromodomains (BD1/BD2) for **17** (MZ1).

**Supplementary Table 3.** SPR-measured ternary complex binding parameters of BRD4 degraders.

**
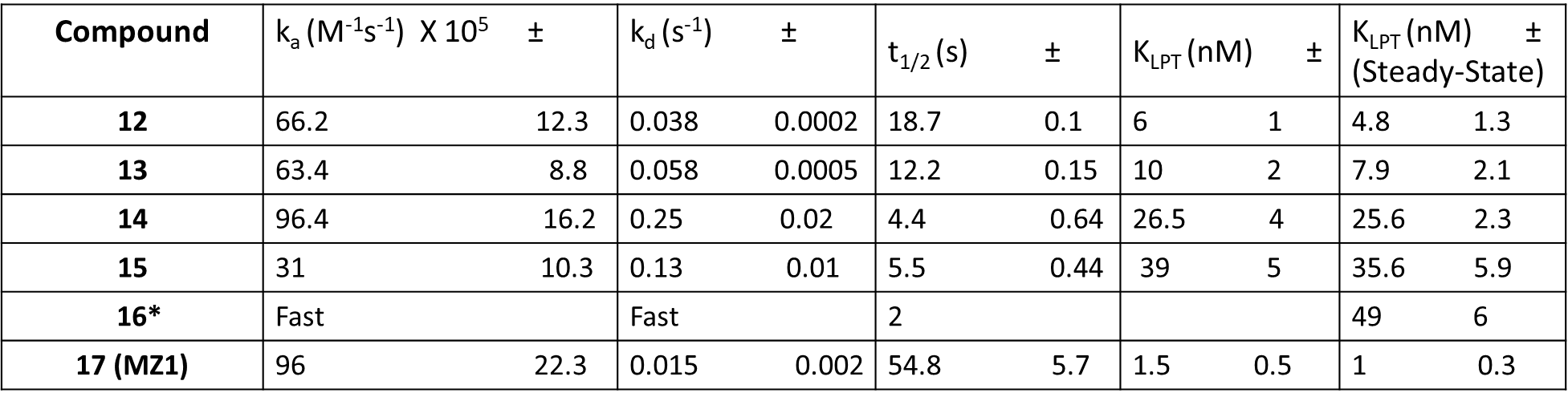
**SPR analysis was performed using 1:1 Langmuir interaction or steady-state affinity models. Error is SEMs for *N=3* or SD for *N=2*. Chi^2^ and uniqueness (U) values were used to determine the quality of fitted parameters and confidence in the results; * ternary complex half-life of 2 s was assumed for compound **16** with fast-off binding kinetics.

**Supplementary Figure 7.** Representative SPR sensorgrams of preformed binary complex comprising PROTACs and BRD4^BD2^ binding to VBC-functionalized surfaces.


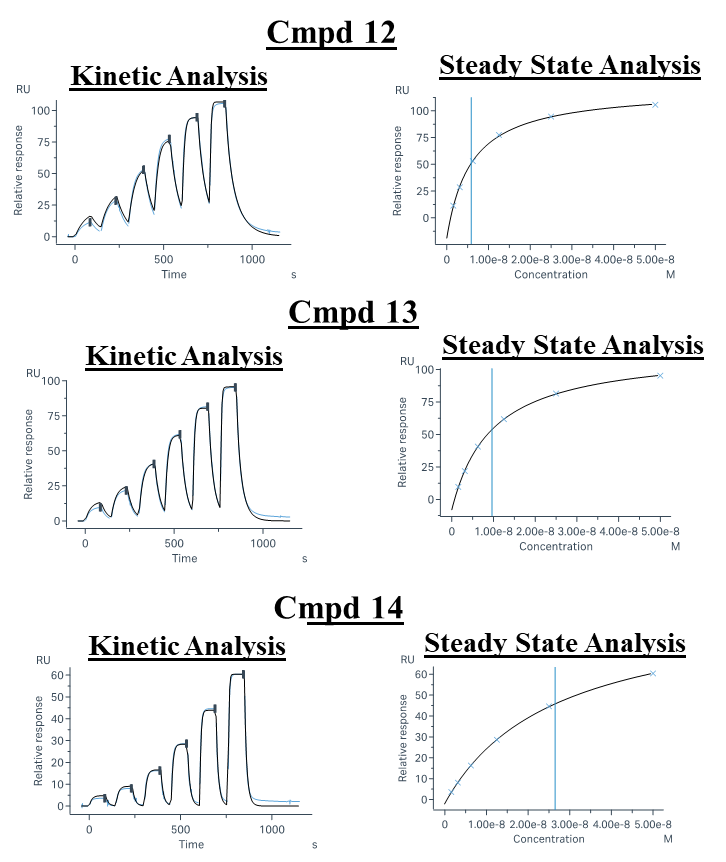

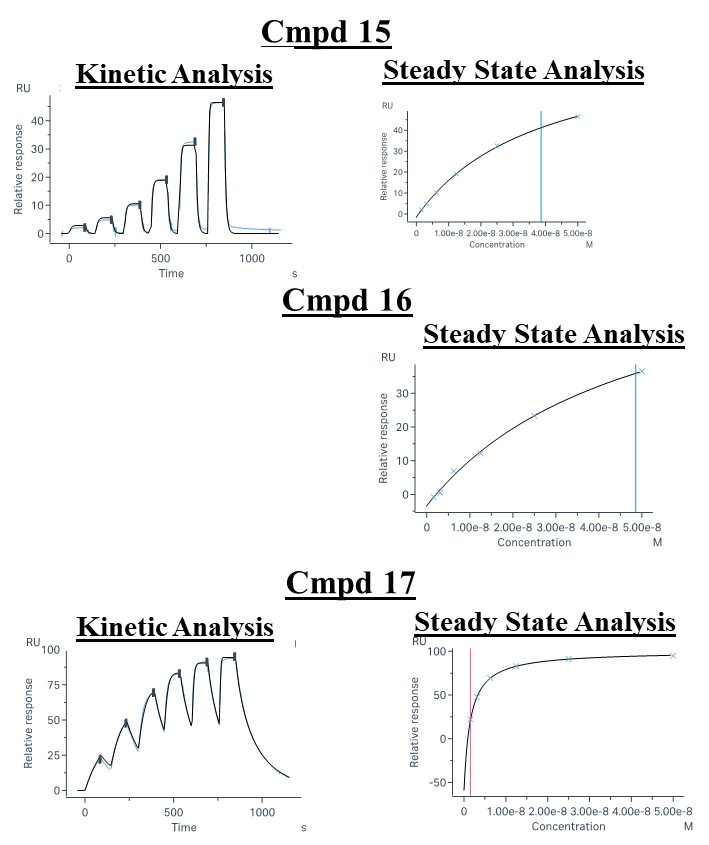


**Supplementary Figure 8.** Representative SPR sensorgrams of BRD4 PROTACs binding to VBC‑functionalized surfaces. SPR analysis was performed using 1:1 Langmuir interaction model.

**
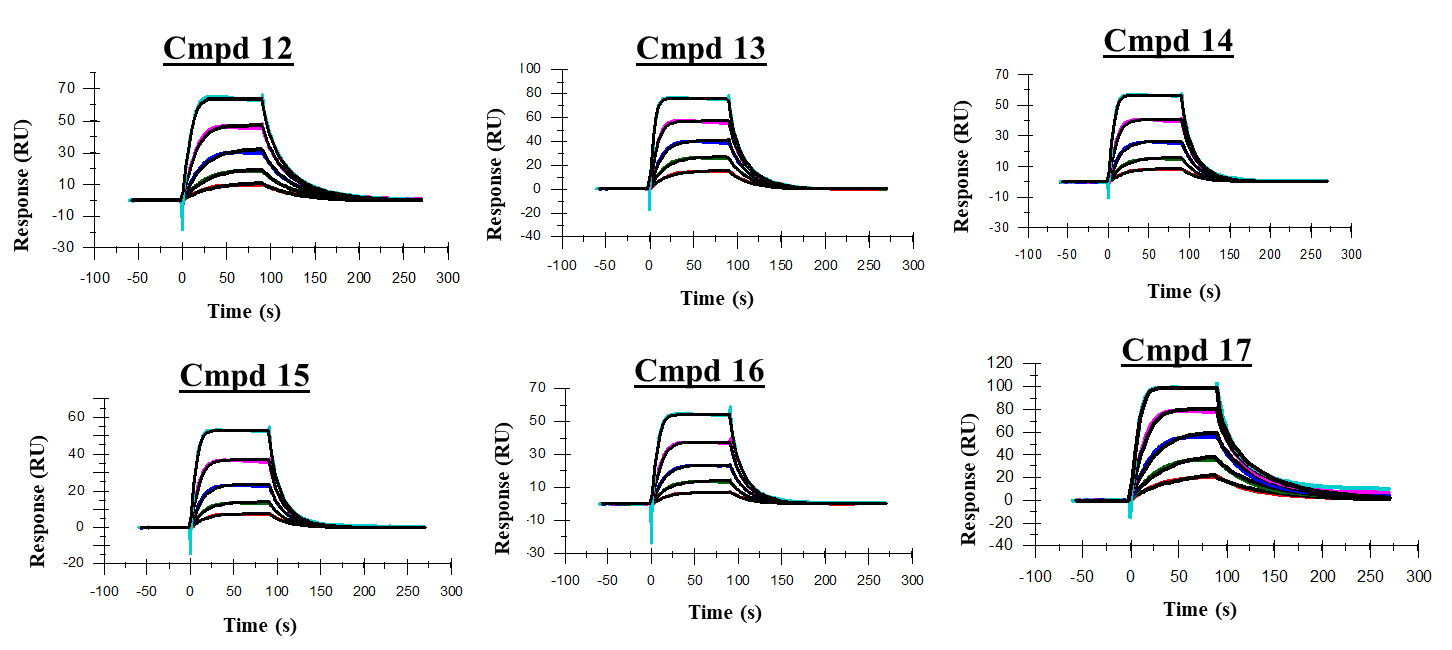
**

**Supplementary Table 4**. SPR-based binary affinity data for PROTACs and BRD4^BD2^ interaction.

| Target | PROTAC | *K_TP_* (nM) |
| --- | --- | --- |
| BRD4^BD2^ | **12** | 20.5 ± 1.2 |
|  | **13** | 11.7 ± 0.9 |
|  | **14** | 9.6 ± 0.9 |
|  | **15** | 16.2 ± 2.6 |
|  | **16** | 15.8 ± 2 |
|  | **17** (MZ1) | 24.4 ± 2 |

SPR analysis was performed using 1:1 Langmuir binding model. Error is SD for *N=*2.

**Supplementary Figure 9.** Representative SPR sensorgrams of PROTACs binding to BRD4^BD2^-functionalized surfaces.


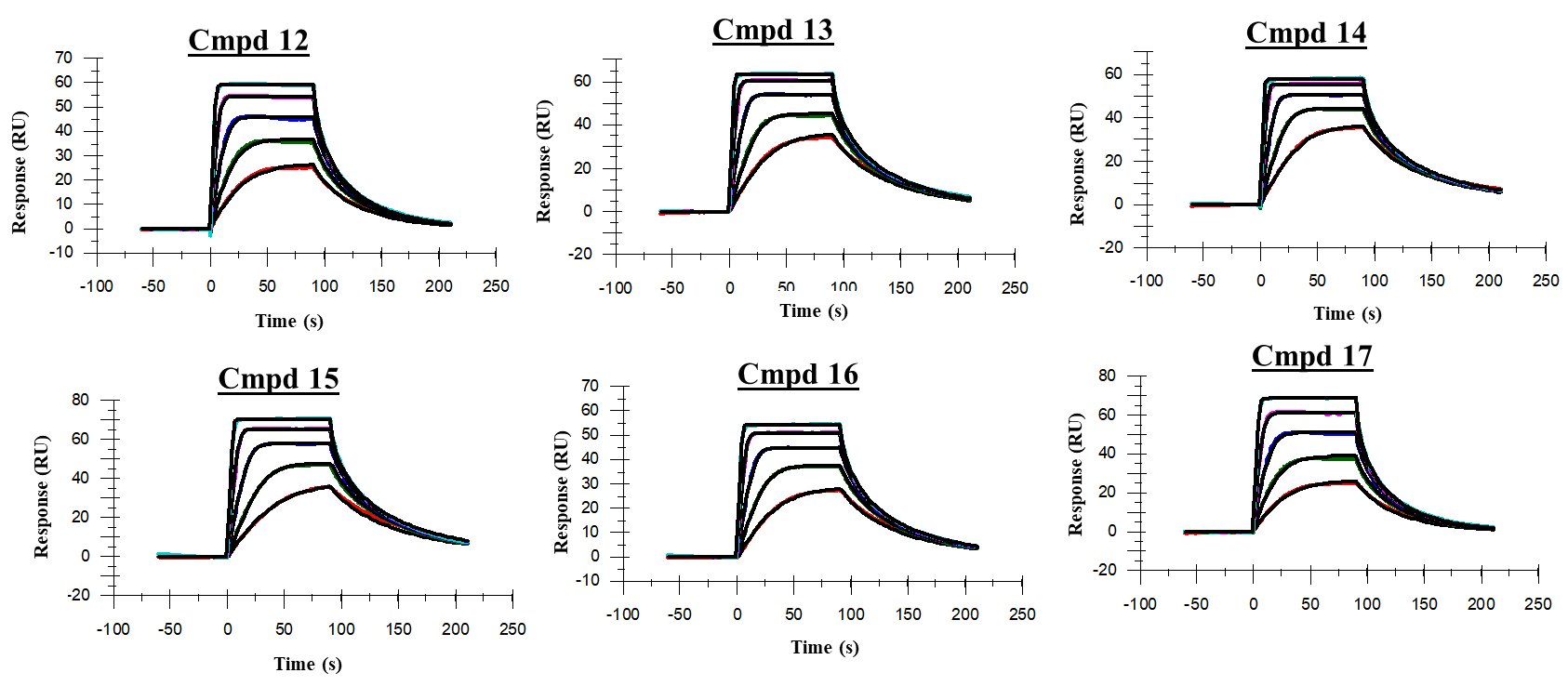


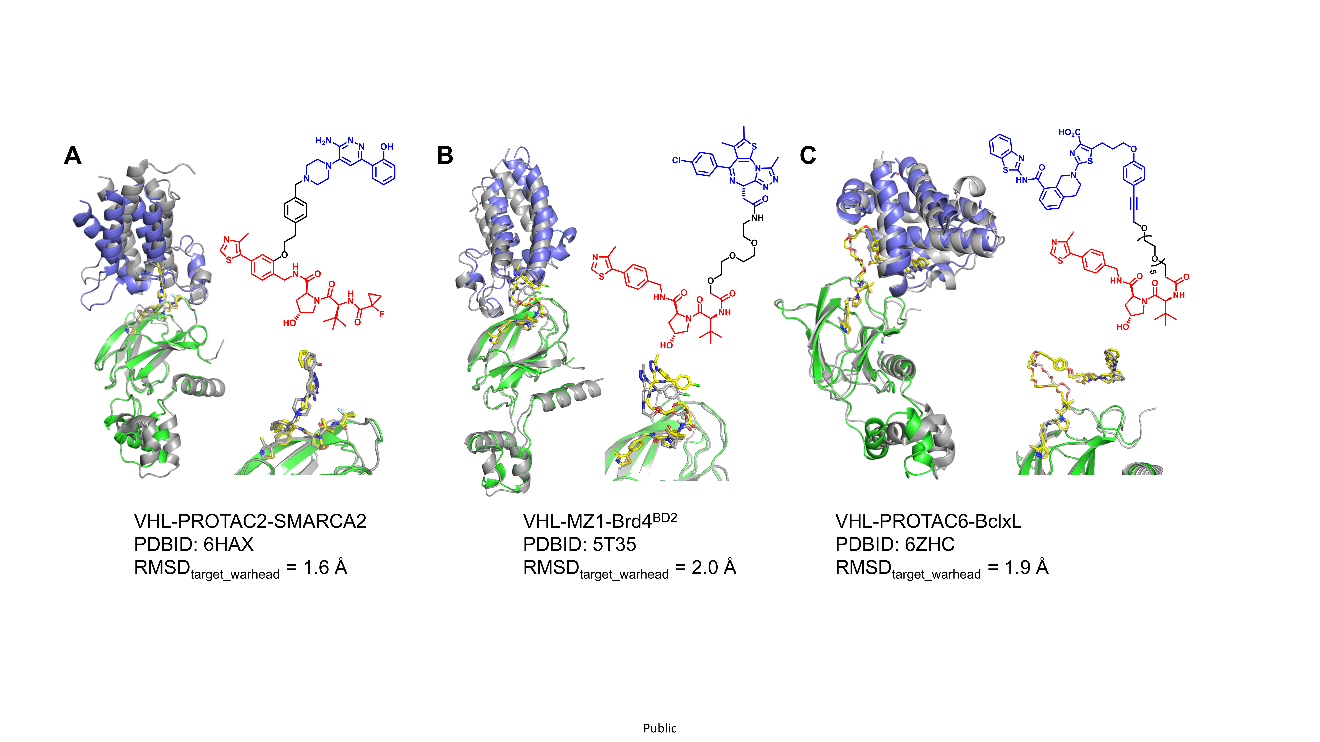


**Supplementary Figure 10.** Comparison of the top model (slate/yellow/green) and the crystal structures (light grey) in three known PROTAC induced complexes with target warhead RMSD shown on the bottom.


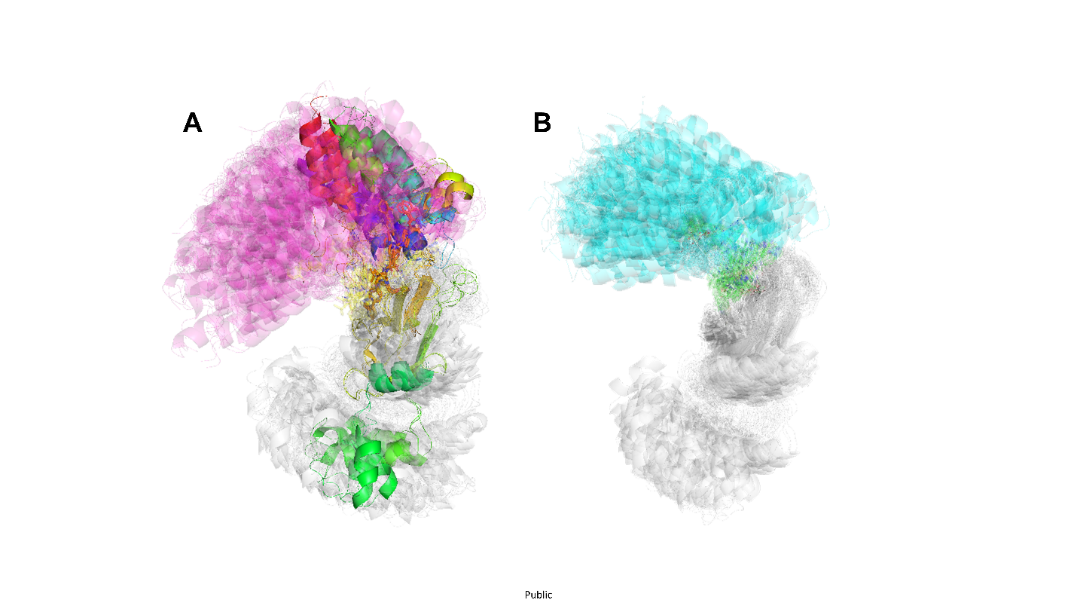


**Supplementary Figure 11.** Comparison of the MD simulation snapshots for (A) the SMARCA2^BD^-Cmpd **1**-VBC model and (B) the SMARCA4^BD^-Cmpd **1**-VBC crystal structure. The top two models (*rainbow*) from the ternary structure modeling workflow are well within the structural ensemble from MD simulations of SMARCA2-Cmpd **1**-VHL. The structures are aligned using VHL.

**Supplementary Note 3:**

**Ternary Complex Crystallization**

Crystallization of PROTAC-mediated ternary complex was performed using sitting drop vapor diffusion method on 96 well trays. VBC: SMARCA2^BD^ were mixed as a 1:1 stoichiometric ratio in 10 mM HEPES (7.5), 150 mM NaCl, 0.5 mM TCEP and concentrated to approximately 10 mg/ml. Compound **11** (PROTAC 2) and compound **1** was then added to mixture in 1:1 stoichiometric ratio and incubated for 20 minutes in ice. Drops of the ternary complex were mixed 1:1 and 1:2 in crystallization buffer using a Mosquito® robot (SPT Labtech). With compound **11**, crystals appeared within 7 days in reservoir solution containing 20% PEG 3350, 0.2 M sodium chloride.

Extensive screening efforts with compound **1** with SMARCA2^BD^ and VBC failed to generate any hits. HT screening efforts with compound **1** with SMARCA4^BD^ and VBC resulted in hits in reservoir solution containing 0.1 M BIS-TRIS pH 6.5, 25% of polyethylene glycol 300. These crystals were then optimized for crystal growth using streak seeding method. Crystals were flash frozen in reservoir solution supplemented with glycerol as cryo-protectant. All data sets were collected on a Pilatus3 6M silicon pixel detector at the Advanced Light Source Beamline 5.0.2 at wavelength 1.00000 Å and temperature 100 K. The data were integrated and scaled using HKL2000. The structures were solved by molecular replacement using Phaser from the CCP4 program suite with apo-SMARCA2 and apo-VBC as a search model. The structures were refined using Phenix. The structure of VBC: Cmpd **11**: SMARCA2^BD^ refined to 2.7 Å resolution with R-factor of 21% and R_free_ of 26%. The structure of VBC: Cmpd **1**: SMARCA4^BD^ refined to 3.7 Å resolution with R-factor of 23.4% and R_free_ of 32.5%.


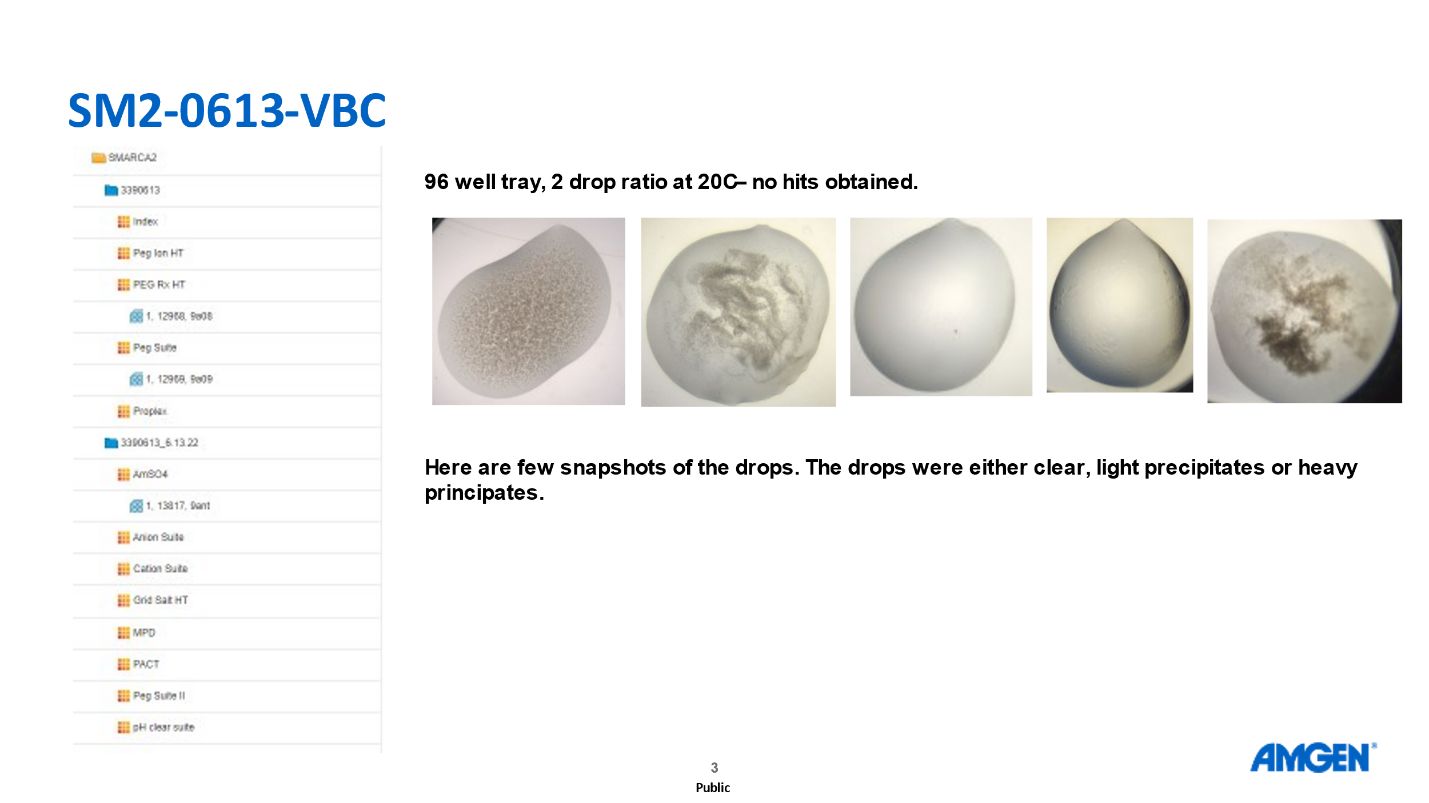


Co-crystallization of compound **1** with SMARCA2/VBC resulted into either clear or precipitated samples.

**
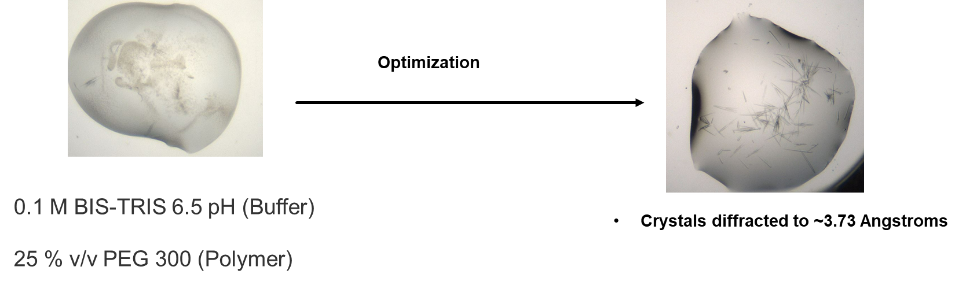
**

Co-crystallization of compound **1** with SMARCA4/VBC resulted in diffraction with average 3.7 Å resolution.

**Supplementary Table 5.** Crystallographic data collection and refinement statistics for SMARCA2^BD^/Cmpd11/VBC and SMARCA4^BD^/Cmpd1/VBC reported in the manuscript.


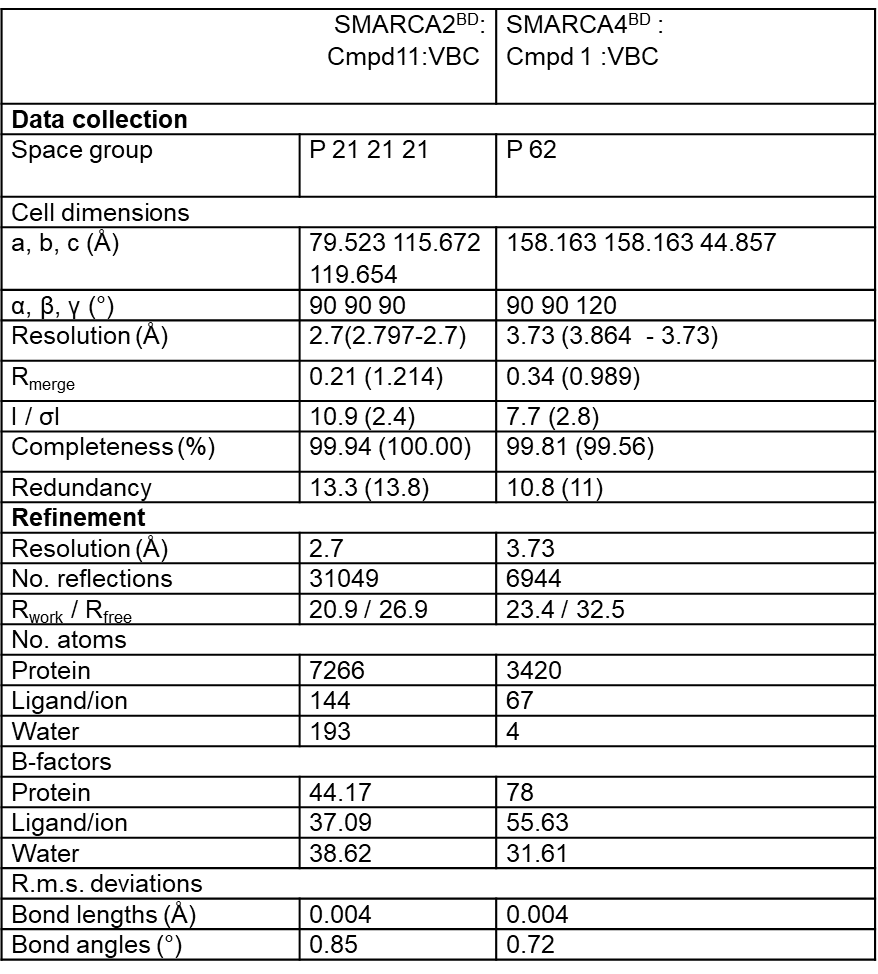


*****Statistics for the highest-resolution shell are shown in parentheses.


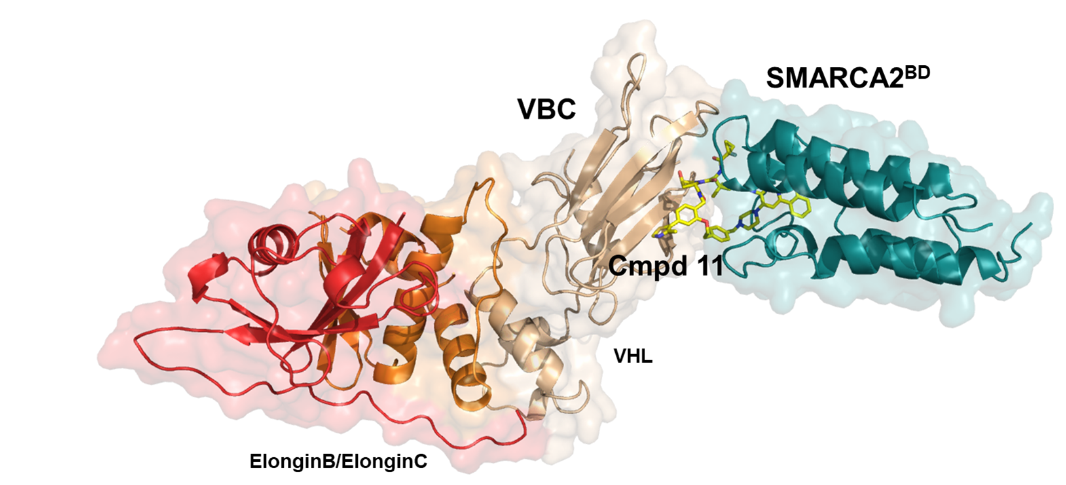


**Supplementary Figure 12.** Crystal structure of VBC-Cmpd **11**-SMARCA2 at 2.7Å.

**Additional H-bond interaction observed with Cmpd 11:**

- Fluoro makes H-bond with backbone oxygen of **Phe1463** (3.1 Å)
- Backbone oxygen of SMARCA2 **Asn1464**  makes H-bond with **Tyr112** of VHL (2.6 Å)
- Backbone oxygen atoms **Phe1463** and **Thr1462** of SMARCA2 makes H-bond with sidechain of **Arg69**  of VHL (2.8 Å, 2.4 Å).
- Backbone oxygen of SMARCA2 **Gly1467** makes H-bond with sidechain **Gln73** of VHL (2.5 Å).


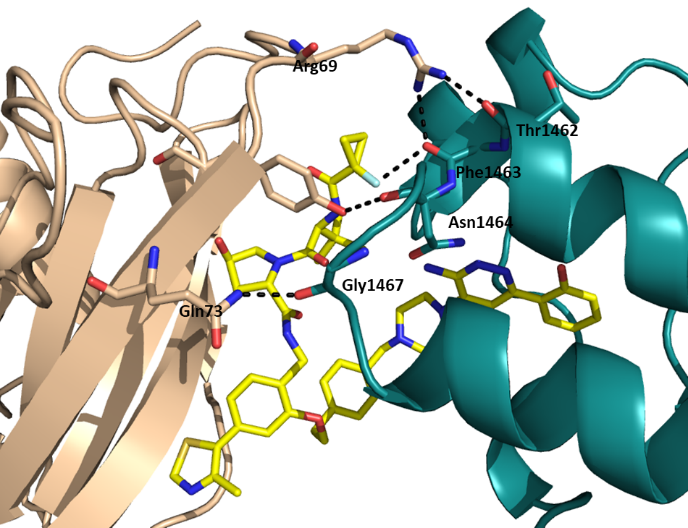


**
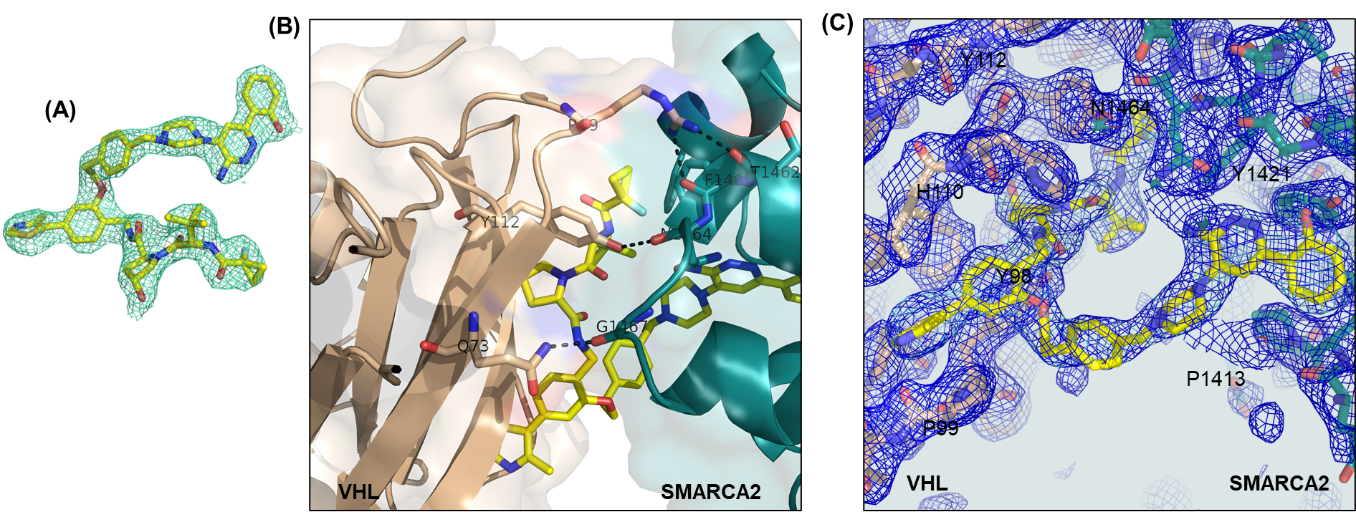
**

**Supplementary Figure 13.** Electron density maps for VBC-Cmpd **11**-SMARCA2 crystals. **(A)** *F_o_-F_c_* omit map (green mesh) of Cmpd **11** in contoured at 3.0 σ. **(B)** Analysis of crystal contacts at VHL-SMARCA2 interface. **(C)** *2F_o_-F_c_* map (blue mesh) of compound **11** (shown in yellow sticks model) in VBC-Cmpd **11**-SMARCA2 contoured at 1.0 σ.

**
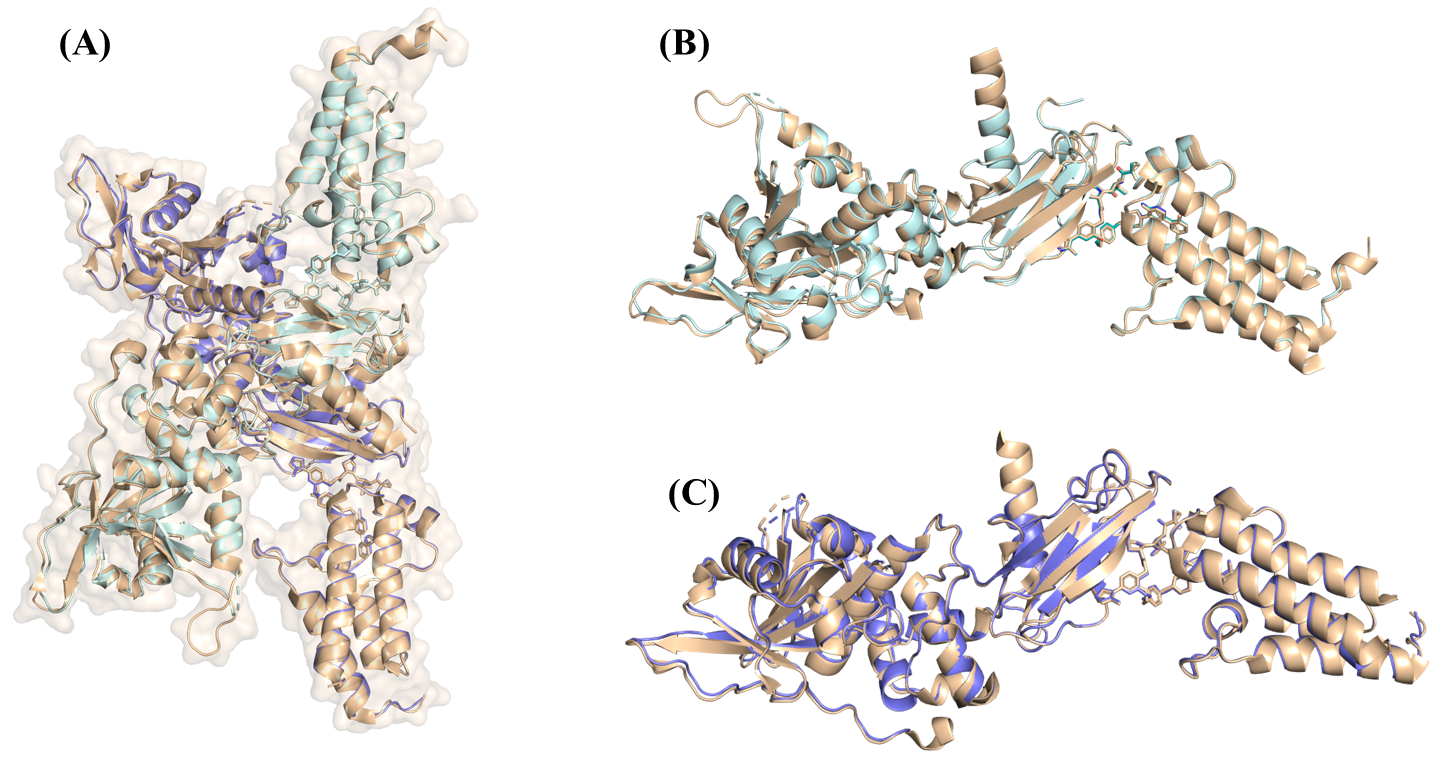
**

**Supplementary Figure 14. (A)** Overview of overlay of 6HAX (literature) and 8G1P (*in-house*) crystal structures with VBC-Cmpd **11**-SMARCA2^BD^. Structures aligned using VHL **(B)** 6HAX copy 1 is shown in wheat and copy of 8G1P structure is shown in pale cyan, **(C)** 6HAX copy 2 is shown in wheat and copy of 8G1P structure is shown in purple.

**Supplementary Table 6.** The pairwise Cα root mean squared deviation (RMSD) in Å between all ternary complex structure copies in the SMARCA2-Cmpd**11**-VBC crystal structures [PDB IDs: 8G1P (*in-house*) and 6HAX (published)]. VBC is used in the structure alignments and the RMSD is computed for the SMARCA2 Cα atoms. The names of the table entries contain the PDB ID followed by the chain IDs for VHL and for SMARCA2 of each structure copy used.

**
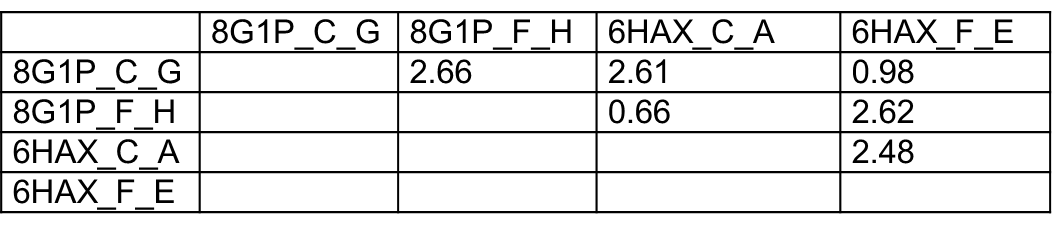
**

| **System** | **Ligase_Target** | **Ligase_ligand** | **Target_ligand** | **Total_BSA (Å^2^)** |
| --- | --- | --- | --- | --- |
| 6HAX | 398.83 | 497.80 | 369.53 | 1266.16 |
| *in-house*  (8G1P) | 388.03 | 482.30 | 385.44 | 1255.77 |
|  |  |  |  |  |

**Supplementary Table 7.** Comparison of calculated BSA from modeling with crystal structures for compound **11** mediated ternary complex.

| BSA of Modeled PROTAC-induced ternary complex structure of compound **11**= 1268 ± 18.5 Å^2^ |  |  |  |  |
| --- | --- | --- | --- | --- |
|  |  |  |  |  |
|  |  |  |  |  |


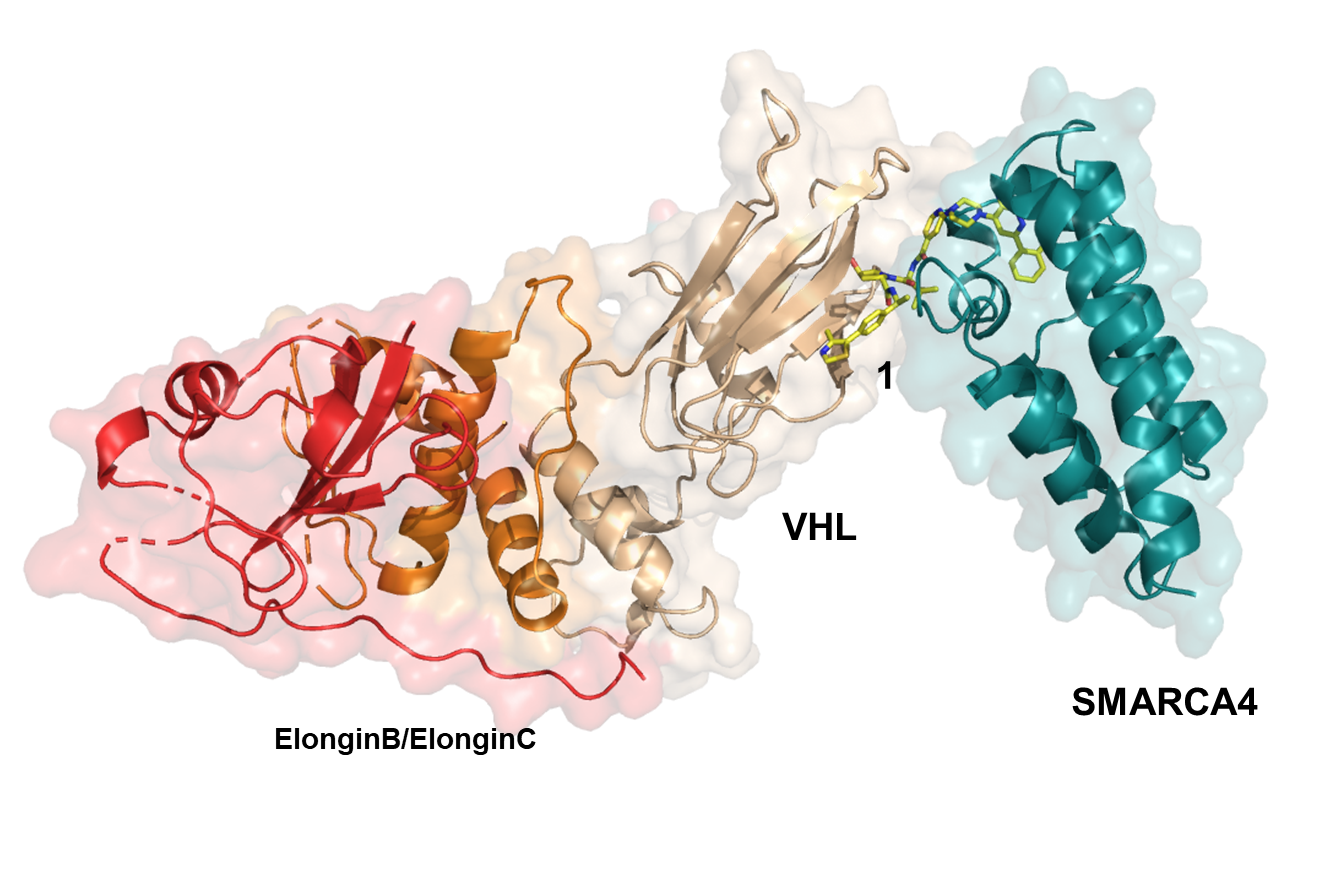


**Supplementary Figure 15.** Crystal structure of VBC-Cmpd **1**-SMARCA4^BD^


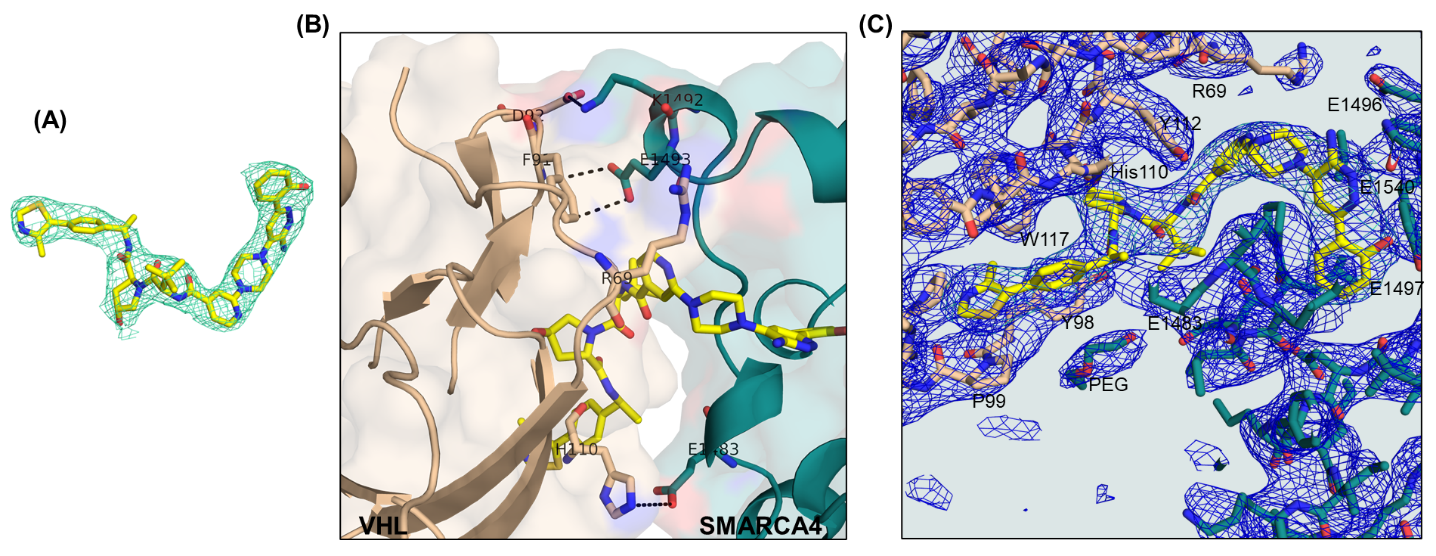


**Supplementary Figure 16.** Electron density maps for VBC-Cmpd **1**-SMARCA4^BD^ crystals. **(A)** *F_o_-F_c_* omit map (green meshes) of compound **1** contoured at 3.0 σ. **(B)** Analysis of crystal contacts at VHL-SMARCA4 interface. **(C)** *2F_o_-F_c_* map (blue meshes) of compound **1** contoured at 1.0 σ.

**Supplementary Note 4:**

**NanoBRET Cellular Ternary Complex Formation Assay for PROTACs Permeability**

We employed a NanoBRET-based target engagement (TE) assay as reported earlier^7^ for evaluating PROTACs relative permeability. In summary, live and permeabilized HEK293 cells with SMARCA2 or BRD4-NanoLuc were used for measuring IC_50_ values of different PROTACs. The relative shift in the IC_50_ was evaluated as relative binding affinity (RBA) by taking the ratio of IC_50_ values of live and permeabilized cells. Availability index (AI), which is a surrogate for permeability can be calculated by taking a ratio of RBA of compounds to permeable benchmark molecules. We used SMARCA bromodomain binding ligand^8^ for SMARCA2 PROTACs and JQ1^9, 10^ for BRD4 PROTACs as benchmark molecules.

Relative Binding Affinity (RBA) = Live-cell TE IC_50_ / Permeabilized-cell TE IC_50_

Availability Index (AI) = RBA_test cmpd_ / RBA_permeable benchmark_

Availability Index (AI) greater than one is indicative of reduced permeability compared to the benchmark.

**Supplementary Table 8. SMARCA2 Degraders Availability Index**

| \| **PROTAC** \| **Live-Cell TE IC_50_ (μM)** \| **Permeabilized-Cell TE IC_50_ (μM)** \| **MW** \| **Availability Index** \| \| --- \| --- \| --- \| --- \| --- \| \| **1** \| 0.20 \| 0.15 \| 818.99 \| 1.31 \| \| **2** \| 0.40 \| 0.20 \| 818.99 \| 1.97 \| \| **3** \| 0.50 \| 0.41 \| 818.99 \| 1.22 \| \| **4** \| 0.82 \| 0.75 \| 818.00 \| 1.10 \| \| **5** \| 1.26 \| 0.40 \| 825.01 \| 3.11 \| \| **6 (AU-15330)** \| 1.41 \| 0.09 \| 755.93 \| 15.46 \| \| **7** \| 0.69 \| 0.21 \| 833.01 \| 3.37 \| \| **8** \| 0.60 \| 0.05 \| 847.04 \| 11.18 \| \| **10** \| 2.37 \| 5.04 \| 803.97 \| 0.47 \| \| **11** \| 0.98 \| 0.74 \| 920.11 \| 1.32 \| |  |  |  |  |
| --- | --- | --- | --- | --- | --- | --- | --- | --- | --- | --- | --- | --- | --- | --- | --- | --- | --- | --- | --- | --- | --- | --- | --- | --- | --- | --- | --- | --- | --- | --- | --- | --- | --- | --- | --- | --- | --- | --- | --- | --- | --- | --- | --- | --- | --- | --- | --- | --- | --- | --- | --- | --- | --- | --- | --- | --- | --- | --- | --- |

**Supplementary Table 9. BRD4 Degraders Availability Index**

| **PROTAC** | **Live-Cell TE; IC_50_ (μM)** | **Permeabilized-Cell TE; IC_50_ (μM)** | **MW** | **Availability Index** |
| --- | --- | --- | --- | --- |
| **12** | 21.61 | 0.08 | 995.61 | 264.47 |
| **13** | 7.27 | 0.10 | 1039.66 | 73.87 |
| **14** | 12.64 | 0.10 | 1083.72 | 126.63 |
| **15** | 3.92 | 0.11 | 1127.77 | 34.23 |
| **16** | 18.03 | 0.11 | 1171.82 | 164.81 |
| **17 (MZ1)** | 4.28 | 0.08 | 1002.65 | 54.34 |

**Supplementary Note 5:**

**Abbreviations**

| UPS | Ubiquitin Proteasome System |
| --- | --- |
| PROTAC | Proteolysis Targeting Chimera |
| SAR | Structure-Activity Relationship |
| DR | Dose-Response |
| SPR | Surface Plasmon Resonance |
| PPI | Protein-Protein Interaction |
| BSA | Buried Surface Area |
| *L* | Ligase |
| *T* | Target |
| *P* | PROTAC |
| *K_LPT_* | Equilibrium Dissociation Constant of Ternary Complex Involving *L*, *P* *and* *T* |
| *K_LP_* | Equilibrium Dissociation Constant of Binary Complex Involving *L and* *P* |
| *K_TP_* | Equilibrium Dissociation Constant of Ternary Complex Involving *T* *and P* |
| *α* | Cooperativity (*K_LP_/K_LPT_*) |
| *ν* | Target Ubiquitination Initial Rate |
| *V_max_* | Product of Ternary Complex Breakdown Rate Constant and Total Ligase Concentration |
| AUC | Area Under the Curve |
| DC_50_ | Concentration of a PROTAC at which the cellular protein content is reduced by half |

**Supplementary References**

1. Douglass, E.F., Miller, C.J., Sparer, G., Shapiro, H. & Spiegel, D.A. A comprehensive mathematical model for three-body binding equilibria. *J. Am. Chem. Soc.* **135**, 6092-6099 (2013).

2. Lu, C. & Wang, Z.-X. Quantitative Analysis of Ligand Induced Heterodimerization of Two Distinct Receptors. *Anal. Chem.* **89**, 6926-6930 (2017).

3. Karlsson, R. Biosensor binding data and its applicability to the determination of active concentration. *Biophys. Rev.* **8**, 347-358 (2016).

4. Squires, T.M., Messinger, R.J. & Manalis, S.R. Making it stick: convection, reaction and diffusion in surface-based biosensors. *Nat. Biotechnol.* **26**, 417-426 (2008).

5. Glaser, R.W. Antigen-Antibody Binding and Mass Transport by Convection and Diffusion to a Surface: A Two-Dimensional Computer Model of Binding and Dissociation Kinetics. *Anal. Biochem.* **213**, 152-161 (1993).

6. Annunziata, O., Buzatu, D. & Albright, J.G. Protein Diffusion Coefficients Determined by Macroscopic-Gradient Rayleigh Interferometry and Dynamic Light Scattering. *Langmuir* **21**, 12085-12089 (2005).

7. Riching, K.M. *et al.* Translating PROTAC chemical series optimization into functional outcomes underlying BRD7 and BRD9 protein degradation. *Curr. Res. Chem. Biol.* **1**, 100009 (2021).

8. Farnaby, W. *et al.* BAF complex vulnerabilities in cancer demonstrated via structure-based PROTAC design. *Nat. Chem. Biol.* **15**, 672-680 (2019).

9. Chan, K.-H., Zengerle, M., Testa, A. & Ciulli, A. Impact of target warhead and linkage vector on inducing protein degradation: comparison of bromodomain and extra-terminal (BET) degraders derived from triazolodiazepine (JQ1) and tetrahydroquinoline (I-BET726) BET inhibitor scaffolds. *J. Med. Chem.* **61**, 504-513 (2018).

10. Wurz, R.P. *et al.* A “Click Chemistry Platform” for the rapid synthesis of bispecific molecules for inducing protein degradation. *J. Med. Chem.* **61**, 453-461 (2018).
